# *In vivo* interferon-gamma induced changes in gene expression dramatically alter neutrophil phenotype

**DOI:** 10.1371/journal.pone.0263370

**Published:** 2022-02-03

**Authors:** Daniel R. Ambruso, Natalie J. Briones, Angelina F. Baroffio, John R. Murphy, Alexander D. Tran, Katherine Gowan, Bridget Sanford, Michael Ellison, Kenneth L. Jones

**Affiliations:** 1 Department of Pediatrics, University of Colorado School of Medicine, Aurora, Colorado, United States of America; 2 Center for Cancer and Blood Disorders, Children’s Hospital Colorado, Aurora, Colorado, United States of America; Leiden University Medical Center, NETHERLANDS

## Abstract

The cytokine Interferon-γ (IFN-γ) exerts powerful immunoregulatory effects on the adaptive immune system and also enhances functions of the neutrophil (PMN). The clinical use of IFN-γ has been driven by the finding that its administration to patients with chronic granulomatous disease (CGD) results in decreased incidence and severity of infections. However, IFN-γ has no effect on the characteristic defect of CGD, the inability to convert oxygen to microbicidal metabolites including superoxide anion (O_2_^-^) during the phagocytosis associated oxidative burst. We administered varying doses of IFN-γ to adult volunteers and studied the effects on plasma drug levels and response molecules and PMNs isolated from blood drawn at intervals over a 96- hour period. Plasma concentrations of IFN-γ, IP-10 and neopterin, and stimulated release of O_2_^-^ from PMNs exhibited dose- and time-dependent increases after IFN-γ administration. Gene expression in PMNs was altered for 2775 genes; changes occurred rapidly after administration and returned to baseline in 24–36 hours. Several genes involved with neutrophil host defense were upregulated including those for components of the O_2_^-^ generating NADPH oxidase; innate-immune and Fc receptors; proteins involved in MHCI and II; a regulator of circulating PMN number; guanylate binding proteins; and a key enzyme in synthesis of an essential NOS cofactor. Coordinate changes were detected in protein levels of representative products from several of these genes. Lysates from isolated neutrophils also demonstrated a spike in NO following IFN-γ administration. IFN-γ appears to increase non-oxygen dependent microbicidal functions of PMNs which could provide strategies to compensate for deficiencies, explain its clinical benefit for CGD patients and expand therapeutic applications of IFN-γ to other disorders.

**Trial registration:** Protocol registered in ClinicalTrials.gov, NCT02609932, Effect of IFN-γ on Innate Immune Cells.

## Introduction

Named for their potent ability to interfere with viral infections, interferons (IFNs) are powerful regulators of the immune system [1, 2]. Of the members of the two classes of these compounds, IFN-γ has the strongest and most diverse effects. This dimerized cytokine is predominantly secreted by CD8+ T cells, CD4+ T helper 1 cells, and natural killer cells and to a lesser extent by macrophages, dendritic cells, and B cells [1, 2]. IFN-γ exerts its many effects by interaction with the cell surface receptor IFN-γR which activates signaling pathways that include the canonical JAK-STAT pathway [3]. IFN-γ powerfully modulates expression of multiple genes producing a variety of physiological and cellular responses [3]. Studies have mostly evaluated IFN-γ interactions with cells of adaptive immunity, and its effects on innate immunity, including on PMNs, are less well studied. However, it has been demonstrated that IFN-γ alters myeloid cell activity in diverse ways including, increased MHCII protein expression, altered cytokine and chemokine production, enhanced NADPH oxidase activity/oxidative burst, increased nitric oxide (NO) production, increased phagocytosis and cytocidal effects, increased Fc receptor expression, inhibition of chemotaxis and suppression of apoptosis [4]. Although most of these changes have been documented as *in vitro* effects on mature neutrophils, recent studies show that more dramatic changes occur when IFN-γ interacts with maturing myeloid cells [5].

The early findings of certain enhancements of neutrophil function led to a clinical trial of IFN-γ in patients with CGD, a group of genetic disorders characterized by defects in components of the NADPH oxidase [6], a multiprotein, transmembrane complex that generates large quantities of superoxide anion (O_2_^-^) in response to specific agonists or phagocytosis (the oxidative burst). A clinical trial evaluating IFN-γ in CGD demonstrated a reduction in the number and severity of infections [7]. This was confirmed by a second study [8] and IFN-γ is now accepted as an important management strategy for this disorder.

In spite of the clinical benefits of IFN-γ in CGD, there is no understanding of the precise mechanism for its effects. In recent studies from our laboratory using a myeloid cell culture model, we demonstrated dramatic changes in NADPH oxidase protein expression and enzyme activity when IFN-γ was present during the maturation of the cells [5, 9]. Documenting the phenotype of neutrophils which have developed under the influence of this cytokine is critical to understanding how it is beneficial in CGD and for predicting disease states other than CGD where it might be of value. To expand our understanding of the role of IFN-γ in the functional integrity of the neutrophil, we studied its *in vivo* effects on neutrophils from healthy adult volunteers.

## Materials and methods

### Study design

The data presented in this manuscript are results for one of two cohorts of subjects focused on the *in* vivo effects of IFN-γ on normal individuals. This total protocol was approved first by the Scientific Advisory and Review Committee for scientific review at the Colorado Clinical Translational Sciences Institute and then by University of Colorado COMIRB for human subjects review before initiation of research activities. As part of the scientific review, issues such as sample size and power analysis as well as other aspects of the protocol noted below were reviewed and approved. These issues were described in the COMIRB protocol which accompany this submission. We hypothesized that neutrophils developed under the influence of Interferon- gamma-1b (IFN-γ) in vivo would display enhanced function across a broad range of activities related in large part to the transcriptional activation effects of this cytokine. The primary objective of this study was to better define in vivo timing of transcriptional effects of IFN-γ on neutrophils from healthy subjects, and the secondary objective was to characterize the effects of IFN-γ on function and biochemistry of neutrophils under the influence of this cytokine. The focus of the specific cohort described in this manuscript was to evaluate four separate doses of IFN-γ described below.

Taking into consideration attrition and potential dropout rates, up to thirty (30) healthy adult volunteers would be screened and enrolled in both cohorts of this study. A total of ten (10) subjects would be enrolled on the cohort receiving separate single doses (single dose, SD, cohort) and the potential of up to twenty (20) subjects would be enrolled on the second cohort. The SD cohort was the first to enroll and is the subject of this manuscript. Participants were recruited locally on the University of Colorado, Anschutz Medical Campus through COMIRB-approved flyers distributed throughout campus, as well as listings on campus-wide “Available Clinical Trials” emails and website. Informed consent was obtained in a face-to-face meeting with the principal investigator in a confidential examining room in the Clinical Translational Research Institute’s Clinic at University Hospital under routine, COMIRB approved conditions.

Subjects of the SD cohort received doses of IFN-γ by subcutaneous injection in right or left deltoid, or anterior thighs administered by nursing staff in the CCTSI Clinic on Weeks 1, 5, 9 and 13 respectively. Due to the blood volume required for the SD Cohort studies and to allow adequate time for drug washout and hematopoietic cell recovery, four weeks were allowed before administering the next IFN-γ dose to the same study subject. The study extended from December 10, 2015 until January 9, 2017. IFN-γ was provided by Horizon Pharma Ireland Ltd. to study participants at no cost.

### Clinical protocol

**Fig 1:**
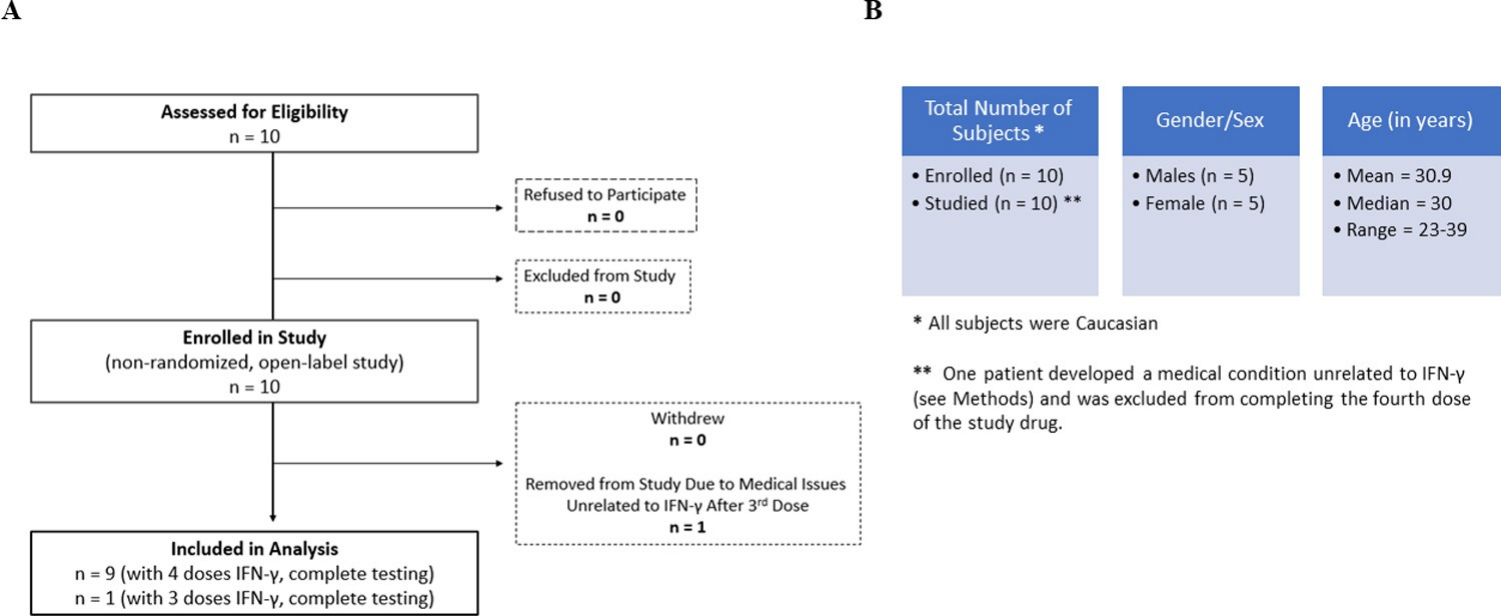
A. Flow diagram showing recruitment, enrollment, and analysis in the clinical trial and B. Demographics for the patients enrolled in the study.

Ten healthy human adult volunteers between 18 and 60 years of age were eligible for the study. Each subject gave written informed consent prior to inclusion in the study. The study protocol was approved by the Scientific Advisory and Review Committee at the Colorado Clinical Translational Sciences Institute and the COMIRB at the University of Colorado Denver, Anschutz Medical Campus and certified under an IND by the FDA. The protocol was also registered on clinicaltrials.gov as NCT02609932, Effect of IFN-γ on Innate Immune Cells. A flow chart for the trial is shown in Fig 1A. Five males and five females (see Fig 1B. Demographics for the study subjects) with no chronic medical conditions or medications, history of infections in the previous two weeks or recurrent infections in the past several months were enrolled on the protocol after confirming normal blood counts (CBC) and negative pregnancy test for the women of childbearing age. Interferon-gamma-1b (IFN-γ, Horizon Pharma Ireland Ltd.), at single, escalating doses of 10, 25, 50, and 100 mcg/m^2^, was given subcutaneously; and blood samples (25ml) were obtained just before (time 0) and 4, 8, 12, 24, 36, 48, 72, and 96 hours after administration for testing described below.

The toxicity profile for IFN-γ is low and has been well defined since its use in patients with CGD established in 1991. The clinical safety of IFN-γ has been extensively studied both as a single agent and as adjunctive treatment, in normal volunteers and in patients with diverse medical conditions. Both adult and pediatric populations with and without CGD have been studied, and multiple routes of administration have been evaluated including the subcutaneous route used here. In all these studies, there was no evidence of irreversible clinical laboratory abnormalities that were considered by the investigators. The incidence of adverse events (AEs) is low. Hypersensitivity reactions to IFN-γ, E. coli derived constituents, or components of the product are rare. Severe cardiovascular or neurologic disorders or bone marrow and liver toxicities have been seen at daily doses two and a half times greater than the single dose or three times a week dosing described in this protocol. The most commonly reported adverse events included mild fever, headache, rash, chills, and injection site erythema and tenderness. These AEs decreased in severity and frequency as IFN-γ treatments continued and were ameliorated by administration of acetaminophen. Other symptoms such as fatigue, diarrhea, vomiting, nausea, myalgia, and arthralgia occurred at the same rate as placebo or were related to the underlying disorder and/or its complications.

No serious adverse events occurred during this study. Non-serious, mild adverse events included mild headaches, fatigue, body aches, low grade fever, and chills in several subjects. These were possibly IFN-γ related, common and anticipated events that resolved on their own or after medications were taken. Other events occurred during the study including individual episodes of mild nausea (2), mild dizziness (2), mild shortness of breath (1) and mild phlebitis (1) all associated with phlebotomy and resolving promptly with supportive care.

During the study, one subject reported pituitary dysfunction with testosterone deficiency diagnosed by his primary care physician. Because he no longer met inclusion criteria, this subject was removed from the study after three doses of IFN-γ. Review of the available medical records by an endocrinologist did not confirm the diagnosis of pituitary disorder or suggest any relationship to his endocrine disorder by IFN-γ administration (see Fig 1).

### Experimental procedures

EDTA anticoagulated plasma was separated immediately from blood cells by centrifugation at 15,000 X g and stored at -80°C. Plasma levels of IP-10 and the small molecule metabolite neopterin, were determined by ELISA techniques (IP-10, ELISA kit from RayBiotech [catalogue number ELH-IP10] and neopterin, ELISA kit from IBL International [catalogue number RE59321]). Plasma IFN-γ was measured commercially by KCAS Bioanalytical and Biomarker Services (Kansas, City, KA). Neutrophils were isolated from heparinized whole blood by Dextran sedimentation, Ficoll Hypaque density gradient centrifugation, and hypotonic lysis of red blood cells [10, 11]. Isolated neutrophils were quantitated manually in a hemocytometer in the presence of 3% paraformaldehyde. Superoxide anion (O_2_^-^) generation by neutrophils, after stimulation with phorbol myristate acetate (PMA, 200ng/ml) and formyl-methionyl-leucyl-phenylalanine (fMLF, 1μM), was measured as SOD inhibitable cytochrome c reduction [10, 11].

RNA was isolated from neutrophils using the RNeasy Mini Kit (Qiagen). At the Genomics and Microarray Core of the University of Colorado, Anschutz Medical Campus, the RNA was used to prepare labelled cDNA which was hybridized to human transcript microarrays (HuGene2.0ST, Affymetrix). Further processing at the core facility generated.CEL files for each microarray as described before [5]. Bioconductor in R (oligo package) was used to extract and normalize expression from the.CEL files and repeated measurements of ANOVA in R were used to evaluate whether gene expression changes were significant over time (Time-p) and dose (Dose-p) and, ultimately, the interaction of time x dose. As each subject’s time zero baseline was inherently different, we subtracted the expression level at time zero from each subsequent time point to determine their differences in expression from baseline. This difference from baseline was then used for downstream statistics. We required the significance for each gene to be at a level of p<1.76e-6 (i.e., p<0.05 with strict Bonferroni correction for 28463 genes). A 2-fold cutoff in gene expression was used as a practical change for purposes of this analysis. We determined that at the cutoff of 2, in comparison to 0, 1.5, or 4, the significance of upstream regulator analysis was minimized using the Ingenuity Pathways Analysis package (Qiagen). The microarray data from this study have been deposited into Array Express database at EMBL-EBI (www.ebi.ac.uk/arrayexpress) under accession number E-MTAB-7261. For the 2775 genes significant at the 2-fold cutoff, a heatmap was generated showing the average change in expression across all subjects at each time point and dose. Genes showing significant changes in Time-p or Dose-p are listed in S1 Table and were evaluated to determine if any correlation to neutrophil function could be discerned.

At the various time points, isolated neutrophils were pelleted, and lysates produced (10^8^ cells/ml) [9–11]. Nitric oxide (NO) levels in neutrophil lysates were inferred using a colorimetric assay (In Vitro Nitric Oxide (Nitrite/Nitrate) Assay Kit from Cell Biolabs Inc, San Diego, CA) to measure the combined nitrite and nitrate levels, and tetrahydrobiopterin (BH4) was determined with an avidin/biotin based competitive ELISA kit (Aviva systems Biology, San Diego, CA). Protein concentrations in the cell lysates were measured using the Pierce BCA Protein assay kit (catalogue number 23225) and nitrite plus nitrate as well as BH4 concentrations were normalized to these values. Western blot analysis of lysates (50 μg protein) was completed with resolution of proteins on 10% or 15% SDS-PAGE and blotting onto nitrocellulose membranes as is described in detail [5, 9–11]. Detection of proteins on membranes was performed with exposure to primary antibodies as follows, for FCGR1A (mouse monoclonal, Origene, Rockville, MD, USA), FCGR1B (rabbit polyclonal, ThermoFischer, Rockfort, IL or Invitrogen, Waltham, MA, USA), GBP1 (rabbit polyclonal, Proteintech, Rosemont, IL, USA), GCH1 (mouse monoclonal, ThermoFischer, Rockfort, IL or Invitrogen, Waltham MA, USA), gp91^*phox*^ (mouse monoclonal, Santa Cruz Biotechnology, Santa Cruz, CA, USA), HLA-DMB (rabbit monoclonal, Abcam, Cambridge, UK), MD2/LY96 (Invitrogen, Waltham, MA, USA), p47^*phox*^ (goat polyclonal, Abcam, Cambridge, UK), TLR4 (rabbit polyclonal, Bio-techne, Minneapolis, MN, USA), and GAPDH (mouse monoclonal, Origene, Rockville, MD, USA). Secondary antibodies included Goat anti-Mouse IgG Horse Radish Peroxidase (Cayman Chemical, AnnArbor, MI, USA), and Goat Anti-Rabbit IgG H&L Horse Radish Peroxidase (Abcam, Cambridge, UK) and Donkey anti-Goat IgG (Abcam, Cambridge, UK). Immune complexes were then detected with an enhanced chemiluminescence system from Amersham ECL western Blotting Reagents (GE Healthcare). Alternatively, Western nitrocellulose membrane blots were analyzed using the G:BOX Chemi XL1.4 Fluorescent & Chemiluminescent Imaging System and CAM-GX-CHEMI-XL1.4 Camera Lens Assembly (Syngene, Cat# 05-GBOX-CHEMI-XL1.4 (G:BOX Chemi XL1.4)/ Cat# 05-CAM-GX-CHEMI-XL1.4, Frederick, Maryland, USA) and associated software (Genesys software version 1.8.2.0). Quantitation of proteins detected by Western blotting was done with densitometry using ImageJ software (http://imagej.nih.gov/ij/). Western blots contained samples from an individual patient’s lysate samples before and after adminidtration of IFN-γ. Quantitation of the densitometry measurements for specific proteins was completed by calculating the ratio of the specific time after administration by the result from before administration. This represented the fold change over time and results were summarized as mean ± SEM for the number of separate measurements completed.

### Statistical methods

As no data existed for in vivo neutrophil gene expression, function, or biochemical data that were measured in this study, we used our previous *in vitro* culture model [5, 9] that measured gene expression, Nox2 activity and phox protein levels to power this study. The difference in Nox2 activity and *phox* protein levels between IFN-γ treated and controls in these previous studies were at least 2.5-fold of the common standard deviation. With 10 subjects serving as their own controls, the study provides 95% power to detect a difference of 1.3 common standard deviations between baseline and post-IFN-γ treatment at relevant time points with a two-sided paired t-test with a significance level of 0.05. Thus, to be conservative to assume that human samples are more variable yet using the same subject as their own control reduces variability, it appears that 10 subjects for the studies proposed would provide interpretable results.

Study outcomes included: IFN-γ, IP-10, neopterin, PMA and fMLF stimulated superoxide anion (O_2_^-^), nitrate plus nitrite (NO), tetrahydrobiopterin (BH4), quantitation of specific proteins by Western blot, and gene expression over time and dose. Average levels of IFN-γ, IP-10, neopterin, PMA and fMLF stimulated O_2_^-^ production, nitrate plus nitrite, and BH4 and protein levels were presented along with standard error bars. Differences in levels of each outcome at each time point were compared to time zero using paired t-tests. Repeated measurements analysis of variance (RM ANOVA) was used to evaluate whether gene expression significantly changed over time and dose. As each subject’s time zero baseline was inherently different, the expression level at time zero was subtracted from each subsequent time point to determine their differences and this difference from baseline was then used for RM ANOVA as described above in RNA techniques. The significance for each gene was required to be at a level of p<1.76e-6 (i.e., p<0.05 with strict Bonferroni correction for 28463 genes). A 2-fold cutoff in gene expression was used as a practical change for purposes of this analysis. For genes significant at the 2-fold cutoff, a heatmap was generated showing the average change in expression across all subjects at each time point and dose.

Microsoft Excel for Microsoft 365 (64-bit) with "Analysis ToolPak" software was used for all analysis except gene expression. The overall significance level was set at 0.05. Except for the previously mentioned Bonferroni correction for the gene expression analysis, adjustments for multiple comparisons were not performed, as this was a pilot study of exploring IFN-γ in human subjects. The emphasis of the results is on the effect sizes.

## Results

### Plasma levels of IFN-γ and response molecules

**Fig 2:**
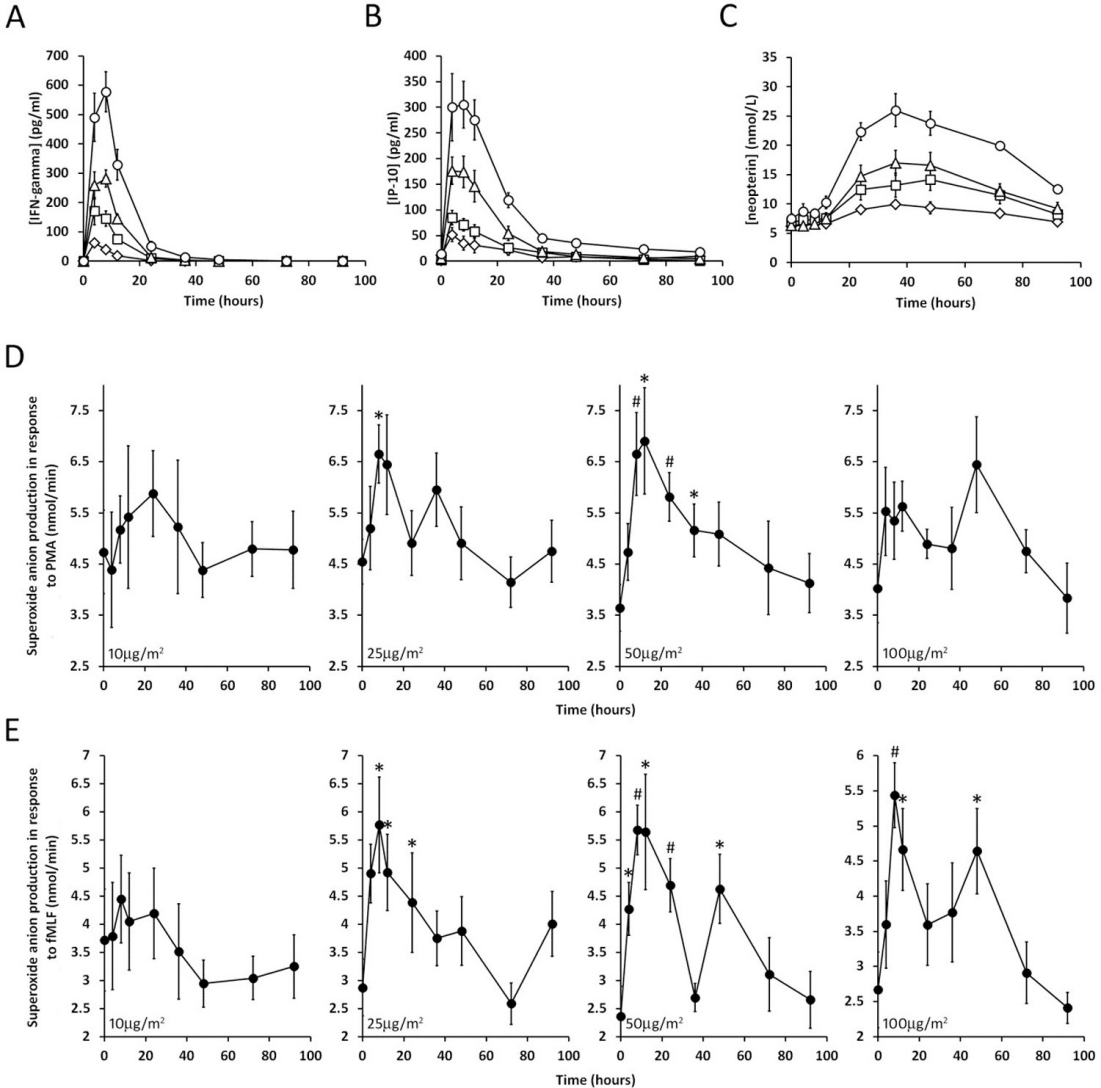
A, B and C: IFN-γ, IP-10 and neopterin levels (respectively) in plasma of healthy volunteers. IFN-γ was administered just after blood was taken for the time 0 time point. The responses to different concentrations are indicated by different symbols (10μg/m^2^, diamonds; 25μg/m^2^, squares; 50μg/m^2^, triangles and 100μg/m^2^, circles). The error bars are SEM for n ≥7. D and E: Superoxide anion production by neutrophils. At the same time points as in A, B and C, neutrophils were isolated from the peripheral blood of healthy volunteers and superoxide anion production was stimulated by PMA (D) and fMLF (E). The error bars are SEM for n ≥7. The asterisks and pound signs indicate differences (p = 0.008–0.043 and p = 0.001–0.004 respectively) from time 0 (two tailed t-test).

Following administration of IFN-γ, peaks in its plasma levels were seen after 4–8 hours (Fig 2A) with return to baseline by 12–36 hours.

IP-10 (interferon gamma-induced protein 10/C-X-C motif chemokine 10) is a small cytokine whose elevated expression in response to IFN-γ is well characterized [12]. Levels of IP-10 (Fig 2B) paralleled those of IFN-γ, but it tended to persist for slightly longer than those of IFN-γ.

Neopterin is a small molecule metabolite in the Biopterin pathway that is also known to be upregulated in response to IFN-γ [13]. Its plasma levels over time following IFN-γ administration were very distinct from IFN-γ and IP-10; the elevated levels of neopterin developed more slowly and persisted for longer (Fig 2C).

### Neutrophil oxidative burst

PMA and N-formyl-methionyl-leucyl-phenylalanine (fMLF)-induced superoxide anion generation by neutrophils was measured using cytochrome c reduction (Fig 2D and 2E). For both agonists, IFN-γ caused a rapidly appearing peak in O_2_^-^ production that increased in magnitude as the dose of IFN-γ increased up to 50μg/m^2^ dose. However, at 100μg/m^2^, the initial rapidly-induced-peak, decreased relative to 50μg/m^2^ and a second, later peak appeared. For fMLF mediated O_2_^-^ production, a second later peak was also present in the 50μg/m^2^ time course.

### Gene expression data

**Fig 3:**
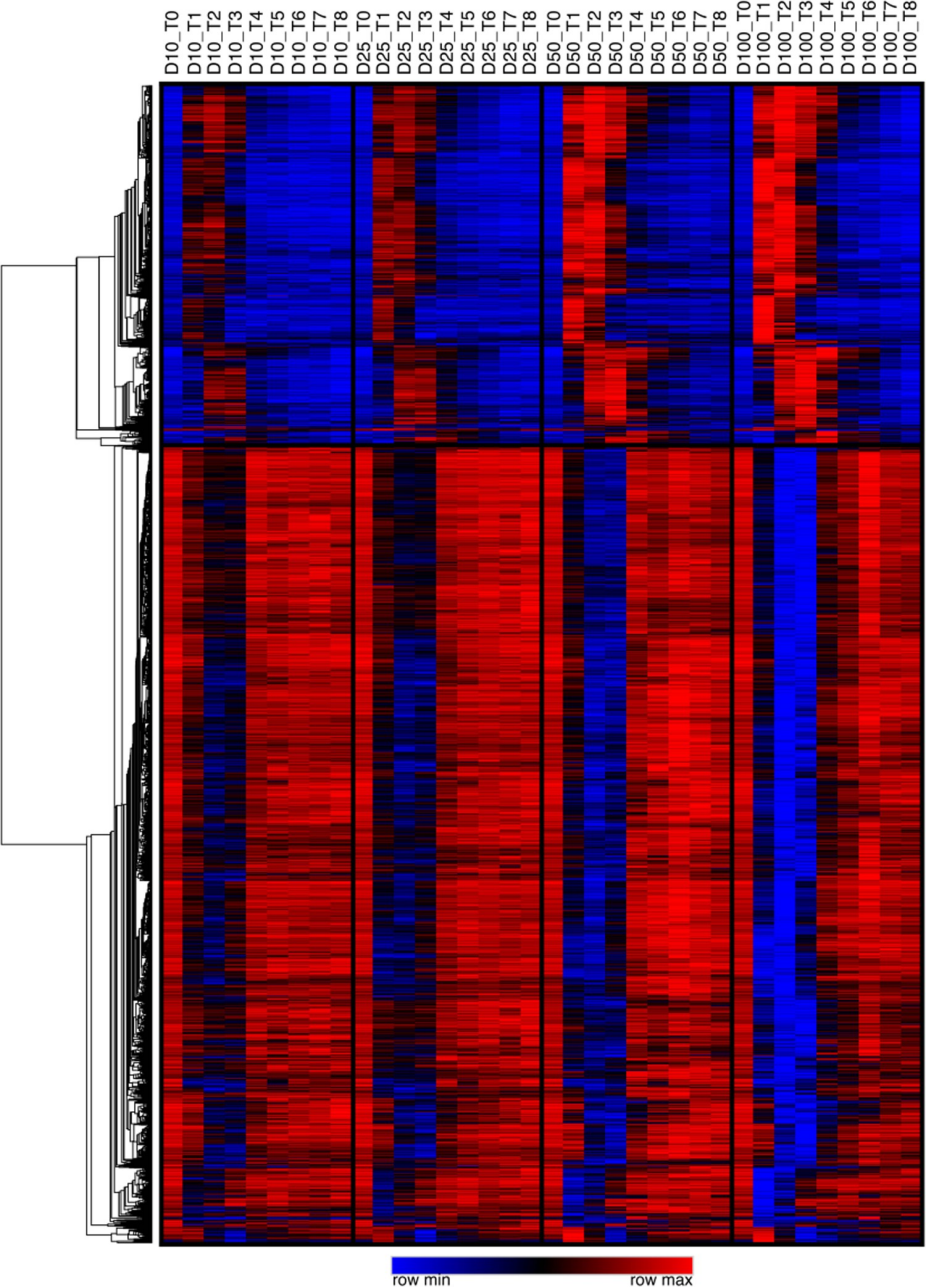
Heat map showing time dependent changes for all genes with a ≥ 2-fold maximal increase or decrease in gene expression (mRNA) in response to single doses of IFN-γ. Gene expression for each gene averaged across subjects are shown in order from pre (T0) or post (T1-8) IFN-γ administration as well as by dose (10, 25, 50 and 100mcg/m^2^ respectively). The cells are colored according to expression level relative to row (i.e., relative within gene). Genes that responded to IFN-γ with increased expression are at the top and those that showed decreased expression are at the bottom.

**Fig 4:**
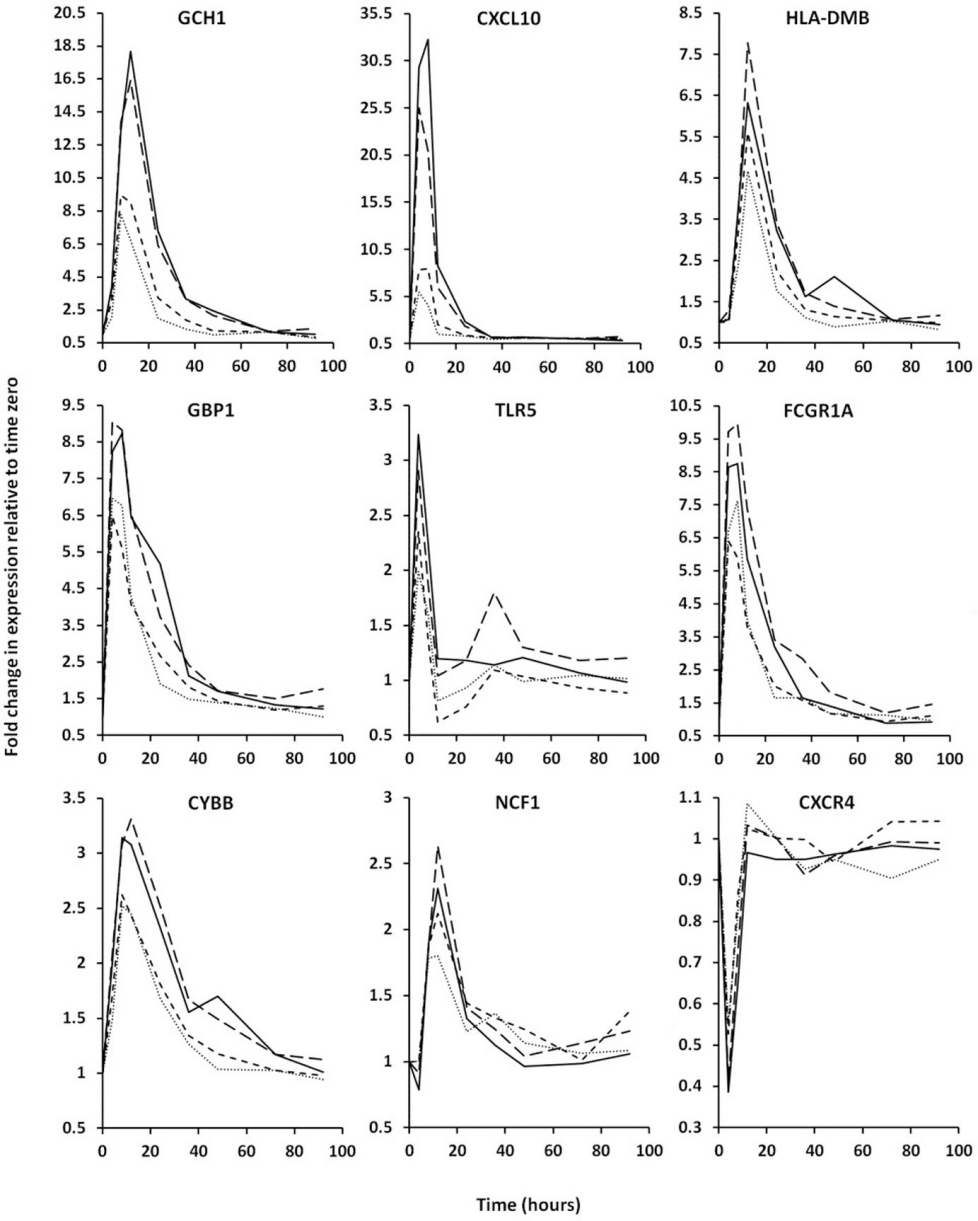
Examples of gene expression changes in response to IFN-γ administration. The fold changes relative to expression levels just prior to IFN-γ administration (time 0) are plotted against the various times at which blood was drawn. The selected genes are discussed in more detail throughout the manuscript. The doses of IFN-γ causing expression changes are indicated by line style (10μg/m^2^, dots; 25μg/m^2^, short dashes; 50μg/m^2^, long dashes; 100μg/m^2^, solid line). Raw expression data from individual genes are shown in S1 Table.

After administration of IFN-γ to volunteers, time dependent changes in the genome wide mRNA levels of their neutrophils were measured using Affymetrix gene chips. The heat map in Fig 3 summarizes the changes in expression of all genes which experienced a 2-fold or greater increase or decrease in expression at one or more time points. As can be seen, the genes responded to IFN-γ with a sharp trough of decreased expression or a sharp peak of increased expression; these generally increase in magnitude and duration with increasing IFN-γ dose. In S1 Table we show all genes with IFN-γ mediated expression changes that were significant over time (Time-p<0.05) or that changed significantly at the different doses of IFN-γ administered (Dose-P<0.05). By these criteria, 866 genes show transient expression increases and 1909 exhibit transient expression decreases following IFN-γ administration. In addition, Fig 4 shows IFN-γ-mediated expression profiles for selected genes.

To understand how treatment with IFN-γ might enhance neutrophil function, we examined the gene list (S1 Table) to see if any relation to known neutrophil activity could be discerned. Genes identified in this way are listed in Table 1 where they are organized into protein families or groups of genes associated with specific neutrophil functions. We also used Ingenuity pathway analysis to identify any potentially relevant canonical pathways in this collection of expression changes (Table 2). The altered neutrophil functions and corresponding genes and their proteins identified by these two methods are described below. Collectively they reflect marked changes in the neutrophil phenotype.

**Table 1:** Functionally relevant neutrophil genes that were altered by IFN-γ administration to healthy subjects.

Gene	Fold change	Time-P	Dose-P
Antigen Presentation			
HLA-DMB	7.77	6.56E-26	0.00139
HLA-DMA	5.81	1.14E-26	0.00140
HLA-DRA	3.67	2.63E-19	0.00769
HLA-DOB	3.41	3.56E-29	2.25E-05
HLA-DQB2	2.72	2.16E-15	0.000859
HLA-DRB4	2.55	1.50E-08	0.165
HLA-DPA1	2.36	7.75E-09	0.633
HLA-DQB1	2.27	3.04E-11	0.0893
HLA-DQA2	2.24	4.21E-07	0.0708
HLA-DPB1	2.14	1.52E-09	0.262
CIITA	4.11	1.60E-28	1.16E-05
RFX5	3.62	4.65E-29	0.0448
CD74	2.89	1.56E-23	0.000669
CD40	2.86	1.21E-09	0.206
PDCD1LG2	16.5	2.57E-31	0.00233
CD274	17.0	1.22E-30	5.02E-05
TAP1	3.14	5.16E-31	0.000268
TAP2	3.69	1.71E-31	5.24E-06
PSMB8	2.11	1.15E-28	1.50E-05
PSMB9	2.47	7.82E-24	0.0287
Guanylate Binding Proteins			
GBP1	9.05	2.09E-28	0.000670
GBP2	2.26	1.61E-26	0.00403
GBP3	11.4	5.10E-28	0.0193
GBP4	13.1	2.05E-26	0.00101
GBP5	5.55	1.64E-25	0.0512
GBP6	12.7	5.33E-26	0.00280
GBP7	2.47	1.77E-09	0.000105
GBP1P1	20.1	6.81E-29	0.000402
Innate Immune Receptors			
TLR4	1.28	4.65E-09	0.0123
TLR5	3.24	2.08E-17	0.0945
LY96	2.51	5.51E-21	0.563
TLR8	2.06	1.79E-26	0.0578
NOD1	2.78	1.74E-19	0.147
CLEC4D	2.13	3.45E-07	0.0738
CLEC5A	2.56	1.60E-14	0.00483
CLEC6A	2.83	1.01E-12	0.338
CLEC9A	3.16	1.74E-24	0.000206
Fcγ Receptors			
FCGR1A	9.96	4.20E-26	0.000227
FCGR1B	6.92	5.70E-29	5.62E-05
NADPH Oxidase Proteins			
CYBB	3.31	4.64E-25	0.00191
NCF1	2.63	6.49E-20	0.336
GTP Cyclohydrolase 1			
GCH1	18.2	1.15E-30	0.00234
C-X-C Motif Chemokine Receptor 4			
CXCR4	‒2.59	1.84E-23	0.0201

Fold change is the maximum change in gene expression seen at any time point and IFN-γ dose relative to time 0 (i.e. just prior to administration of IFN-γ). Time-p is a measure of the statistical significance of a given gene’s expression changing with time and Dose-p is a measure of the statistical significance of the response being different at the various doses of IFN-γ.

**Table 2:** Pathways analysis applied to the gene expression changes induced by IFN-γ in neutrophils of healthy subjects.

Ingenuity Canonical Pathways	p-value
Antigen Presentation Pathway	5.10E-10
Th1 and Th2 Activation Pathway	1.26E-8
Th1 Pathway	2.95E-8
Role of Pattern Recognition	1.26E-6
Receptors in Recognition of Bacteria and Viruses	
Th2 Pathway	1.74E-6

The names of altered canonical pathways which have a discernable connection to neutrophil function are listed with the corresponding p values for the significance of their changes.

#### MHCII system

The Ingenuity pathway analysis (Table 2) identified “Antigen Presentation Pathway”, “Th1 and Th2 Activation Pathway”, “Th1 Pathway” and “Th2 Pathway” as among the most statistically significant canonical pathways. The genes in these pathways that were upregulated by IFN-γ administration include many of the MHCII antigen presenting proteins (HLA-DMB, HLA-DMA, HLA-DRA, HLA-DOB, HLA-DQB2, HLA-DRB4, HLA-DPA1, HLA-DQB1, HLA-DQA2, HLA-DPB1) as well as CIITA and RFX5 which together enhance transcription of proteins of the MHCII system. All exhibit a significant difference over time and most show a dose dependency (Table 1, Fig 4, S1 Table). Also upregulated was CD74 (invariant chain/Ii) which protects the MHCII peptide binding pocket from premature loading and several co-receptors (CD40, PDCD1LG2 and CD274) which modulate interactions between APCs and their corresponding T-cells.

#### MHCI system

The ingenuity pathways (Table 2) also include components of the MHCI system. We found that proteins involved in the endoplasmic-reticulum-associated complex that loads peptides on to antigen presenting proteins of the MHCI system, TAP1, TAP2 and TAPBP, increased >3 fold, with significant dose and time related changes (S1 Table, Table 1). We also found that expression of proteasome subunits PSMB8 and PSMB9 was increased more than 2-fold. These genes encode components of a modified proteasome, the immunoproteosome [1, 14], which generates peptides more suitable for presentation by the MHCI system.

#### Guanylate binding proteins

GBPs are a family of guanosine triphosphatases (GTPases) that are known to be induced by IFN-γ [15]. Because these proteins have a range of diverse immune supportive functions, we hypothesized that their upregulation could enhance the antimicrobial phenotype of neutrophils in patients treated with IFN-γ; and we examined our gene expression data, S1 Table, to see if transcription of any GBP genes were upregulated in this study. Large increases in the expression (2.6–13.1-fold) of various members of this family (GBPs 1,2,3,4,5,6,7) were observed. Upregulation of a GBP pseudogene (GBP1P1) that presumably retains the same transcriptional regulation as functional GBPs was also noted. All showed significant time and dose related changes (S1 Table and Table 2, Fig 4).

#### Innate immune receptors

Ingenuity pathway analysis (Table 2) identified “Role of Pattern Recognition Receptors in Recognition of Bacteria and Viruses” as a significant canonical pathway that was enhanced by the administration of IFN-γ. The upregulated genes in this pathway included some encoding innate immune receptors which bind to pathogen derived molecules and trigger anti-microbial, pro-inflammatory cellular changes. Expression of mRNA from the toll like, NOD like and c-type lectin families of innate immune receptors was altered with significant time and dose related effects (S1 Table, Table 1, Fig 4).

#### Fcγ receptors

Fcγ receptors mediate phagocytosis of antibody opsonized microbes directing uptake of pathogens into neutrophil phagolysosomes. Examination of our data revealed that transcript levels for two high affinity IgG receptors, FCGR1A and FCGR1B were upregulated 6.92- and 9.96-fold respectively following IFN-γ administration (Fig 4, S1 Table, and Table 1) and that the time and dose dependences were highly significant.

#### NADPH oxidase

Because NADPH oxidase activity was elevated transiently following IFN-γ administration (Fig 2) we examined the gene expression data to see if expression of any of the components of the NADPH oxidase followed a similar pattern. We found transient and significant spikes in expression of CYBB, which encodes the transmembrane oxidase component gp91^*phox*^, and NCF1, which encodes the oxidase component p47^*phox*^ (S1 Table, Table 1 and Fig 4). The pattern of expression of these genes compares quite well with the pattern of O_2_^-^ production in Fig 2D and 2E suggesting that their upregulation accounts for the increased NADPH oxidase activity. The decline in the initial peaks of O_2_^-^ generation and the appearance of a second peak at the 100μg/m^2^ dose may be explained by changes in gp91^*phox*^ expression; peak transcription of CYBB was reduced at the 100μg/m^2^ dose compared to 50μg/m^2^ and a slight second peak of CYBB expression occurred at 48 hours at 100μg/m^2^.

#### CXCR4

**Fig 5:**
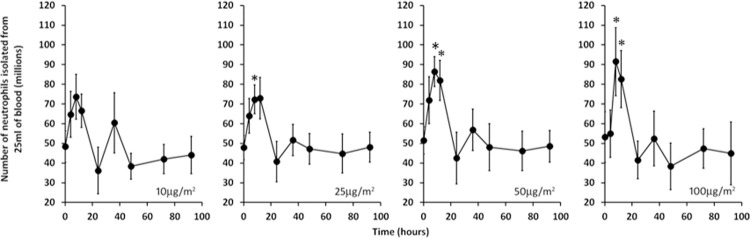
Elevated neutrophil yields are induced by IFN-γ. The indicated doses of IFN-γ were administered just after time 0 and the numbers of neutrophils isolated from 25ml of blood drawn at various times are shown. The error bars are SEM for n ≥7. The asterisks indicate differences (p = 0.0087–0.0479) from time 0 (two tailed t-test).

Expression of the CXC-chemokine receptor 4 (CXCR4) reduces the number of circulating PMNs [16, 17]. Our gene expression data showed that the levels of CXCR4 mRNA decreased by greater than 2-fold immediately after administration of IFN-γ, (S1 Table, Table 1 and Fig 4). While we did not have sequential blood counts to investigate the effect of decreases in CXCR4 expression, we were able to estimate whether IFN-γ administration was associated with a change in neutrophil numbers in peripheral blood by determining the yield of neutrophils isolated from equivalent amounts of blood obtained at each time point in the study. As can be seen in Fig 5, IFN-γ administration produced a transient rise in cell yield that increased in magnitude with increasing dose. Thus, the reduction in CXCR4 expression could provide at least a partial explanation for the corresponding spike in isolated neutrophil numbers.

#### GTP cyclohydrolase 1 (GCH1)

Strikingly, expression for GTPCH was upregulated 18-fold following IFN-γ administration with significant time and dose effects (S1 Table, Table 1, Fig 4). *GCH1* is the first and rate-limiting enzyme in tetrahydrobiopterin (BH4) biosynthesis. BH4 is an essential cofactor for NOS activity and the production of nitric oxide (NO). NO and reactive nitrogen intermediates (RNI) produced from it are microbicidal and have been shown to be an alternative to ROS mediated killing in neutrophils [18]. RNI are broad spectrum antimicrobial agents which can damage DNA, proteins and lipids in pathogens [19]. It seemed possible that an increase in RNI production by neutrophils might provide an alternative source of pathogen damaging molecules that could play a corporate or separate role with ROS. A review of expression for genes encoding the known nitric oxide synthase enzymes (NOS 1, 2 and 3) demonstrated these were not upregulated after administration of IFN-γ (data in S1 Table).

### NO production

**Fig 6:**
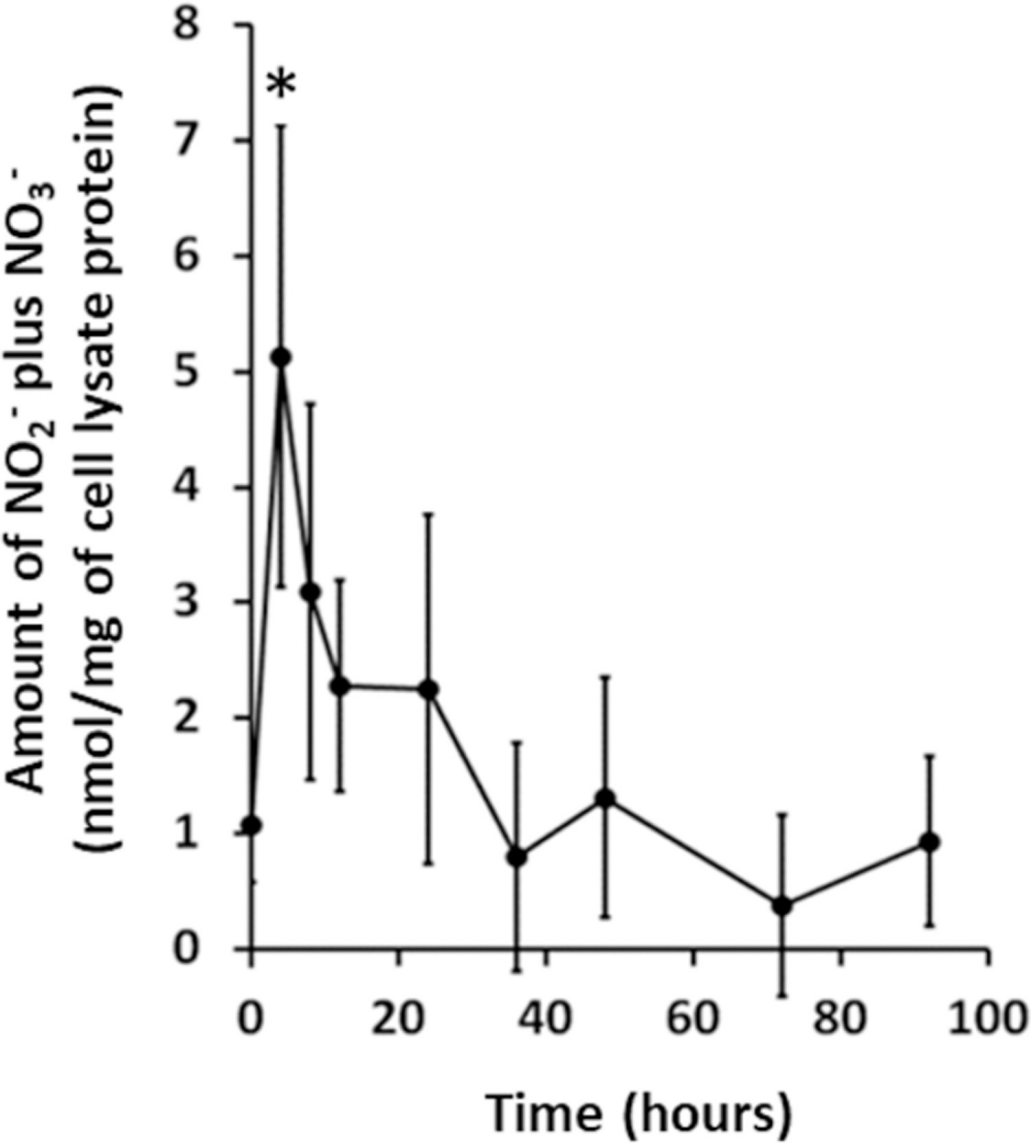
Nitric Oxide levels inferred from nitrate plus nitrite levels in neutrophil lysates. IFN-γ was administered to healthy volunteers just after blood was taken for the time 0 time point and cell lysates were prepared from isolated neutrophils at the indicated times. Nitrate plus nitrite levels were measured by a colorimetric assay. The data is presented as mean ± SEM for n≥6 samples. The asterisk indicates significant difference (p = 0.040) from time 0 (two-tailed t-test).

The increase in expression of mRNA for GTPCH prompted investigation into NO generation in neutrophil lysates isolated as part of the study. Cell lysates prepared from neutrophils isolated from subjects at the 100 μg/m^2^ dose were assayed for nitrate plus nitrite as a proxy for the unstable NO molecule. As shown in Fig 6, nitrite plus nitrate concentrations spiked early (4 hours, p = 0.040), were elevated 2-3-fold above baseline, and returned to baseline by 36 hours suggesting that NOS activity was increased after administration of IFN-γ.

**Fig 7:**
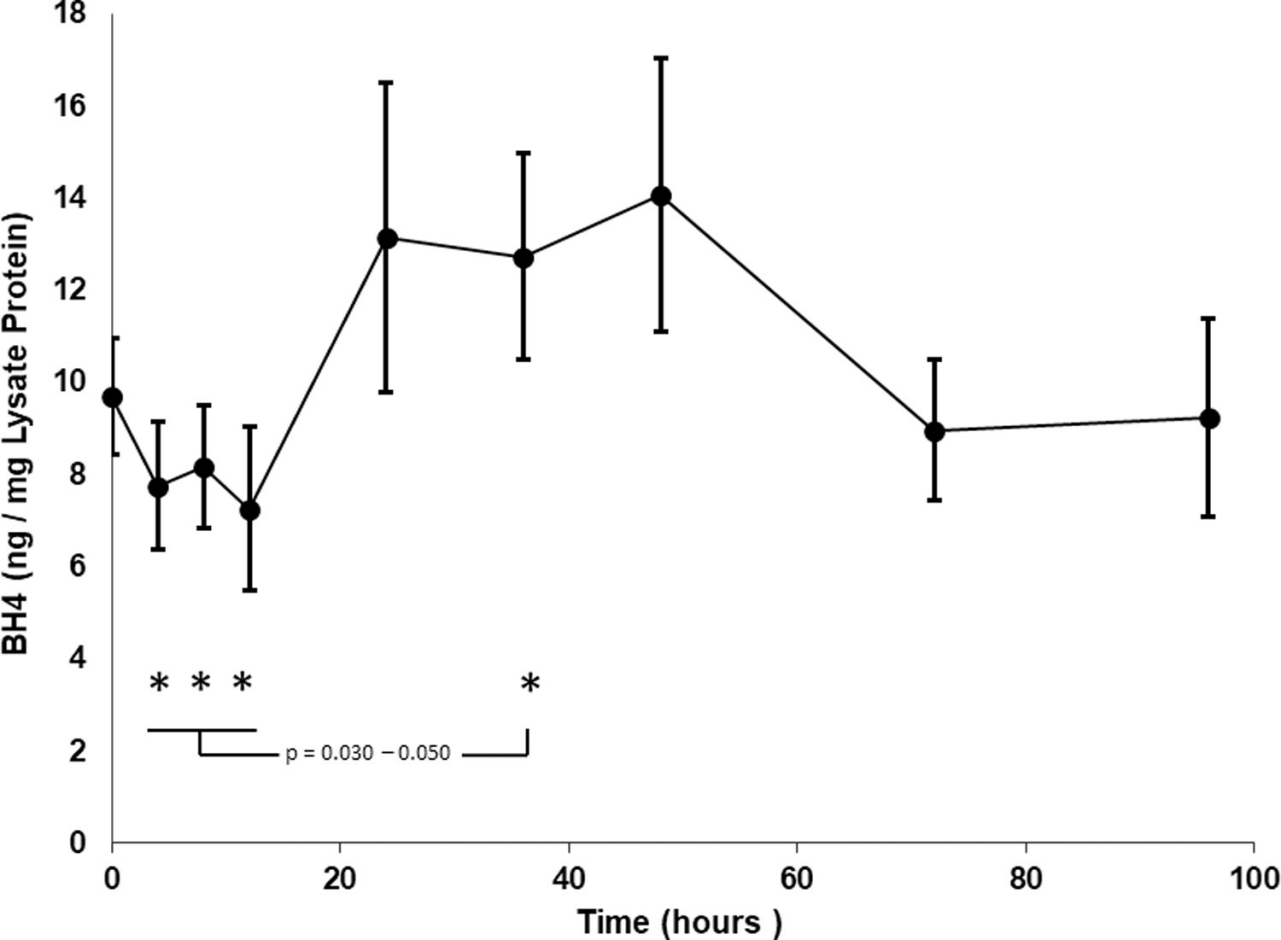
Tetahydrobiopterin (BH4) levels in neutrophil lysates. Free BH4 was measured in neutrophil lysates at all study timepoints by a competitive ELISA assay. Numbers represent mean ± SEM for 7 patients. No differences were between pre administration levels (time 0) and all other times. However, results for 4, 8, 12 hours compared to 36 hours (asterisks) were significantly different p = 0.030–0.050 by paired t-test.

Because BH4 is an essential cofactor required by NOS enzymes, increased GCH1 expression might lead to production of BH4, elevated NOS catalytic activity and thus enhanced NO production. Free BH4 levels were measured in neutrophil lysates from the 50 mcg/m^2^ dose (Fig 7). Because NOS has a high affinity for its cofactor, this assay will not measure BH4 bound to NOS. As determined by our technique, levels of BH4 dropped over the first 12 hours after administration, increased to a maximum at 36–48 hours by nearly 2-fold over the nadir (4, 8, 12 hours different from 36 hours, p = 0.030–0.050, paired t-test), returning to near baseline by 72–96 hours. The pattern is consistent with that expected for high NOS activity and NO production seen after IFN-γ administration. A similar pattern was seen at the 25 mcg/m^2^dose.

### Protein expression

**Fig 8:**
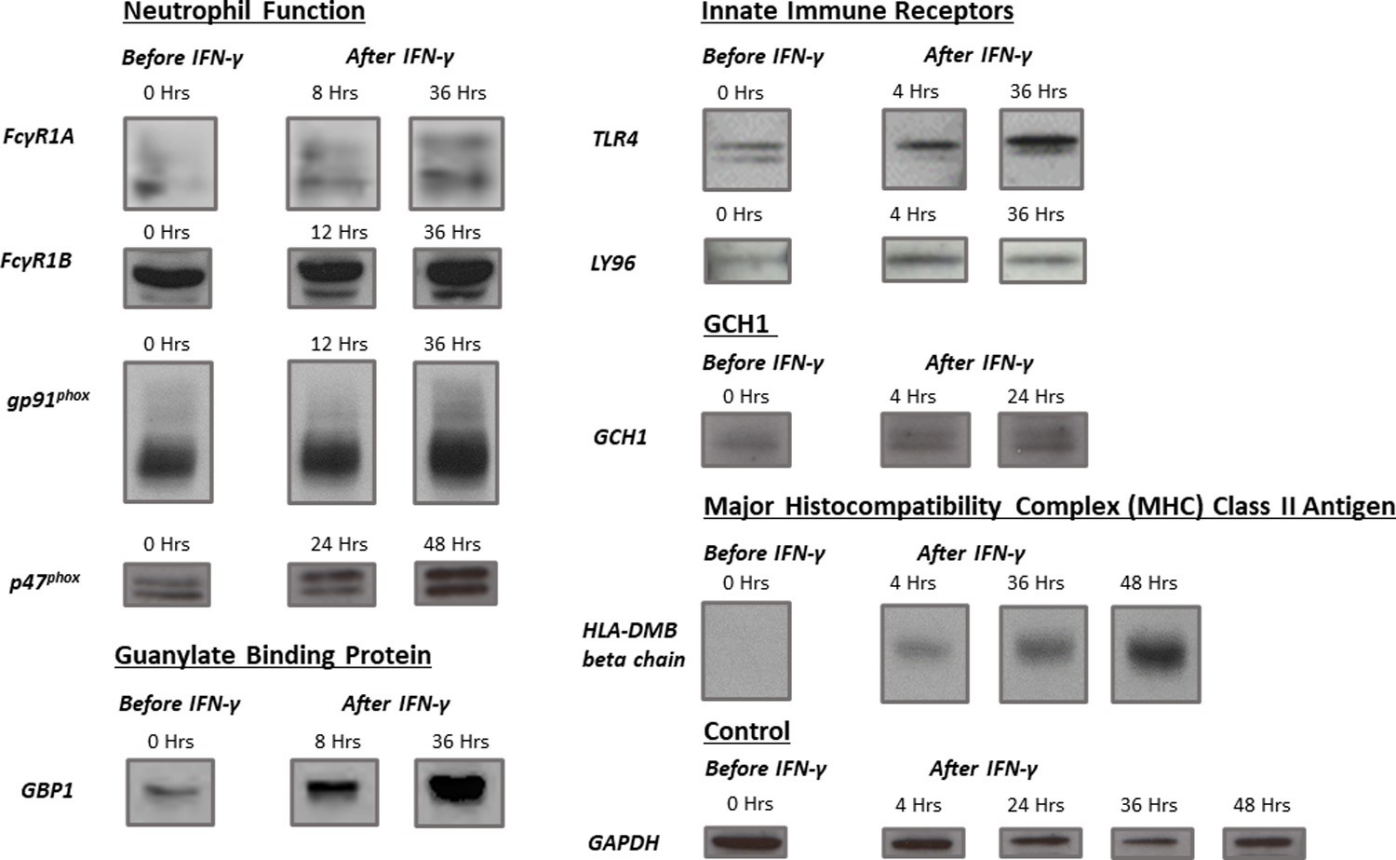
Western blot analysis for selected proteins. The levels of selected proteins for genes in Table 1 were investigated by Western blot. Lanes containing neutrophil lysate (50 μg protein) prior to administration of IFN-γ (time 0) on the left and samples from various times after administration are on the right. These are representative results from at least three separate subjects for each protein. The housekeeping enzyme glyceraldehyde 3-phosphate dehydrogenase (GAPDH) was included as a loading control.

To confirm that the observed changes in gene expression were reflected in neutrophil protein content, we performed Western blot analysis on lysates from subject samples before and after IFN-γ administration at the 25 or 50 mcg/m^2^ dose for selected proteins. Results are shown in Fig 8 and Table 3 which demonstrate increased protein expression for genes associated with neutrophil function (FCGR1A, FCGR1B, CYBB, and NCF1). Guanylate binding protein (GBP1), innate immune receptors (TLR4 and LY96), antigen presentation (HLA-DMB), and GTPCH. Minor early increases were demonstrated for *FcγRIA*, *FcγRIB*, and gp91^*phox*^ (8–12 hours) with significant peak levels at 36 hours (Table 3). The response for p47^*phox*^ was delayed in comparison with small early changes at 24 hours and the higher levels at 48 hours approaching significance. *GPB1* showed an increase at 8 hours and maximum and significant levels at 36 hours. For the remainder of the proteins, *TLR4*, *LY96*, *GCH1*, and *HLA-DM beta chain*, increased levels were seen very early after administration of the drug (4 hours) and significantly enhanced protein expression detected at 36, 36, 24, and 48 hours, respectively. The protein changes appeared consistent with gene expression. However, additional factors such as altered protein or mRNA stability, changes in translation, or the kinetics of PMN turnover in the vascular compartment might also affect the precise congruity. The changes in proteins shown are expected to be associated with alterations in PMN activity. This was the case in superoxide anion generation, and the pattern of *phox* protein levels could account for the changes noted in the PMN respiratory burst. However, extensive evaluation of PMN function was not determined in this study.

**Table 3:** Quantitation of proteins in neutrophil lysates*.

Protein	Ratio of Band Densities		
	**8 Hours**	**36 Hours**	
***FcγR1A***	1.93 ± 0.43	1.70 ± 0.12	
(n = 4)	*p = 0*.*117*	*p = 0*.*010*	
	**12 Hours**	**36 Hours**	
***FcγR1B***	1.37 ± 0.30	2.02 ± 0.20	
(n = 4)	*p = 0*.*312*	*p = 0*.*014*	
	**12 Hours**	**36 Hours**	
***gp91*** ^***phox***^	0.99 ± 0.13	1.56 ± 0.11	
(n = 3)	*p = 0*.*964*	*p = 0*.*035*	
	**24 Hours**	**48 Hours**	
***p47*** ^***phox***^	1.24 ± 0.08	1.87 ± 0.27	
(n = 3)	*p = 0*.*092*	*p = 0*.*083*	
	**8 Hours**	**36 Hours**	
***GBP1***	1.96 ± 0.35	3.21 ± 0.53	
(n = 4)	*p = 0*.*074*	*p = 0*.*025*	
	**4 Hours**	**36 Hours**	
***TLR4***	1.12 ± 0.14	1.95 ± 0.21	
(n = 3)	*p = 0*.*495*	*p = 0*.*047*	
	**4 Hours**	**36 Hours**	
***LY96***	1.48 ± 0.37	2.24 ± 0.37	
(n = 4)	*p = 0*.*289*	*p = 0*.*044*	
	**4 Hours**	**24 Hours**	
***GCH1***	2.14 ± 0.47	2.32 ± 0.27	
(n = 3)	*p = 0*.*137*	*p = 0*.*040*	
	**4 Hours**	**36 Hours**	**48 Hours**
***HLA-DMB***	0.96 ± 0.08	2.56 ± 0.60	2.32 ± 0.09
(n = 3)	*p = 0*.*653*	*p = 0*.*122*	*p = 0*.*005*

* Numbers represent ratio of band density at noted time after administration of IFN-γ compared to that before administration, presented as mean ± SEM with numbers of separate blots in parenthesis. p-values are for two-tailed, paired t-test.

## Discussion

We found that IFN-γ concentrations in plasma increased transiently after its administration, and the magnitude and persistence of the resulting peaks were dose dependent (Fig 2A). We also monitored plasma levels of IP-10, a small cytokine encoded by the gene CXCL10 whose expression in response to IFN-γ is well characterized [12]. The biological activity of the administered IFN-γ is demonstrated in Fig 2B which shows that plasma levels of IP-10 mirrored those of IFN-γ. The longer persistence of the IP-10 peaks presumably reflects its slower clearance relative to IFN-γ. IP-10 synthesis in response to IFN-γ has previously been shown to occur in monocytes, endothelial cells, and fibroblasts [12]. However, our gene expression data indicate that CXCL10 was dramatically upregulated in neutrophils, the maximal increase in CXCL10 observed in this study was 36.7-fold over baseline (S1 Table and Fig 4) suggesting the PMNs also contribute to increases in IP-10 in plasma. Another well-known IFN-γ response molecule is neopterin [13]. Its levels in plasma also increased in a dose dependent manner in response to IFN-γ; however, its response was delayed compared to IP-10 and its clearance was much slower as well (Fig 2C). Neopterin is synthesized from GTP by means of the biochemical pathway associated with GTP cyclohydrolase. We found that transcription of the gene GTPCH, which encodes GTP cyclohydrolase (*GCH1*), was upregulated dramatically in neutrophils after administration of IFN-γ (S1 Table, Table 1 and Fig 4). Since this enzyme catalyzes the first and rate limiting step in neopterin biosynthesis, these data suggest that neutrophils might account, at least partially, for the elevated plasma levels of neopterin observed in this study. A non-enzymatic oxidation step involved in neopterin formation could account for its delayed formation relative to IP-10.

A major aspect of this study was the detailed measurement of *in vivo* gene expression changes in isolated neutrophils following IFN-γ administration. As revealed by the gene expression changes listed in Table 1, and the canonical pathways in Table 2, we found variations in many genes including a number involved in classical as well as less appreciated aspects of neutrophil function. These broad alterations in gene expression indicate that IFN-γ induces extensive changes in neutrophil phenotype, and we propose that these could compensate, at least in part, in acquired or congenital neutrophil function defects.

One of the changes induced by IFN-γ was upregulated expression of numerous genes involved in antigen presentation via the MHCII system. Presentation of antigens by MHCII occurs on APCs, and neutrophils have not classically been thought to play a role in this process. However, several studies have shown that neutrophils can express MHCII molecules when treated with pro-inflammatory stimuli including IFN-γ [20–22]. The expressed MHCII molecules are functional since neutrophils have been shown to cause T-cell activation in response to staphylococcal enterotoxin, a superantigen which can be presented by MHCII without intracellular processing [21]. There is conflicting data related to whether neutrophils can fully process non-superantigens using the standard pathway; in one study, following MHCII induction, neutrophils treated with tetanus toxoid antigen were found to activate T-cells [21] but in another study processing of tetanus toxoid antigen was not seen [22]. Data showing that neutrophils express MHCII and acquire other dendritic cell-like properties in certain culture conditions and at inflammatory lesions also challenge the concept that neutrophils can never act as APCs or express MHCII components [23]. Our finding that many genes involved in the MHCII system are upregulated following IFN-γ administration supports the idea that neutrophils can acquire APC-like properties and indicates that antigen presentation by the MHCII system might be a major change in neutrophil phenotype induced by IFN-γ. We propose that IFN-γ might cause antigen presentation by the MHCII system in neutrophils thus enhancing an underappreciated interaction between innate and adaptive immunity.

In addition to the potential existence of the MHCII system in neutrophils, some data suggest that they might also express the MHCI system [24, 25]. Circulating neutrophils express MHCI molecules [26–28], and peptides added to neutrophils can be presented by MHCI molecules and can thus activate CD8 positive memory T cells [25]. We found that certain components of the MHCI system were upregulated in this study and thus, acquisition of antigen processing by the MHCI system might be another phenotypic change induced in neutrophils after IFN-γ treatment.

Upregulation of gene expression for a host of GBPs was also induced by IFN-γ treatment. Antimicrobial roles for many of these molecules have been described. GBP1 and 2 inhibit *Chlamydia trachomatis* in HeLa cells [29]. GBPs 1 and 6 are necessary for maximal inhibition of growth of *Listeria monocytogenes* and *Mycobacterium bovis* by RAW264.7 macrophages [30], and GBP1 was found to carry ubiquitinated-*p62/Sqstm1 protein* to autolysosomes where ubiquitin-derived bactericidal peptides were then generated. We propose that a similar mechanism might deliver bactericidal peptides to pathogen containing phagolysosomes in neutrophils developed under the influence of IFN-γ. In CGD patients treated with IFN-γ, increased delivery of bactericidal peptides to phagolysosomes might compensate for reduced ROS-mediated killing.

Increased transcription of a range of innate immune receptors was induced by IFN-γ treatment in this study (Tables 1 and 2). These molecules bind to pathogen-derived, pathogen-specific molecules triggering cellular changes involved in pathogen clearance. The upregulated receptors include several Toll-like receptors, *TLR5* which recognizes lipopolysaccharide (LPS), *LY96* which associates with *TLR4* to form the major LPS receptor, and *TLR8*. *TLR8* recognizes single stranded viral RNA [31] and is phylogenetically similar to *TLR7*, which can sense RNA species from bacteria such as group B *Streptococcus* [32]. Gene expression for the nod-like receptor, NOD1, which recognizes proteoglycan degradation products derived from bacteria [33] was upregulated more than 2 fold in this study as was that for CLEC4D, CLEC5A, CLEC6A and CLEC9A, members of the c-type lectin family of innate immune receptors. Thus, a further IFN-γ-mediated phenotypic change in neutrophils may reflect enhanced detection of, and responses to, pathogen derived molecular patterns.

The two high-affinity IgG receptor genes, (FCGR1A and FCGR1B) were upregulated by IFN-γ in this study (S1 Table, Table 1 and Fig 4). Given the role of Fcγ receptors in phagocytosis of IgG opsonized bacteria, their upregulation following administration of IFN-γ has clear implications for enhanced bactericidal activity.

We observed a transient, but large and dose-dependent increase in neutrophil yield following IFN-γ administration (Fig 5). Because *CXC-chemokine receptor 4* is a strong negative regulator of neutrophil levels in blood [16, 17], we examined the gene expression data in S1 Table and found that expression of its gene (CXCR4) spikes down after IFN-γ administration (Fig 4) thus providing at least a partial explanation for the corresponding increase in neutrophil yield. The role of CXCR4 downregulation in increasing neutrophil numbers is not the only effect related to this issue; cultured neutrophils were found to have reduced apoptosis in the presence of IFN-γ [34] which might also contribute to the peak in cell yield shown in our study. An attractive hypothesis for the effect of IFN-γ on neutrophil phenotype may also relate to cell numbers. It is possible that the changes seen are related solely to the mobilization of cells from a separate pool. This seems less likely because of the extent of activity exhibited with *in vivo* administration of IFN-γ and the extensive gene expression, functional and biochemical changes documented. We have shown that maturing and matured cells respond to IFN-γ [5, 9]. Mobilization, in part, may play a role along with transcriptional activation and gene expression in the changes induced in peripheral blood neutrophils by IFN-γ.

IFN-γ caused transient increases in the expression of CYBB and NCF1, which encode the gp91^*phox*^ and p47^*phox*^, components of the NADPH oxidase, thus providing a clear explanation of the enhanced O_2_^-^ generation seen in response to PMA and fMLF (Fig 2D and 2E). Clearly upregulation of NADPH oxidase components is of no significance in CGD patients where the disease mutation completely prevents the formation of a functional NADPH oxidase. However, it may help in cases of variant CGD where expression of a less stable and therefore less abundant oxidase protein occurs. In this case enhanced expression of CYBB alleles that encode unstable proteins could boost suboptimal levels of these proteins allowing residual oxidase activity, an issue which has been shown to make a difference in long term survival in these patients [35].

GCH1 was also upregulated following IFN-γ administration. This protein is the first and rate-limiting enzyme in biosynthesis of tetrahydrobiopterin (BH4) an essential cofactor required by nitric oxide synthase (NOS) enzymes. This presented an attractive hypothesis that IFN-γ might increase NOS enzyme activity by stimulating formation of BH4 in neutrophils. Our method for measuring the NOS cofactor by competitive ELISA detected free BH4 and was not likely to show elevated levels during peak NOS activity when BH4 would be bound to NOS. Although no statistically significant changes in BH4 levels before administration of IFN-γ were documented compared to results at later times, significant decreases were seen at 4, 8, and 12 hours after administration of the drug compared to increases observed at 36 hours. In fact, the timing of decreasing cofactor levels coincided exactly with the maximum NOS activity measured in the neutrophil lysates with increases in BH4 after the enzyme activity waned. The details of our specific assay, issues with retrospective analysis of samples, large variations in study subject results, contributions to neutrophil BH4 from other cells and tissues in an *in vivo* study and the specific biochemical pathways in neutrophils may impact the results shown here and further investigation of the biopterin pathway is required. Most importantly, however, generation of NO, measured as nitrite/nitrate, was demonstrated to peak by 4 hours after IFN-γ administration Because NO, and RNI produced from it, are broad spectrum microbicidal agents and have been shown to be an alternative to ROS mediated killing in neutrophils [18, 19], we suggest that IFN-γ mediated increases in RNI could provide a species of reactive molecules to enhance the microbicidal activity of the neutrophil.

Finally, gene expression changes were correlated with protein content by Western blot analysis for selected proteins. Analysis included proteins for gp91^*phox*^, p47^*phox*^, *FcγR1A*, *FcγR1B*, *TLR4*, *LY96*, *GBP1*, *HLA-DM beta chain*, and *GCH1*. Protein levels were increased after IFN-γ administration providing biochemical correlation with gene expression. Alterations in gene and protein expression are expected to be associated with changes in cell function. This was the case in superoxide anion generation, but other neutrophil functions were not determined in this study and remain to be defined.

The results of this study demonstrate that administration of IFN-γ induces dramatic *in vivo* changes in circulating neutrophils. The alterations in gene expression, protein expression and function shown here are similar to those demonstrated for IFN-γ in an *in vitro* model of myeloid maturation in a myeloid promyelocytic leukemia cell line [5, 9] and imply that the effects of this cytokine on neutrophils include enhancements in classically recognized neutrophil activity (i.e. NADPH oxidase activity, Fc receptor mediated ingestion and pathogen recognition by innate immune receptors) as well as more novel functional changes (i.e. interaction with the adaptive immune system via MHCI and MHCII, activation of GBPs, upregulation of NO production and enhancement of neutrophil release into the circulation). Our findings suggest that there may not be a single process by which IFN-γ enhances the neutrophil functional capacity, but rather several mechanisms. With effects on both classical and less appreciated immune neutrophil functions, IFN-γ may induce a more potent cell participating in innate immune host defense, one that can help circumvent clinical complications resulting from neutrophil dysfunction. These findings are important for understanding how IFN- γ is beneficial in CGD and how the expanded use of this drug may support other disorders in which the neutrophil or innate immune response is compromised.

## Supporting information

S1 Checklist (PDF)

S1 Table Genes which showed changes in expression after administration IFN-γ over time or dose. (XLSX)

S1 Raw images Western blot raw images for Fig 8. (PDF)

S1 Protocol (PDF)

## References

[1] Pollard KM, Cauvi DM, Toomey CB, Morris KV, Kono DH. Interferon-gamma and systemic autoimmunity. *Discov Med* 2013;16:123–131. 23998448 PMC3934799

[2] Schoenborn JR, Wilson CB. Regulation of interferon-gamma during innate and adaptive immune responses. *Adv Immunol* 2007;96:41–101. doi: 10.1016/S0065-2776(07)96002-2 17981204

[3] Green DS, Young HA, Valencia JC. Current prospects of type II interferon gamma signaling and autoimmunity. *J Biol Chem* 2017;292:13925–13933. doi: 10.1074/jbc.R116.774745 28652404 PMC5572907

[4] Ellis TN and Beaman BL. Interferon-γ activation of polymorphonuclear neutrophil function. Immunology 2004;112:2–12. doi: 10.1111/j.1365-2567.2004.01849.x 15096178 PMC1782470

[5] Ellison MA, Gearheart CM, Porter CC, Ambruso DR. IFN-gamma alters the expression of diverse immunity related genes in a cell culture model designed to represent maturing neutrophils. PLoS.One. 2017;12:e0185956. doi: 10.1371/journal.pone.0185956 28982143 PMC5628906

[6] Ambruso DR, Johnston RBJ. Chronic Granulomatous Disease and Common Variable Immunodeficiency Disorders. In: Wilmott R.W., Boat T.F., Bush A., Chernick V., Deterding R, Ratjen F., eds. Kendig and Chernick’s Disorders of the Respiratory Tract in Children.: WB Saunders Co; 2012.

[7] A controlled trial of interferon gamma to prevent infection in chronic granulomatous disease. The International Chronic Granulomatous Disease Cooperative Study Group. N.Engl.J.Med. 1991;324:509–516. doi: 10.1056/NEJM199102213240801 1846940

[8] Marciano BE, Wesley R, De Carlo ES et al. Long-term interferon-gamma therapy for patients with chronic granulomatous disease. Clin.Infect.Dis. 2004;39:692–699. doi: 10.1086/422993 15356785

[9] Ellison MA, Thurman G, Gearheart CM et al. INF-gamma Enhances Nox2 Activity by Upregulating phox Proteins When Applied to Differentiating PLB-985 Cells but Does Not Induce Nox2 Activity by Itself. PLoS.One. 2015;10:e0136766. doi: 10.1371/journal.pone.0136766 26317224 PMC4552644

[10] Leavey PJ, Sellins KS, Thurman G et al. In vivo treatment with granulocyte colony-stimulating factor results in divergent effects on neutrophil functions measured in vitro. Blood 1998;92:4366–4374. 9834243

[11] Ambruso DR, Knall C, Abell AN et al. Human neutrophil immunodeficiency syndrome is associated with an inhibitory Rac2 mutation. Proc.Natl.Acad.Sci.U.S.A 2000;97:4654–4659. doi: 10.1073/pnas.080074897 10758162 PMC18288

[12] Luster AD, Unkeless JC, Ravetch JV. Gamma-interferon transcriptionally regulates an early-response gene containing homology to platelet proteins. Nature 1985;315:672–676. doi: 10.1038/315672a0 3925348

[13] Fuchs D, Weiss G, Reibnegger G, Wachter H. The role of neopterin as a monitor of cellular immune activation in transplantation, inflammatory, infectious, and malignant diseases. Crit Rev.Clin.Lab Sci. 1992;29:307–341. doi: 10.3109/10408369209114604 1489521

[14] Wang J, Maldonado MA. The ubiquitin-proteasome system and its role in inflammatory and autoimmune diseases. Cell Mol.Immunol. 2006;3:255–261. 16978533

[15] Martens S, Howard J. The interferon-inducible GTPases. Annu.Rev.Cell Dev.Biol. 2006;22:559–589. doi: 10.1146/annurev.cellbio.22.010305.104619 16824009

[16] Eash KJ, Means JM, White DW, Link DC. CXCR4 is a key regulator of neutrophil release from the bone marrow under basal and stress granulopoiesis conditions. Blood 2009;113:4711–4719. doi: 10.1182/blood-2008-09-177287 19264920 PMC2680371

[17] Summers C, Rankin SM, Condliffe AM et al. Neutrophil kinetics in health and disease. Trends Immunol. 2010;31:318–324. doi: 10.1016/j.it.2010.05.006 20620114 PMC2930213

[18] Malawista SE, Montgomery RR, van BG. Evidence for reactive nitrogen intermediates in killing of staphylococci by human neutrophil cytoplasts. A new microbicidal pathway for polymorphonuclear leukocytes. J.Clin.Invest 1992;90:631–636. doi: 10.1172/JCI115903 1379614 PMC443143

[19] Schairer DO, Chouake JS, Nosanchuk JD, Friedman AJ. The potential of nitric oxide releasing therapies as antimicrobial agents. Virulence. 2012;3:271–279. doi: 10.4161/viru.20328 22546899 PMC3442839

[20] Radsak M, Iking-Konert C, Stegmaier S, Andrassy K, Hansch GM. Polymorphonuclear neutrophils as accessory cells for T-cell activation: major histocompatibility complex class II restricted antigen-dependent induction of T-cell proliferation. Immunology 2000;101:521–530. doi: 10.1046/j.1365-2567.2000.00140.x 11122456 PMC2327116

[21] Fanger NA, Liu C, Guyre PM et al. Activation of human T cells by major histocompatability complex class II expressing neutrophils: proliferation in the presence of superantigen, but not tetanus toxoid. Blood 1997;89:4128–4135. 9166855

[22] Gosselin EJ, Wardwell K, Rigby WF, Guyre PM. Induction of MHC class II on human polymorphonuclear neutrophils by granulocyte/macrophage colony-stimulating factor, IFN-gamma, and IL-3. J.Immunol. 1993;151:1482–1490. 8335942

[23] Takashima A, Yao Y. Neutrophil plasticity: acquisition of phenotype and functionality of antigen-presenting cell. J.Leukoc.Biol. 2015;98:489–496. doi: 10.1189/jlb.1MR1014-502R 25632045

[24] Potter NS, Harding CV. Neutrophils process exogenous bacteria via an alternate class I MHC processing pathway for presentation of peptides to T lymphocytes. J.Immunol. 2001;167:2538–2546. doi: 10.4049/jimmunol.167.5.2538 11509593

[25] Reali E, Guerrini R, Moretti S et al. Polymorphonuclear neutrophils pulsed with synthetic peptides efficiently activate memory cytotoxic T lymphocytes. J.Leukoc.Biol. 1996;60:207–213. doi: 10.1002/jlb.60.2.207 8773582

[26] Jack RM, Fearon DT. Selective synthesis of mRNA and proteins by human peripheral blood neutrophils. J.Immunol. 1988;140:4286–4293. 2453576

[27] Neuman E, Huleatt JW, Vargas H, Rupp EE, Jack RM. Regulation of MHC class I synthesis and expression by human neutrophils. J.Immunol. 1992;148:3520–3527. 1534098

[28] Lloyd AR, Oppenheim JJ. Poly’s lament: the neglected role of the polymorphonuclear neutrophil in the afferent limb of the immune response. Immunol.Today 1992;13:169–172. doi: 10.1016/0167-5699(92)90121-M 1642755

[29] Tietzel I, El-Haibi C, Carabeo RA. Human guanylate binding proteins potentiate the anti-chlamydia effects of interferon-gamma. PLoS.One. 2009;4:e6499. doi: 10.1371/journal.pone.0006499 19652711 PMC2714978

[30] Kim BH, Shenoy AR, Kumar P et al. A family of IFN-gamma-inducible 65-kD GTPases protects against bacterial infection. Science 2011;332:717–721. doi: 10.1126/science.1201711 21551061

[31] Akira S, Uematsu S, Takeuchi O. Pathogen recognition and innate immunity. Cell 2006;124:783–801. doi: 10.1016/j.cell.2006.02.015 16497588

[32] Mancuso G, Gambuzza M, Midiri A et al. Bacterial recognition by TLR7 in the lysosomes of conventional dendritic cells. Nat.Immunol. 2009;10:587–594. doi: 10.1038/ni.1733 19430477

[33] Kanneganti TD, Lamkanfi M, Nunez G. Intracellular NOD-like receptors in host defense and disease. Immunity. 2007;27:549–559. doi: 10.1016/j.immuni.2007.10.002 17967410

[34] Colotta F, Re F, Polentarutti N, Sozzani S, Mantovani A. Modulation of granulocyte survival and programmed cell death by cytokines and bacterial products. Blood 1992;80:2012–2020. 1382715

[35] Kuhns DB, Alvord WG, Heller T, Field JJ, Pike KM, Marciano BE Uzel E, DeRaven SS, Priel DA, Soule BPZarember KA, Malech HL. Holland SM, Gallin JI. Residual NADPH oxidase and survival in chronic granulomatous disease. N Engl J Med. 2010; 363:2600–2610. doi: 10.1056/NEJMoa1007097 21190454 PMC3069846

