# Supplementary figures and images for: In vivo interferon-gamma induced changes in gene expression dramatically alter neutrophil phenotype
Source: PLoS One. 2022 Feb 3;17(2):e0263370. doi: 10.1371/journal.pone.0263370 (PMC8812922; doi:10.1371/journal.pone.0263370)

**Supplementary Information (S2).** Western blot raw images for Figure 8.

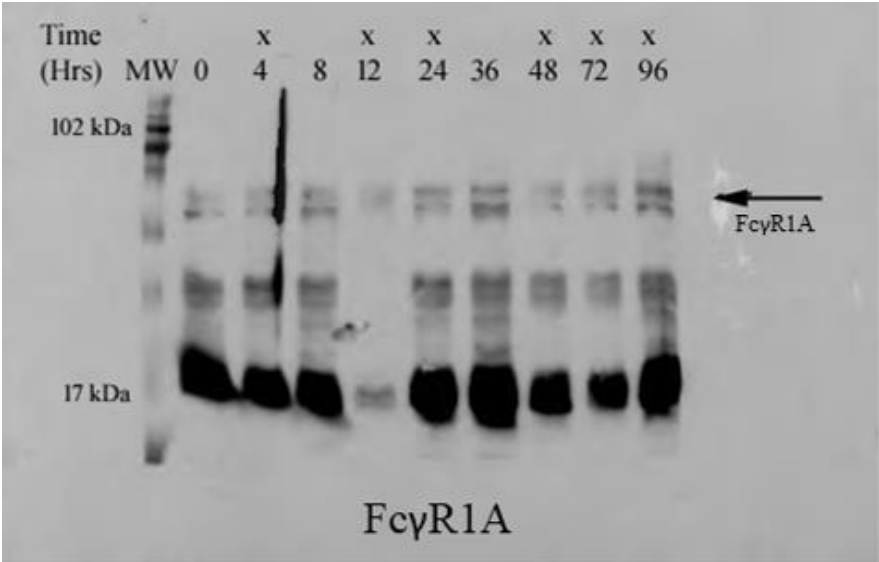

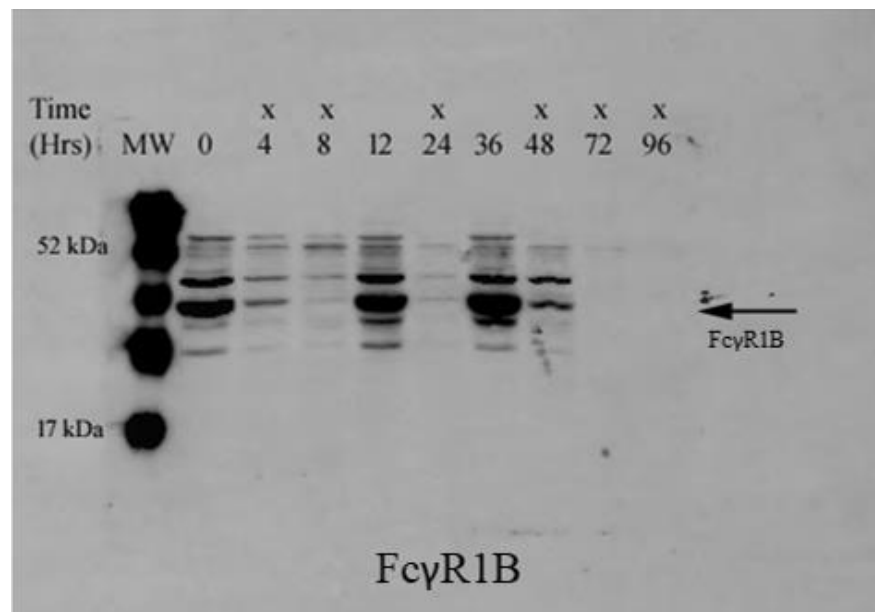

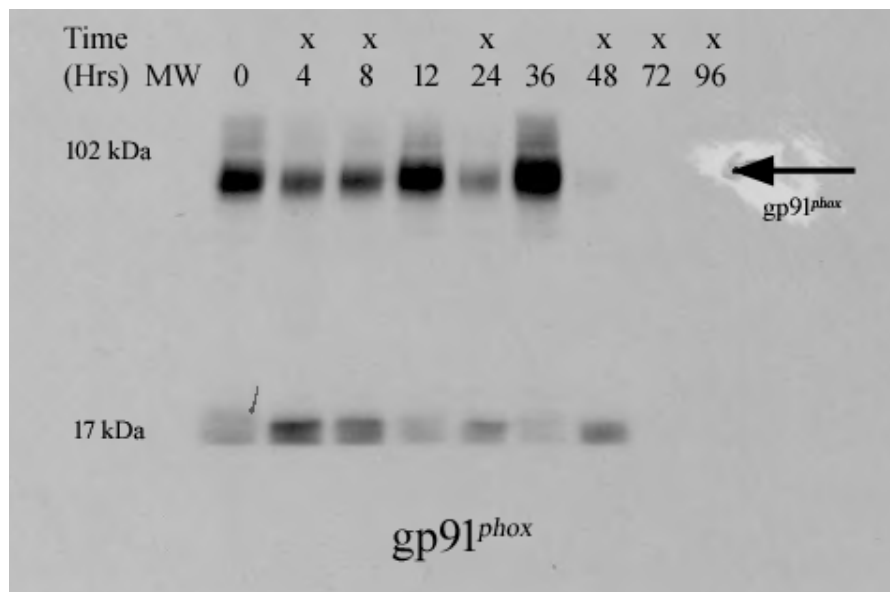

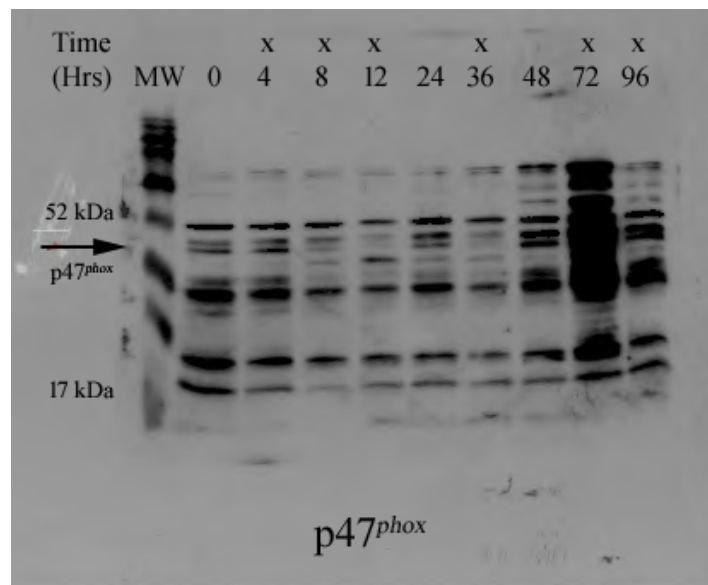

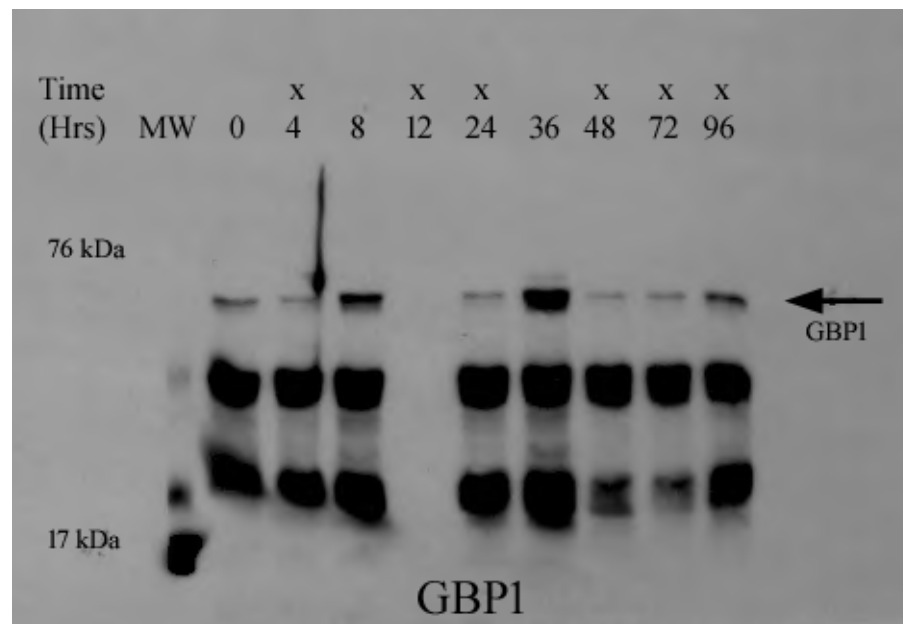

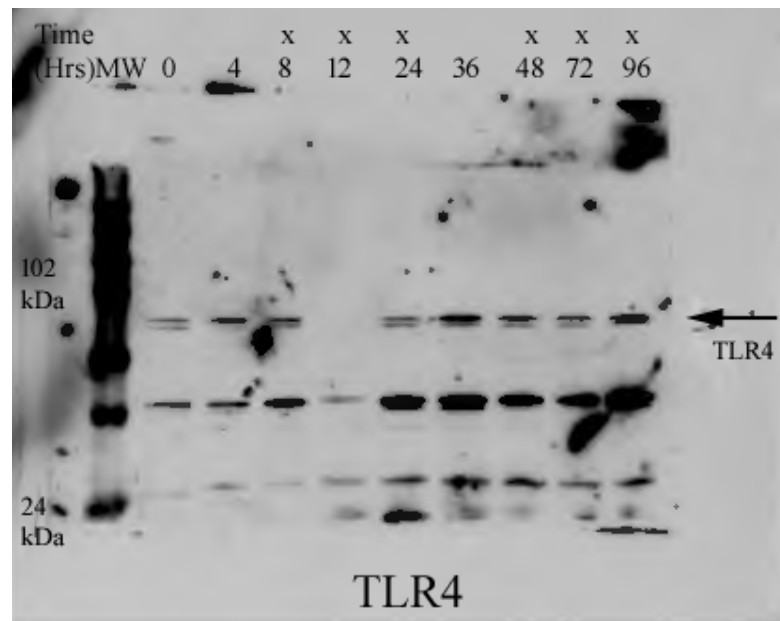

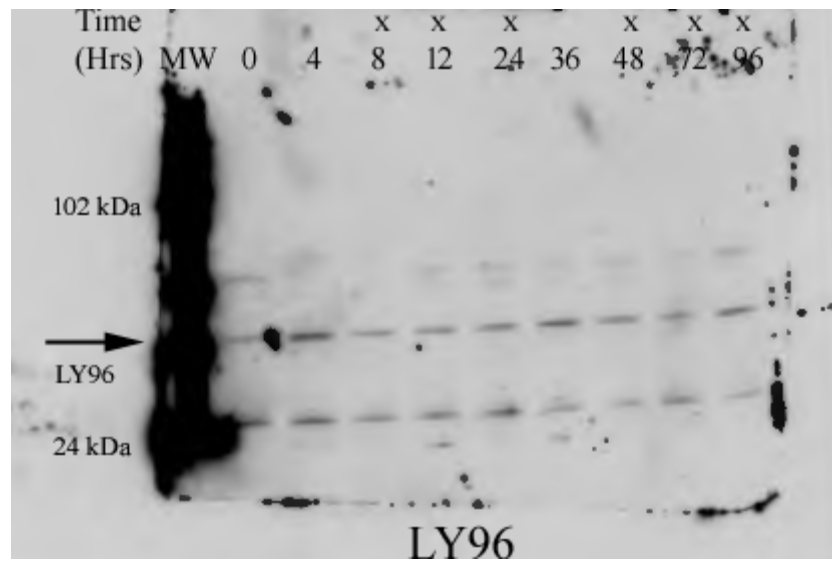

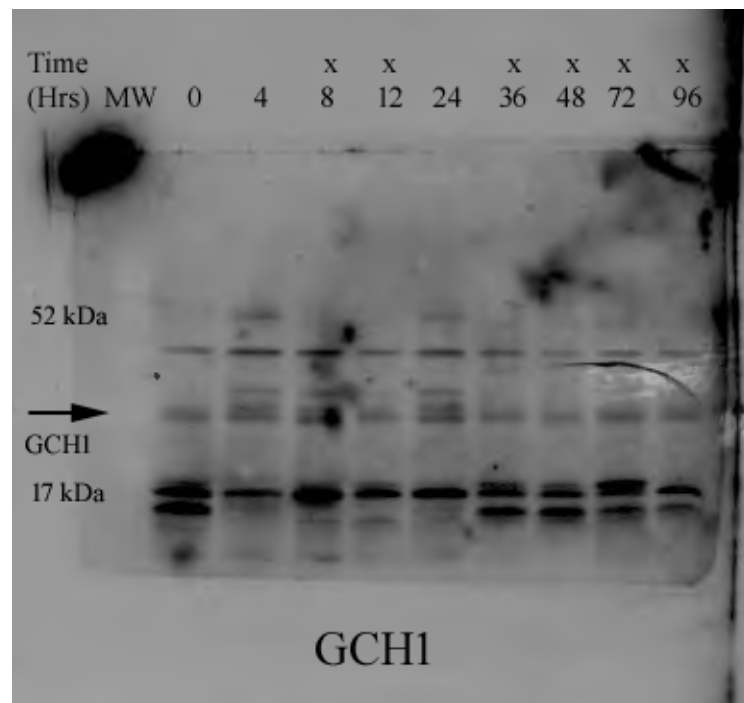

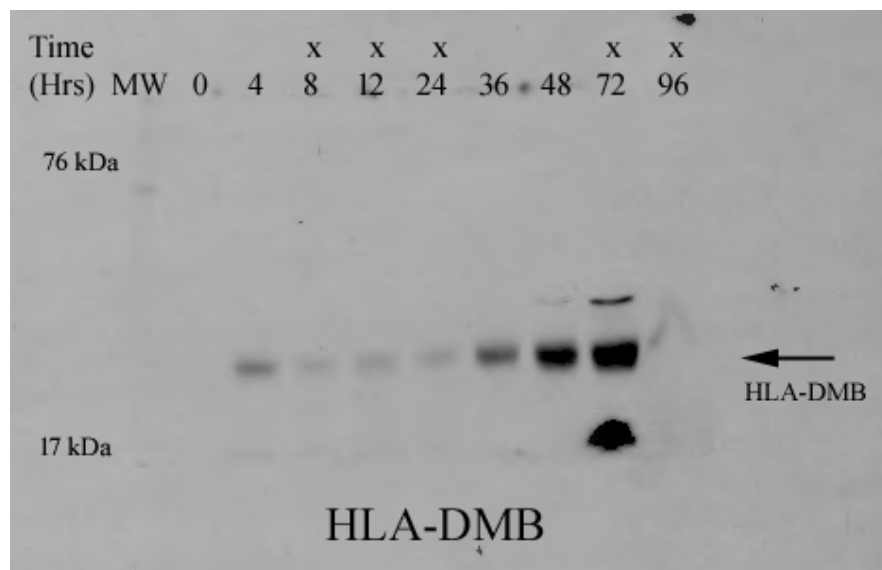

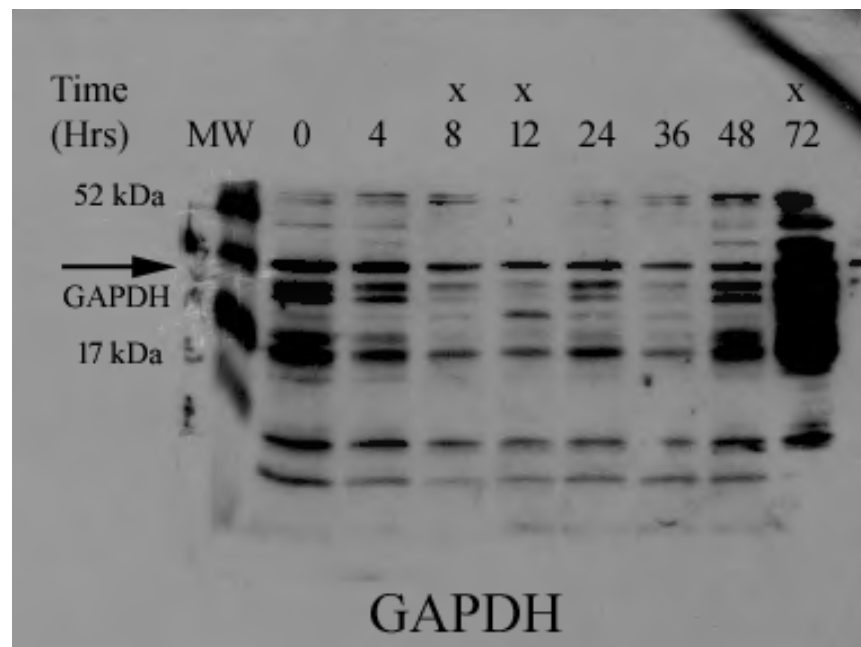

Supplement: S1 Raw images — (PDF) [file pone.0263370.s003.pdf]
