# Supplementary material for: In vivo interferon-gamma induced changes in gene expression dramatically alter neutrophil phenotype
Source: PLoS One. 2022 Feb 3;17(2):e0263370. doi: 10.1371/journal.pone.0263370 (PMC8812922; doi:10.1371/journal.pone.0263370)
Supplement: S1 Protocol — (PDF) [file pone.0263370.s004.pdf]

COMIRB Protocol

COLORADO MULTIPLE INSTITUTIONAL REVIEW BOARD

CAMPUS BOX F-490 TELEPHONE: 303-724-1055 Fax: 303-724-0990

Protocol #: 15-1643

Protocol Title: **Effect of Interferon-gamma 1-b (IFN-γ Ib) on Innate Immune Cells**

Protocol Version Date: October 12, 2015 (Amendment 2)

Principal Investigator: Daniel R. Ambruso, MD
Professor, University of Colorado School of Medicine

Immunohematology Laboratory:
Department of Pediatrics, University of Colorado Anschutz Medical Campus
Children's Hospital Colorado
Dan Ambruso Laboratory
13123 East 16th Avenue
Room A0941
Aurora, CO 80045
Phone: 303-324-2171 (Dr. Ambruso's Cell)
Lab Phone: 720-777-8057
Email: Daniel.amburso@ucdenver.edu

Funding Institution: Horizon Pharma Ireland Ltd.

Table of Contents

1. **Objectives** 4
Hypothesis and Specific Aims 4
Primary Objective 4
Secondary Objective 4

2. **Background** 4
Preliminary Studies 5
Rationale for selected approach and trial design 6

3. **Participation Selection** 7
Inclusion criteria 7
Exclusion criteria 7
Enrollment 8
Registration Process 8

4. **Treatment Plan** 8
Study Schema (see next page): 8
Single Dose (SD) Cohort 10
Sample Size 10
Laboratory Studies: 11
Statistical Analysis: See Section below, Statistical Considerations 14
Steady State (SS) Cohort 14
Sample Size: 14
Laboratory Studies: 15
Statistical Analysis: See section below, Statistical Considerations. 17

5. **Specimen Collections, Labeling and Storage** 18

6. **Duration of Study** 18

7. **Participant Stopping Criteria** 18

8. **DRUG INFORMATION FOR IFN-γ lb (Acctimmune®)** 18
Dosage: 18
Adjustment of Dosing: 18
Availability: 19
Accountability: 19
Source and Pharmacology: 19
Toxicity: 19
Formulation and Stability: 20

Drug Preparation: 20
9. Protocol Workflow 20
10. Experimental Methods 20
11. Operational and Statistical Considerations 21
Sample size justifications 21
12. Adverse Events related to IFN-γ lb 21
Supportive Care Guidelines for expected AEs: 22
13. Adverse Event Reporting Requirements 22
Adverse Events Definitions 23
Expedited Adverse Event Reporting to the FDA 24
14. Regulatory Requirements 24
Informed Consent 24
Study Documentation 24
15. Data and Safety Monitoring 25
Data Safety and Monitoring Plan 25
16. Statistical Analysis Plan 25
17. References 27
18. Reimbursement of Study Subjects 27
19. Materials Provided by Horizon Pharma Ireland Ltd. 27
20. APPENDIX I 28
21. Appendix II: Toxicity Grading Scale for Healthy Adult and Adolescent Volunteers Enrolled in Preventative Vaccine Clinical Trials 32
22. Appendix III: Justification for proposed ACTIMMUNE® (Interferon gamma-1b) dosing in: COMIRB #14-2231: Effect of IFN- γ-1b on Innate Immune cells 37
23. Appendix IV: Enrollment Timeline for both Single Dose (SD) and Steady State (SS) Cohorts: 50

Objectives

Hypothesis and Specific Aims

We hypothesize that neutrophils and monocytes developed under the influence of Interferon- gamma-1b (IFN-γ-1b, Actimmune*) *in vivo* will display enhanced function across a broad range of activities related in large part to the transcriptional activation effects of this cytokine. We will evaluate the effects of IFN-γ in healthy human subjects *in vivo* on gene expression, biologic activity markers, and functional activity of myeloid cells in single dose studies and in steady state studies.

Primary Objective

To better define in vivo timing of biological effects of IFN-γ 1b on monocyte and neutrophil cells from healthy subjects.

Secondary Objective

To characterize the chronic effects of IFN-γ 1b on function and biochemistry of neutrophils and monocytes developed under the influence of this cytokine in healthy human subjects.

Background

Named for their potent ability to interfere and protect against viral infections, interferons (IFNs) have many regulatory effects on the immune system.[1] Of the members of the two classes of these compounds, IFN-γ has the most diverse and powerful immune effects. Studies have mostly evaluated IFN-γ interactions with cells of adaptive immunity, including macrophages and lymphocytes. Effects on innate immunity, particularly polymorphonuclear leukocytes or neutrophils and monocytes are less well studied. However, investigations have suggested that IFN-γ may be involved in signal transduction, gene expression, the respiratory burst and neutrophil NADPH oxidase (Nox2) activity, phagocytosis, motility, microbicidal activity, and apoptosis. Not all of these functions are enhanced by IFN-γ; but the clinical use of this cytokine has been driven, in part by these results. For example, the primary motivation for initiating investigation of its beneficial clinical effects in Chronic Granulomatous Disease (CGD) was its effects on Nox2 activity.[2] Most data in this area was based on studies using differentiated neutrophils from peripheral blood.[1] However, the phenotype of neutrophils developed under the influence of this cytokine, not just changes expressed by exposure of differentiated cells to IFN-γ, is critical to understanding the physiologic effects of IFN-γ and the broad applications for its use in treatment of a range of human diseases. To expand our understanding of the role of IFN-γ in the development and functional integrity of the neutrophil, we have completed a series of studies with PLB-985 cells in an *in vitro* culture system of myeloid cells. In this proposal, we will evaluate innate immune activation and phagocyte function in healthy adult volunteers who are receiving IFN-γ.

---

*Actimmune is a registered trademark of Horizon Pharma, Inc.

Preliminary Studies

In our initial studies, we investigated the effects of IFN-γ on a promyelocytic cell line, PLB-985cells that can be differentiated into neutrophil-like cells with addition of DMSO to the culture. PLB-985 cells were grown under standard culture conditions (5% CO_2, 37°C) with RPMI and 10% bovine serum and were matured with the addition of 1.3% DMSO in the presence and absence of various concentrations of IFN-γ-1b (Horizon Pharma Ireland Ltd.). After 72 and 96 hours, the cells exhibited neutrophil-like phenotype. As controls, cells were incubated with media or media plus IFN-γ alone.

Superoxide anion (O_2^-) production was measured as SOD inhabitable cytochrome c reduction after exposure to PMA (200 ng/mL) or FMLP (1 µM). Cell lysates were prepared in the presence of 0.1% SDS, 1% Triton X 100 and a protease inhibitor mix. Proteins from the lysate were separated on 10-15% SDS PAGE, blotted onto PVDF or nitrocellulose, exposed to primary antibodies for phox proteins overnight at 4°C and then appropriate peroxidase labeled secondary antibodies. Protein/antibody complexes were detected with an ECL detection system and were quantitated by densitometry with Image J software.

Figure 1 (Appendix) shows results for PLB-985 cells cultured for 4 days with 30ng/ml (600 IU/ml) IFN-γ, DMSO or DMSO+ IFN-γ and subsequently stimulated with PMA or fMLF with O_2^- production was measured over 5 minutes (for fMLF) or 10 minutes (for PMA). IFN-γ alone resulted in minimal O_2^- generation. For fMLF a significant ($p<0.05$) increase in O_2^- was seen when DMSO was included in the culture and for PMA a significant ($p<0.005$) increase was seen. For both agonists an additional increase ($p<0.005$) was documented when IFN-γ was included with DMSO during the culture, fMLF and PMA.

When a range of IFN-γ concentrations (5-60 ng/ml) were co-applied during DMSO mediated PLB-985 differentiation over 3 days, the increase in O_2^- production was concentration dependent and reached a plateau at 30ng/ml for both stimuli (see Figure 2, Appendix).

Quantitation of phox proteins completed by Western blot is shown in Figure 3, Appendix. Culture of cells with IFN-γ alone resulted in dramatic increases in p22phox and gp91phox but failed to cause more than minimal increases in the initially low levels of p40phox, p47phox and p67phox accounting for the inability of IFN-γ alone to induce oxidase activity. Differentiation of the cells with DMSO caused a small decrease in p22phox and a small increase in gp91phox but also resulted in large increases in p40phox, p47phox and p67phox, which would explain the ability of DMSO to induce Nox2 activity. Inclusion of IFN-γ with DMSO further increased p47phox, and increased p22phox and gp91phox relative to DMSO alone, potentially explaining the enhanced Nox2 activity noted above.

In striking contrast, when IFN-γ was applied to cells first differentiated with DMSO, a minimal increase in fMLF induced O_2^- production was seen and only a small increase in PMA induced O_2^- generation was measured (Figure 2, Appendix). Consistent with this, IFN-γ applied to pre-differentiated PLB-985 cells did not enhance p40phox, and gp91phox, minimally increased p22phox, and decreased p47phox and p67phox slightly (Figure 4, Appendix). Further studies are currently underway to evaluate phagocytosis and bactericidal activity in this cell culture system.

These studies demonstrate that IFN-γ induces very different phenotypes in myeloid cells depending on the differentiation level of the cell. Dramatic differences were noted whether the IFN-γ was present during maturation of these myeloid cells as opposed to after differentiation and include major differences in expression of phox proteins as well as activity of the Nox2 enzyme system. These data not only support the administration of IFN-γ for specific neutrophil dysfunction disorders, but also suggest, in theory, expanded uses of this cytokine.

Additional investigations were completed to evaluate cell motility in neutrophils developed under the Influence of IFN-γ in the PLB-985 cell culture system. PLB-985 cells were cultured under the conditions described above. Chemotaxis was completed as previously described (J Immunol 2006; 176: 7621-7627). Cells were pre-incubated with calcium-acetoxymethylester at 37°C for 30 minutes in the dark with agitation. Cell migration was monitored using a 96 well ChemoTx disposable chemotaxis system (Neuro Probe). The wells of the lower chambers were filled with 31 µl of buffer, fMLP or zymosan opsonized serum in various concentrations in buffer. Polycarbonate filters (3 micron) were positioned on the plate; and the differentiated PLB-985 cells (30 µl, 60,000 cells/well) were placed on the filter and allowed to migrate for 120 minutes at 37°C in 5% CO_2. Non-migrated cells were removed by gently wiping the filter with tissue. The fluorescence of cells in the filter was measured with a micro plate fluorescence reader with emission and expression wavelengths 485 and 530nm respectively, and the numbers of cells migrated into the filters was expressed as a percentage of numbers of cells added to the upper well.

Chemotaxis for cultured PLB-985 cells is shown in the figure 5, Appendix. PLB-985 cells (medium alone) as expected exhibited very low levels of motility in response to buffer (8-12%) or any concentration of fMLP or ZAS (8-12%). PLB-985 cells cultured with 30 ng/mL IFN-γ also showed very little movement into the lower chamber under conditions noted (7.0 – 15.7%). PLB-985 cells differentiated into a mature neutrophil phenotype over 4 days with DMSO expressed an increase in random migration ($p < 0.05$, paired t-test) as well as directed migration in response to 10^{-7} and 10^{-8} M fMLP ($p < 0.05$) and 5% and 10% ZAS ($p < 0.01$). No increase in motility was seen with 10^{-6} M fMLP. Dose response showed maximum directed movement at 10^{-8} M fMLP and 10% ZAS.

Addition of IFN-γ during the maturation of PLB-985 cells with DMSO showed a similar pattern. Random migration (buffer) was increased over media and IFN-γ alone ($p < 0.05$) and directed migration was increased with higher concentrations of fMLP and ZAS ($p < 0.01$). Motility after culture with DMSO plus IFN was significantly but not practically decreased only to 5% ZAS ($p < 0.05$).

These studies demonstrate that random and directed migration of neutrophils matured under the influence of IFN-γ are very similar to cells differentiated in the absence of this cytokine. These results are in contrast to previous studies with mature neutrophils exposed to IFN-γ and have implications for expanded use of IFN-γ in patient groups with neutrophil dysfunction, particularly those with altered motility.

Rationale for selected approach and trial design

IFN-γ-1b (Actimmune), a recombinant form of human interferon gamma is classified as a Type II interferon. It is FDA-approved for reducing the frequency and severity of serious infections associated with Chronic Granulomatous Disease (CGD) as well as indicated for delaying the time to disease progression in patients with severe, malignant

osteopetrosis (SMO). Both these conditions are life threatening, rare diseases. Because of these encouraging in vivo results noted, our team would like to better understand the in vivo functional and biochemical properties of IFN-γ in healthy subjects.

In this study, functional and biochemical observation of IFN-γ-1b will occur in two cohorts:

1. Single Dosing Cohort, which will examine the acute effects of IFN-γ-1b on the body's immune cells;
2. Steady State Cohort which will examine the phenotype of immune cells developing over time under the influence of IFN-γ-1b and to characterize the long term effects of this cytokine in vivo.

Participation Selection

Taking into consideration attrition and potential dropout rates, up to thirty (30) healthy adult volunteers will be screened and enrolled. A total of ten (10) subjects will be enrolled on the Single Dose (SD) cohort, and the potential of up to twenty (20) subjects will be enrolled on the Steady State (SS) cohort. The SD cohort will be the first to enroll. Ideally, we will offer the SD subjects enrollment onto the SS cohort upon completion of their SD regimen. However, we realize not all will volunteer to continue. At that time, the study team will seek additional recruitment for the SS cohort. Within the SS cohort, samples from the first ten subjects will be used for neutrophil analysis, and blood samples from the next ten participants will be used for steady state monocyte analysis.

Inclusion criteria

3.1.1 Healthy adults who ≥18 years up to 60 years;
3.1.2 At time of screening subject is well and healthy as deemed by PI;
3.1.3 At least 30 days from the last dose of IFN-γ 1b;
 3.1.3.1 If subject has previously participated in the SD cohort, then their 30-day post ADA must be reported as negative to be eligible for participation in the SS cohort.
3.1.4 Acute infections resolved and the subject is off treatment medications;
3.1.5 No diagnosis of chronic conditions or active health care issues for which the subject is actively followed by a health care provider and/or is on chronic medications;
3.1.6 Non-prescription medications for mild intercurrent illnesses will be allowed at the discretion of the PI.
3.1.7 Reproductive Function:
 3.1.7.1 All women of childbearing potential must have a negative urine beta-HCG.
 3.1.7.2 Female subjects of childbearing potential are required to practice two forms of effective contraception for the duration of their participation in the study.

Exclusion criteria

3.2.1 Pregnancy, breast-feeding, or unwillingness to use effective contraception during the study;
3.2.2 Recent vaccination (within the last fourteen days);
3.2.3 History of current infection and two weeks from most recent intercurrent infection;
3.2.4 History of recurrent infections or immunodeficiency.

Enrollment

3.3.1 Enrollment and screening (completed by Day 1)

3.3.2 Screened subjects will undergo a brief history and physical exam by the Principal Investigator.

3.3.3 All women of childbearing potential will have urine pregnancy testing within 72-hours of Day 1.

Registration Process

Consent and registration of all participants must occur before any study-related procedures. Staff will be available to register participants Monday thru Friday, from 8:00 AM to 5:00 PM.

Please follow these steps to register a participant:

1. Obtain written informed consent prior to any study related procedures.
2. Complete the eligibility checklist. The PI must sign the checklist confirming that eligibility has been reviewed.
3. The participant will be assigned a subject number that is unique.

Treatment Plan

In this study, IFN- γ-1b will be subcutaneously (SQ) administered to the right or left deltoid areas, or anterior thighs. A total of 30 subjects will be entered into one of two cohorts; Single Dose (SD) or Steady State (SS) dosing (see study schema below). Dosing of IFN- γ-1b will be based upon the time subject became eligible and started study. In this non-randomized, open-label study, subjects will be enrolled on the SD cohort first, and once that cohort has been filled, enrollment to the SS cohort will begin. Although not required, subjects in the SD cohort may also volunteer to participate in the SS cohort (granted they still meet eligibility criteria at the screening portion of the SS study). Since separate consents will be used for the SD and SS cohorts, any subjects volunteering to go onto the SS cohort will need to be re-consented to the SS consent. In the event not all the SD subjects choose to continue onto the SS cohort, we will plan to recruit new participants from our local campus community.

<u>Study Schema</u> (see next page):

Single Dose (SD) Cohort

(n=10)
10, 25, 50 and 100 µg/m² INF- γ-1b
administered once monthly
as per the following schedule:

Month 1 (Week 1) → 10 µg/m² INF- γ-1b
Month 2 (Week 5)→ 25 µg/m² INF- γ-1b
Month 3 (Week 9)→ 50 µg/m² INF- γ-1b
Month 4 (Week 13)→ 100 µg/m² INF- γ-1b

Steady State (SS) Cohort

for Neutrophil analysis

(n=10*)
50µg/m² INF- γ-1b given on Days 1, 3, 5 and 8

**Ideal enrollment goal would be that the previous 10 SD subjects consent and continue on to this SS Neutrophil Cohort. If not feasible, then study team will recruit new subjects to this cohort.*

Steady State (SS) Cohort

for Monocyte analysis

(n=10)
50µg/m² INF- γ-1b given on Days 1, 3, 5 and 8

Single Dose (SD) Cohort

Sample Size

Ten subjects will be enrolled to this cohort. IFN-γ1b will be administered subcutaneously every 28 days at escalating doses of 10, 25, 50 and 100 µg/m^2 at Weeks 1, 5, 9 and 13 respectively.

It is clear from earlier studies that one week is adequate time for washout of the drug and its biologic effects. In addition it is unlikely that the menstrual cycle in female subjects will influence any of the outcomes of interest.

Due to the blood volume required for the SD Cohort immuno-assays, and to allow adequate time for hematopoietic cell recovery, we are waiting 28-days (3 Weeks) before dosing the same study subject to the next IFN-gamma level. Additionally, to allow laboratory staff adequate time to perform these assays, enrollment of study subjects into the SD cohort will be staggered by one week. Although a separate Laboratory Manual for research staff includes a general roadmap of the study enrollment plan and what will occur each week, we have included in this protocol (Appendix IV) to illustrate the step-wise nature of accrual. For example, if Subject #1 on the SD cohort were to finish the 30-day follow-up ADA test (Week 17), the soonest they could participate in the SS cohort would be Week 35) – resulting in an 18 week "rest" period. The PI will initially offer Subjects 1 through 4 participation to the SS cohort first, with subsequent invitations to Subjects 5, 6, 7 etc… as they progress through the SD cohort.

Single Dose (SD) Cohort Time and Events Calendar

	Screening (Within 72-hours of Day 1, Week 1 administration)	Day 0 of Week 1 (0 hr.)	Week 1	Week 5	Week 9	Week 13	Week17 (+/-7days)
Consent, eligibility screening	X						
IFN-γ-1b (µg/m^2)			10	25	50	100	
Blood Samples*		X	X$	X$	X$	X$	X
Adverse Event Assessment		X	X	X	X	X	X

***Refer** to Table 1 (below) for specimen collection volume and Section 5.1 for specimen processing instructions.

$Blood will be taken just prior to and at 4, 8, 12, 24, 48, 72, and 96-hours post Day 1 IFN-γ1b administration on dosing Weeks 1, 5, 9 and 13.

Laboratory Studies:

Blood samples will be obtained for the following studies at the times before, and/or after IFN-γ administration as noted below:

Plasma Specimens: Anticoagulated specimens will be obtained at 0, 4, 8, 12, 24, 36, 48, 72, and 96 hours (+/- 30 minutes) on dosing Weeks 1, 5, 9 and 13. The cells will be separated immediately by centrifugation and plasma frozen and stored at -70 ^{0}C. IFN-γ levels will be run on 0, 4, 8, 12, and 24 hours samples at the lab identified by the sponsor for analysis. IP10 and Neuropterin, proteins expressed under the influence of IFN will be run on all plasma samples.

Gene Expression Analysis: Will be obtained at 0, 4, 8, 12, 24, 36, 48, 72 and 96 hours on dosing Weeks 1, 5, 9 and 13. Anticoagulated specimens will be obtained, neutrophils and mononuclear cells will be isolated, and the RNA extracted by standard techniques with storage at -70 ^{0}C. The RNA from neutrophils will be used in Affimetrix Gene Chip studies to define changes in genomic expression. RNA from mononuclear cells will be stored for future analysis within the study time period.

Neutrophil Nox2 Activity: Will be measure at 0, 8, 24, 48, 72, 96 hours on Weeks 1, 5, 9 and 13. Anticoagulated specimens will be obtained, neutrophils isolated, and Nox2 activity measured as SOD inhabitable cytochrome c reduction and oxidation of dihydrorhodamine (see below). Cell lysates for neutrophils and mononuclear cells will also be prepared and frozen at -70 ^{0}C. Depending on results, analysis for phox proteins and other relevant marker proteins (e.g. frataxin) will be completed.

Interferon-gamma Anti-drug Antibody (ADA) Testing: We will collect serum for immunogenicity assessment, as IFN-γ 1b is highly homologous to endogenous human interferon-gamma. Samples will be collected from subjects before the first dose, 7 days after each dose of IFN-γ 1b, and 30-days after the last dose of IFN-γ 1b in the SD cohort. We will also complete ADA testing before the first dose, 7-days after the 4th dose, and 30-days after the last dose of IFN-γ 1b in the SS cohort.

If subjects enrolled on the SD cohort, wish to participate in the SS cohort, their 30-day post ADA result must have been reported as negative in order to qualify for participation on the SS cohort.

The ADA samples will be sent to an outside Central Laboratory for analysis of antibodies that include IgA, IgG and IgM, with validated detection, confirmatory titration and neutralizing assays. If antibodies are detected, subjects will be removed from the study and followed with careful monitoring and subsequent monthly sampling to document when levels have returned to baseline. Please refer to the separate Laboratory Manual for complete collection and processing instructions.

(Table 1) Single Dose (SD) Cohort Laboratory Collection Volumes per Subject:

Week	Day	IFN- γ-1b dose	Time point	Lavender EDTA vaccutainer tube*	Lithium Heparin Green Top**	Red Top***	Blood Total (mL)
Week 1	Day 1	10 μg/m2	0 hr. (prior to IFN- γ-1b administration)	4	20	3	27
			4 hr. (post administration)	4	20	-	24
			8 hr.	4	20	-	24
			12 hr.	4	20	-	24
	Day 2		24 hr.	4	20	-	24
	Day 2		36 hr.	4	20	-	24
	Day 3		48 hr.	4	20	-	24
	Day 4		72 hr.	4	20	-	24
	Day 5		96 hr.	4	20	-	24
	Day 8					3	3
Week 5	Day 1	25 μg/m2	0 hr. (prior to IFN- γ-1b administration)	4	20	-	24
			4 hr. (post administration)	4	20	-	24
			8 hr.	4	20	-	24
			12 hr.	4	20	-	24
	Day 2		24 hr.	4	20	-	24
	Day 2		36 hr.	4	20	-	24
	Day 3		48 hr.	4	20	-	24
	Day 4		72 hr.	4	20	-	24
	Day 5		96 hr.	4	20	-	24
	Day 8			-	-	3	3
Week 9	Day 1	50 μg/m2	0 hr. (prior to IFN- γ-1b administration)	4	20	-	24
			4 hr. (post administration)	4	20	-	24
			8 hr.	4	20	-	24
			12 hr.	4	20	-	24
	Day 2		24 hr.	4	20	-	24
	Day 2		36 hr.	4	20	-	24
	Day 3		48 hr.	4	20	-	24
	Day 4		72 hr.	4	20	-	24

Week	Day	IFN- γ-1b dose	Time point	Lavender EDTA vaccutainer tube*	Lithium Heparin Green Top**	Red Top***	Blood Total (mL)
	Day 5		96 hr.	4	20	-	24
	Day 8			-	-	3	3
Week 13	Day 1	100 μg/m^2	0 hr. (prior to IFN-γ-1b administration)	4	20	-	24
			4 hr. (post administration)	4	20	-	24
			8 hr.	4	20	-	24
			12 hr.	4	20	-	24
	Day 2		24 hr.	4	20	-	24
	Day 2		36 hr.	4	20	-	24
	Day 3		48 hr.	4	20	-	24
	Day 4		72 hr.	4	20	-	24
	Day 5		96 hr.	4	20		24
	Day 8			-	-	3	3
Week 17 (+/- 7 days)				-	-	3	3
				144 mL	720 mL	18 mL	882 mL

***** IFN-gamma levels, IP10, and Neuropterin assays
******Gene expression analysis, and Neutrophil Nox2 activity assays
******* Immunogenicity testing

Statistical Analysis: See Section below, Statistical Considerations

Steady State (SS) Cohort

Sample Size:

Up to twenty subjects will be enrolled to this cohort. IFN-γ-1b will be administered at a fixed dose of 50 μg/m^2 subcutaneously on Days 1, 3, 5, and 8.

Steady State (SS) Cohort

	Screening (Within 72-hours of Day 1, Week 1 administration)	Day 1	Day 3	Day 5	Day 8	Day 15 (+/-3 days)	Day 30 (+/- 7 days)
Consent, eligibility screening	X						
IFN-γ-1b (µg/m^2)		50	50	50	50		
Blood Samples*		X (Pre-dose)			X (prior to 4th dose)	X	X
Adverse Event Assessment		X	X	X	X	X	X

*Refer to Section 5.1 for specimen collection and processing instructions.

The SS Cohort is further sub-divided into either neutrophil or monocycle analysis. The rationale for this is because monocyte and neutrophil studies cannot be conducted simultaneously on one subject due to blood volume collection restrictions. Additionally, because both neutrophil and monocyte assays require fresh blood, there are limited laboratory personnel to perform both at the same time. As such, these cohorts will require the proposed sample size of twenty; ten subjects for neutrophil studies and ten subjects for monocyte studies, as well as staggered enrollment period with initial accrual to SS neutrophil cohort, and then accrual to the SS monocyte cohort.

Planned enrollment on to the SS cohort will occur *after* completion of the SD studies.

Laboratory Studies:

Peripheral blood will be obtained Days 1 and 8 prior to IFN-γ-1b administration. These samples will be used for neutrophil or monocyte studies and a Complete Blood Count (CBC). In addition, follow-up ADA immunogenicity testing will done 7 days (+/-3) from the 4th dose, and 30-days (+/-7) from the last dose of IFN-γ-1b administered. See tables below.

Steady State (SS) Cohort Laboratory Collection Schedule for neutrophil analysis:

Day	Time point	CBC	Neutrophil function biochemical studies	Neutrophil Gene expression analysis	ADA Immunogenicity Testing	Blood Total (mL)
1	0 hr. (prior to IFN-γ administration)	3 mL lavender top	50-75 mL* Lithium heparin green top		3 mL in Red Top	56-80 mL*
8	Prior to IFN-γ administration	--	50-75 mL* Lithium heparin green top			50-75 mL*
15 (+/-3)		---	---		3 mL in Red Top	3 mL
30 (+/-7)		---	---		3 mL in Red Top	3 mL

Total Volume = 112 – 162 mL

*Volume of blood dependent on body weight

Steady State (SS) Cohort Laboratory Collection Schedule for monocyte analysis:

Day	Time point	CBC	Monocyte function biochemical studies	Monocyte Gene expression analysis	ADA Immunogenicity Testing	Blood Total (mL)
1	0 hr. (prior to IFN-γ-1b administration)	3 mL lavender top	50-75 mL* Lithium heparin green top		3 mL	56-80 mL*
8	Prior to IFN-γ-1b administration	--	50-75 mL* Lithium heparin green top			50-75 mL*
15 (+/-3)		---	---		3 mL	3 mL
30 (+/-7)		---	---		3 mL	3 mL

Total Volume = 112 – 162 mL

*Volume of blood dependent on body weight

Neutrophil function and biochemistry assays include:

Function studies	Biochemical studies
Adherence	f-Actin assembly
Chemotaxis	CD11b/CD18 expression
Ingestion	CD 14, 16, 178, 177 FcγR (CD64) expression
Bactericidal activity	Nox2 activity
Degranulation activity	Cell lysates for phox protein, granule constituents

Gene expression studies:

On Days 1 and 8, RNA will be extracted from neutrophils. The RNA will be used in Affimetrix gene chip studies to define changes in genomic expression associated with IFN-γ administration.

Monocyte function studies will be completed before administration of a first dose of IFN-γ as noted above and on Day 8 after the 4th dose. Samples will be obtained on Day 1 and 8 as described for neutrophil studies and monocyte studies completed as listed below.

Monocyte Function and biochemistry assays:

Functional Studies	Biochemical Studies
Adherence	Expression of CD11a, b, c, CD64, CD14, CD16, CCR2, CX3CR1, PSGL-1, PECAM-1, CD177, 178
Chemotaxis	Nox2 activity
Ingestion	Cell lysates for granule proteins, phox proteins
Microbicidal activity	ADCC

Gene expression studies:

On Days 1 and 8, RNA will be extracted from monocytes. The RNA will be used in Affimetrix gene chip studies to define changes in genomic expression associated with IFN-γ-1b administration.

Statistical Analysis: See section below, Statistical Considerations.

Participants will be recruited locally through COMIRB-approved flyers distributed throughout campus, as well as listing on campus-wide "Available Clinical Trials" emails and website.

Specimen Collections, Labeling and Storage

Labeling: Each specimen should be labeled with the patient ID number along with the date and time the specimen was collected.

Specimen processing and Storage: All specimens will be transferred to the Immunohematology Laboratory Monday thru Friday for processing. Please refer to detailed sample processing instructions in separate, Protocol Lab Manual.

Duration of Study

Duration of therapy will be dependent on the cohort in which each is enrolled. Participants enrolled in the Single Dose Cohort will participate for approximately 21 weeks. Participants enrolled on the Steady State Cohort will participate for approximately 45 days. AEs will be followed for 30 days past the last dose of study drug for both cohorts.

Participant Stopping Criteria

Participants will be removed from the study once they have completed their assigned cohort, or for any of the following reasons:

- Request to discontinue study by participant;
- PI clinical discretion such as:
  - Poor compliance with clinic visits and blood draws;
  - Low leukocyte count (below 3500 cells/µL)which would require the need for greater volume of blood draws to meet testing needs;
  - Positive immunogenicity testing at any point during study participation.
  - Change in health conditions so that subject would no longer meet inclusion or exclusion criteria;
- Severe Adverse Event (e.g., severe neurologic, bone marrow or liver toxicities)

DRUG INFORMATION FOR IFN-γ lb (Acctimmune®)

Dosage:

Single Dose (SD) Cohort: IFN-γ lb administered at the following increasing doses: 10, 25, 50 and 100 micrograms/m^2/dose on Weeks 1, 5, 9 and 13 respectively.

Stead State (SS) Cohort:

SS *neutrophil* Cohort:	IFN-γ lb administered on Days 1, 3, 5, and 8 at a fixed dose of 50 micrograms/m^2/dose.
SS *monocyte* Cohort:	IFN-γ lb administered on Days 1, 3, 5, and 8 at a fixed dose of 50 micrograms/m^2/dose.

Route of delivery:

Subcutaneous injection in right or left deltoid, or anterior thighs.

Adjustment of Dosing:

No dose adjustments in this protocol.

Availability:

IFN-γ lb will be provided by Horizon Pharma Ireland Ltd. to study participants at no cost.

Accountability:

Drug accountability will be maintained through the University of Colorado Hospital Investigational Pharmacy.

Source and Pharmacology:

ACTIMMUNE® (Interferon gamma-1b), a biologic response modifier, is a single-chain polypeptide containing 140 amino acids. Production of ACTIMMUNE is achieved by fermentation of a genetically engineered Escherichia coli bacterium containing the DNA, which encodes for the human protein. Purification of the product is achieved by conventional column chromatography

Interferons bind to specific cell surface receptors and initiate a sequence of intracellular events that lead to the transcription of interferon-stimulated genes. Specific effects of interferon-gamma include the enhancement of the oxidative metabolism of macrophages, antibody dependent cellular cytotoxicity (ADCC), activation of natural killer (NK) cells, and the expression of Fc receptors and major histocompatibility antigens.

Toxicity:

The most common side effects with ACTIMMUNE® are "flu-like" symptoms such as fever, headache, chills, myalgia (muscle pain), or fatigue, which may decrease in severity as treatment continues. The following table was taken from the most recent ACTIMMUNE Investigator's Brochure (Horizon Pharma, Edition 1.0)

Percent of Patients **Clinical Toxicity**	***ACTIMMUNE*** **CGD (n=63)**	**Placebo CGD (n=65)**
Fever	52	28
Headache	*33*	*9*
Rash	*17*	*6*
Chills	*14*	*0*
Injection site erythema or tenderness	*14*	*2*
Fatigue	*14*	*11*
Diarrhea	*14*	*12*
Vomiting	*13*	*5*
Nausea	*10*	*2*
Myalgia	*6*	*0*
Arthralgia	*2*	*0*
Injection site pain	*0*	*2*

Formulation and Stability:

ACTIMMUNE is supplied as a sterile, clear, colorless, preservative-free solution filled in a glass vial with a rubber closure. Each 0.5-mL vial of ACTIMMUNE solution delivers 100 μg (2 million IU/0.5 mL) of IFN-γ lb, formulated in mannitol, sodium succinate, succinic acid, polysorbate 20, and Sterile Water for Injection, United States Pharmacopeia (USP).

IFN-γ 1b solution does not contain a preservative. A vial of ACTIMMUNE solution is suitable for single use only. The unused portion of any vial should be discarded.

Vials of IFN-γ 1b must be placed in a 2°C to 8°C (36°F to 46°F) refrigerator immediately upon receipt to ensure optimal retention of physical and biochemical integrity. The vials must not be frozen. Excessive or vigorous agitation should be avoided. Exposure of vials to temperatures greater than 25°C (77°F) should be strictly avoided. Vials left at room temperature (>8°C) for a total time exceeding 6 hours should be returned to the sponsor or designee. Vials should not be used beyond the expiration date.

Drug Preparation:

No preparation required.

Protocol Workflow

The anticipated sequence of studies is as follows:

(1) Planning, submission to and approval by COMIRB and Colorado Clinical Translational Sciences Institute (CCTSI), establishment of sample handling logistics, and confirmation of all techniques (3 months);
(2) Completion of SD studies on 10 subjects (10 months);
(3) Completion of SS studies for neutrophil function and biochemistry on 10 subjects (5 months); and
(4) Completion of SS studies for monocyte function and biochemistry on 10 subjects (5 months).

Experimental Methods

Neutrophils and monocytes will be isolated from heparinized peripheral blood by standard techniques. Phagocytosis will be completed with fluorescent zymosan opsonized with IgG or serum (C3b). Adherence and chemotaxis will be measured in an under agarose assay or in a 96-well chamber through a polycarbonate filter with C5a (zymosan activated serum, 5%) and fMLP (0.1 µM) as chemo attractants. Bactericidal activity will be measured against *S.aureus* or *E. coli* opsonized with normal human serum, and ingestion can be determined by visual inspection of samples from early time points and quantitating the % of cells which have intracellular microbes. Nox2 activity will be determined as SOD inhibitable cytochrome c reduction and oxidation of dihydrorhodamine as described above in response to PMA (200 nM) and fMLP (1 µM). We will also evaluate expression of Nox2 proteins, gp91phox, p40phox, p47phox, p67phox, Rac2, and Prdx6 by Western Blot and correlate these results to Nox2 activity. CD11b/CD18 and other surface markers and f-Actin assembly will be measured by flow cytometry. All of these techniques are currently employed in our laboratory.[3-15] ADCC for monocytes will be determined by release of LDH from antibody (anti-RhD) coated

Rh(D) positive red blood cells. IP10 ,neuropteran, and frataxin will be determined by ELISA techniques. RNA will be extracted from neutrophils and monocytes as above and transcriptome analysis will be completed on Affimetrics gene chips in the Genomics and Microarray Core Laboratory, University of Colorado Cancer Center. Correlation between functional characteristics and Genomic expression data will be evaluated with standard analytical and statistical packages.

Operational and Statistical Considerations

Sample size justifications.

IFN-γ investigated in this protocol has been used for >20 years in patients with CGD and osteopetrosis. Benefits are well defined and the risk profile is low in the doses used including those proposed here. No current data exists for the in vivo biochemical, molecular and cell function data that will be measured in this proposal. However, our in vitro culture model provides reasonable data on Nox2 activity and phox protein levels, which approximates what we expect to see in vivo and to justify sample size. The difference in Nox2 activity and phox protein levels between IFN-γ treated and the controls are at least 2.5 fold of the common standard deviation. With 10 subjects in each group the study provides 80% power to detect a difference of 1.3 common standard deviation between the groups with a two-sided t-test with a significance level of 0.05. Thus, to be conservative to assume that human samples are more variable, it appears that 10 subjects in each group for the studies proposed will provide interpretable results.

Adverse Events related to IFN-γ lb

The benefits of IFN-γ lb are related to its immunostimulatory activity. Its toxicity profile is low and has been well defined since its use in patients with CGD established in 1991. IFN-γ lb is contradicted in those individuals who develop or have had known hypersensitivity reactions to IFN-γ lb, *E. coli* derived constituents, or components of the product.

Severe cardiovascular or neurologic disorders or bone marrow and liver toxicities have been seen at daily doses two and a half times greater than the single dose or three times a week dosing described in this protocol. Most are reversed with dose reduction or stopping the drug. The clinical safety of IFN-γ 1b has been extensively studied both as a single agent and as adjunctive treatment, in normal volunteers and in patients with diverse medical conditions. In addition, both adult and pediatric populations have been studied. Routes of administration have included IV, SC, and IM of IFN-γ 1b. Dosing regimens have included daily, three times weekly, and once weekly by the SC route, daily by IM injection, and daily or twice weekly by the IV route.

As with other interferons, the most common adverse experiences in clinical trials evaluating IFN-γ 1b have been “flu-like” or constitutional symptoms (FLS) such as fever, inflluenza-like symptoms, and headache. These FLS are typically of mild-to-moderate severity. A healthy volunteer study showed attenuation of FLS when ACTMMUNE is titrated step-wise over 2 weeks. As in trials of patients with CGD, there was no evidence of irreversible clinical laboratory abnormalities that were considered by the investigator to be related to IFN-γ 1b.

Based on the data obtained from the clinical trial with CGD patients, which used the same dose, and schedule proposed here, the incidence of adverse events (AEs) is low. The most commonly reported adverse events included mild fever, headache, rash, chills, and injection site erythema and tenderness. These AEs decreased in severity and frequency as IFN-γ treatments continued and were ameliorated by administration of acetaminophen. Other symptoms such as fatigue, diarrhea, vomiting, nausea, myalgia, and arthralgia occurred at the same rate as placebo or were related to the underlying disorder and/or its complications.

The rate of neutralizing anti-drug antibodies to IFN-γ is very low. Based on results from 334 patients treated for short courses with IFN-γ in Phase I and II trials, one patient developed a non-neutralizing antibody. No severe clinical adverse events have been attributed to immune reactivity of this drug.

Supportive Care Guidelines for expected AEs:

AEs will be assessed from the time first dose of IFN-γ Ib is administered through 7 days after the last dose of IFN-γ Ib. The Principal Investigator will thoroughly discuss with each subject the expected adverse events associated with IFN-γ Ib administration as well as providing guidelines for supportive care measures. This will include the recommendation of acetaminophen: 325-650 mg every 4-6 hours. Subjects will also be provided with study doctor's contact information in the event an AE were of concern (e.g., temperature greater than 40 degrees Celsius [104 degrees Fahrenheit]).

Adverse Event Reporting Requirements

Adverse events (AEs) that occur while the patient is on study will need to be recorded. There are specific reporting requirements for AEs, which are listed in the table below. Additionally, certain adverse events must be reported in an expedited manner to allow for timelier monitoring of patient safety and care. The following sections provide information and about expedited reporting through the use of a written IND safety report (MedWatch) to the Food and Drug Administration (FDA).

All adverse events will be evaluated according to the "Toxicity Grading Scale for Healthy Adults and Adolescent Volunteers Enrolled in Preventive Vaccine Clinical Trials" (Appendix II).

Adverse events will be monitored starting at the time the participant signs consent and ending 30 days after the participant's last dose of IFN-γ Ib. All toxicities that are a grade 2 and more severe will be recorded in the AE log.

	When to report AEs to COMIRB				
**Attribution**	Grade 2 & 3 AE Expected	Grade 2 & 3 AE Unexpected	Grade 4 AE Expected	Grade 4 AE Unexpected	Grade 5 AE Expected or Unexpected
Unrelated Unlikely	Not required	Not required	Report within 3 days	Report within 3 days	Report within 24 hours
Possible Probably Definite	Not required	Report within 3 days	Report within 3 days	Report within 3 days	Report within 24 hours

Adverse Events Definitions

Adverse Event (AE): An adverse event means any untoward medical occurrence associated with the use of a drug in humans, whether or not considered drug related.

Suspected Adverse Reaction: Any adverse event for which there is a reasonable possibility that the drug caused the adverse event. Reasonable possibility means there is evidence to suggest a causal relationship between the drug and the adverse event.

Unexpected Adverse Event or Unexpected Suspected Adverse Reaction: An adverse event or suspected adverse reaction is considered "unexpected" if it is not listed in the investigator brochure or is not listed at the specificity or severity that has been observed; or, if an investigator brochure is not available, is not consistent with the risk information described in the general investigational plan.

Serious Adverse Events (SAE) or Serious Suspected Adverse Reactions: An adverse event
or suspected adverse reaction is considered serious if, in the view of either the investigator or sponsor, it results in any of the following outcomes:

**Death of Patient**	An event that results in the death of a patient.
**Life-Threatening**	An event that, in the opinion of the investigator, would have resulted in immediate fatality if medical intervention had not been taken. This does not include an event that would have been fatal if it had occurred in a more
**Hospitalization**	An event that results in an admission to the hospital for any length of time. This does not include an emergency room visit or admission to an
**Prolongation of Hospitalization**	An event that occurs while the study patient is hospitalized and prolongs the patient's hospital stay.
**Congenital Anomaly**	An anomaly detected at or after birth or any anomaly that result in fetal loss.
**Persistent or Significant Disability/ Incapacity**	An event that results in a condition that substantially interferes with the activities of daily living of a study patient. Disability is not intended to include experiences of relatively minor medical significance such as headache, nausea, vomiting, diarrhea, influenza, or accidental trauma (*e.g.,*
**Important Medical Event Requiring Medical or Surgical Intervention to Prevent Serious Outcome**	An important medical event that may not be immediately life-threatening or result in death or hospitalization, but based on medical judgment may jeopardize the patient and may require medical or surgical intervention to prevent any of the outcomes listed above (*i.e.,* death of patient, life-threatening, hospitalization, prolongation of hospitalization, congenital anomaly, or persistent or significant disability/incapacity). Examples of such events include allergic bronchospasm requiring intensive treatment in an emergency room or at home, blood dyscrasias or convulsions that do not result in inpatient hospitalization, or the development of drug dependency or drug abuse.

Expedited Adverse Event Reporting to the FDA

Per CFR 312.32 (c), the sponsor of the IND, Daniel Ambruso, MD, will notify the FDA and all participating investigators (if applicable) in a written IND safety report of any adverse experience **associated with use of the drug** that is **both serious and unexpected.** Each written notification shall be made as soon as possible, and in no event later than **15 calendar** days after the sponsor's initial receipt of the information. Each written notification may be submitted on FDA Form 3500A (MedWatch) or in a narrative format and must bear prominent identification of its contents, i.e., "IND Safety Report". Follow-up information to a safety report should be submitted as soon as the relevant information is available.

Expedited safety reporting must also be followed per COMIRB guidelines. That is, initial reporting of a serious, unexpected adverse event that is likely or probably related to study drug, must be reported within 5 business days. Please refer to COMIRB Policy & Procedures for complete reporting algorithm.

The sponsor must also notify FDA **by telephone** or by **facsimile** transmission of any **unexpected fatal or life-threatening experience associated with use of the drug** in the clinical studies conducted under the IND as soon as possible but in no event later than **7 calendar** days after initial receipt of the information.

Each telephone call or facsimile transmission to the FDA shall be transmitted to the FDA division that has responsibility for review of the IND; a specific contact person is assigned to each IND at the time the application is filed, and this will be included in the FDA's correspondence acknowledging receipt of the IND application.

Regulatory Requirements

Informed Consent

All participants must be provided a consent describing each stratum of this study. All participants must be provided with sufficient information to make an informed decision about their participation in this study. The formal consent of a participant, using the IRB approved consent form, must be obtained before the participant is involved in any study-related procedure. The consent form must be signed and dated by the participant or the participant's legally authorized representative, and by the person obtaining the consent. The participant must be given a copy of the signed and dated consent document. The original signed copy of the consent document must be retained in the medical record or research file.

Study Documentation

The investigator must prepare and maintain adequate and accurate source documents designed to record all observations and other data pertinent to the study for each research participant. This information enables the study to be fully documented and the study data to be subsequently verified.

Data and Safety Monitoring

Data Safety and Monitoring Plan

The principal investigator (PI) will be responsible for monitoring the activities of the proposal per the trial-monitoring plan, in addition to overseeing the safety and efficacy of the trial, executing the Safety Officer plan, and complying with all the reporting requirements to local and federal authorities. This oversight will be accomplished through the PI and a selected Safety Officer. The PI and Safety Officer are responsible for ensuring data quality and patient safety. A summary of their responsibilities is as follows:

- Conduct internal audits
- Ongoing review of all SAEs, UAPs and reportable AEs
- Has the authority to close and/or suspend trial for safety or trial conduct issues

Reports will include 6 monthly summaries of enrollment numbers, toxicity data including SAEs and AEs, protocol modifications, protocol deviations, and amendments. Per University of Colorado (UCD) requirements, SAEs, Unanticipated Problems (UAPs) and reportable AEs will be reported to the COMIRB within 5 business days of receiving notification of the occurrence. The PI will provide a summary of AEs annually to COMIRB at continuing review. The PI will also submit annual progress reports within 60 days of the anniversary of the date that the IND became active per [21 CFR 312.33].

In addition to patient safety issues, the PI is responsible for ensuring data quality. Data will be entered into the database (Redcap database, Redcap.ucdenver.edu) by laboratory technical staff on laboratory computers. Only the PI and approved technical staff will have access to the database. Any related paper records will be kept in locked cabinets within the laboratories, which are locked and have limited access except to approved study personnel.

Statistical Analysis Plan

Plans for analyzing the data collected from this proposal are summarized in the table below.

Phase	Assay Data	Statistical analysis
Single dose (SD) Cohort	Plasma IFN, IP10, neuropteran	Paired t-test (pre- and post) and unpaired t-test (two independent groups). Regression analysis over time using growth curve model.
	Nox2 activity, phox and other proteins	As per above.
Steady state (SS) Cohort	Adherence, chemotaxis, ingestion, bactericidal activity, CD11b/18 expression, Nox2 activity, degranulation and cell proteins	Paired t-test (Day 1 vs. Day 8)
SD and SS Cohorts	Gene expression studies	Analysis of gene expression is summarize in the notes below.[1]

[1] Log_2 transformed will be obtained from the Affimetrix Transcriptome Analysis Console (ver. 2.0.0.9). Relative expression levels will be compared using unpaired t-test of all genes to detect genes differentially expressed as having a 2-fold over- or under-expression with an associated p-value < 0.05. These will be further adjusted for false discovery at a FDR less than 0.05 using the Benjamini-Hochberg method. Subsequent observational analysis for patients at different time points and over different doses, when applicable will help define changes seen in gene expression. Further statistical analysis will then be developed for the data set with formal statistical consultation as there are currently no known approaches for this large amount of data and a unique approach will need to be developed.

References

1. Ellis,TN et. al. Immunology 2004; 112: 2-12.
2. Ambruso DR, Johnston RB, Jr: Primary Immunodeficiency: Chronic Granulomatous Disease and Common Variable Immunodeficiency Disorders. In Kendig and Chernick's Disorders of the Respiratory Tract in Children (8th Edition). RW Wilmott, TF Boat, A Bush, V Chernick, R Deterding, F Ratjen (eds), WB Saunders Co., 2012.
3. Leavey PJ, et. al. J Biol Chem 2002; 277:45181-45187.
4. Ambruso DR, et. al. J Biol Chem, 1990; 265:924-930. Corrections: J Biol Chem 1990; 265:19370-19372.
5. Ambruso DR, et. al. Biochim Biophys Acta 2012; 1823: 306-315.
6. Ellison MA, et. al. Eur J Immunol 2012; 42:1609-1617.
7. Ambruso DR, et. al. J Clin Immunol 1984;4: 23-30.
8. Leavey PJ et. al. Blood 1998; 92: 4366-4374.
9. Ambruso DR, et. al. Proc Natl Acad Sci USA 2000;97:4654-4659.
10. Kurkchubasche AG, et. al. J Pediatr 2001;139:141-147.
11. Silliman CC, et. al. J Leukoc Biol 2003; 73: 511-524.
12. Frevert CW, et. al. J Immunol Methods 1998; 213: 41-52.
13. Marko V, et. al. Appl Environ Microbiol 1998; 64: 515-519.
14. Speer CP, et. al. infect Immun 1985; 50:919-921.
15. Chi C and Pamer EG. Nat Rev Immunol 2011;11:762-774.
16. Tovey et al. Pharmaceuticals 2010;3:1162-1186
17. Jaffe et al. J of Biological Response Modifiers 1987;6:576-580
18. FDA Guidance for Industry: Toxicity Grading Scale for Healthy Adult and Adolescent Volunteers Enrolled in Preventative Vaccine Clinical Trials. http://www.fda.gov/BiologicsBloodVaccines/GuidanceComplianceRegulatoryInformation/Guidances/Vaccines/ucm074775.htm
19. FDA Guidance for Industry: Assay Development for Immunogenicity Testing of Therapeutic Proteins. http://www.fda.gov/downloads/drugs/guidancecomplianceregulatoryinformation/guidances/ucm192750.pdf

Reimbursement of Study Subjects

Subjects will be reimbursed $600 for completion of the single dose studies and $400 for the steady state study. Payment in the SD cohort will be prorated for each dose given and lab draws obtained. The SS cohort subjects will be paid in full on Day 8 of their participation. Reimbursement includes subject time for consenting/enrolling, screening, receiving injections of IFN-γ lb, phlebotomies and AE assessments.

Materials Provided by Horizon Pharma Ireland Ltd.

- IFN-gamma: Provided by Horizon Pharma Ireland Ltd.
- Plasma levels of IFN-γ will be completed by KCAS Laboratories, Kansas City, Kansas, a contracted laboratory for Horizon Pharma Ireland Ltd.

APPENDIX I

Figure 1
Figure 2
Figure 3
Figure 4
Figure 5
Figure 6

Figure 1

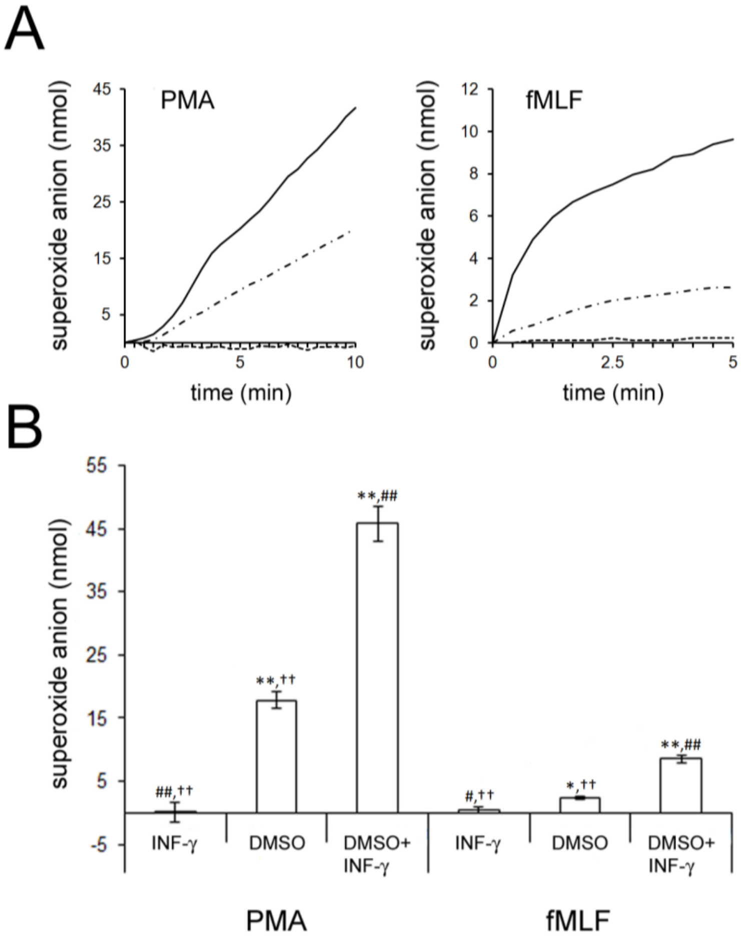

Figure 2

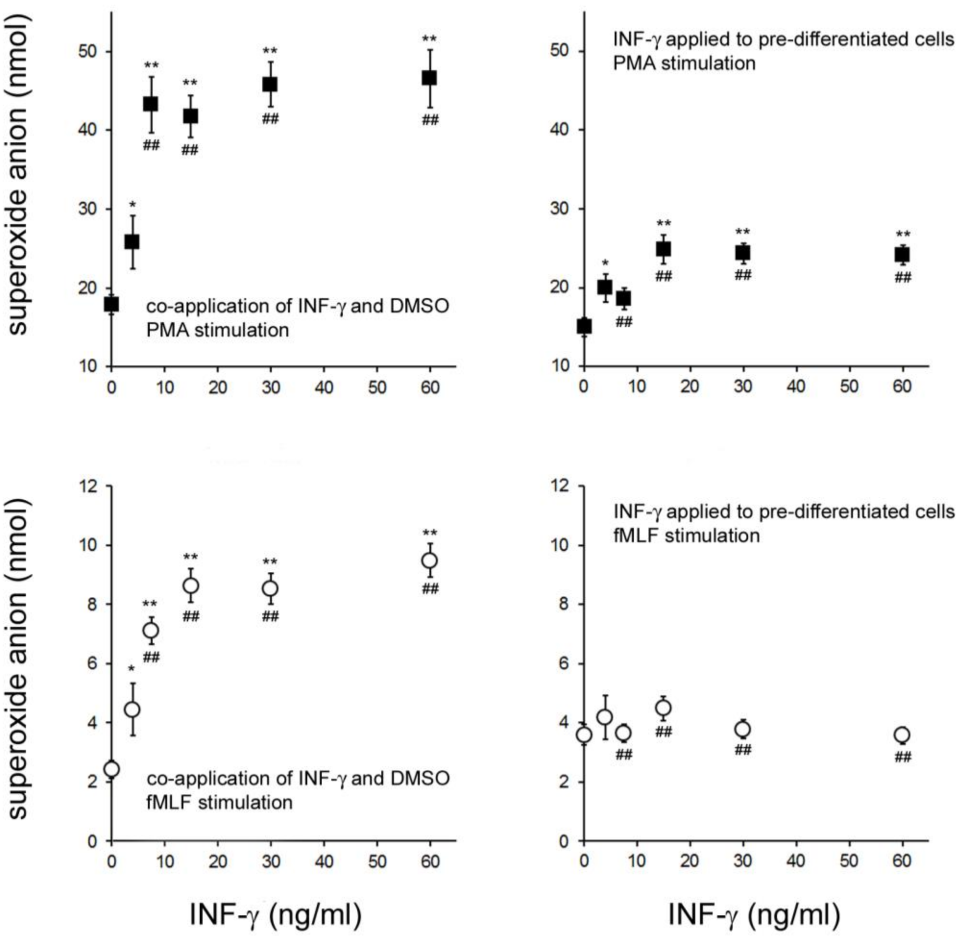
superoxide anion (nmol)
co-application of INF-γ and DMSO
PMA stimulation
INF-γ applied to pre-differentiated cells
PMA stimulation
co-application of INF-γ and DMSO
fMLF stimulation
INF-γ applied to pre-differentiated cells
fMLF stimulation
INF-γ (ng/ml)
INF-γ (ng/ml)

Figure 3

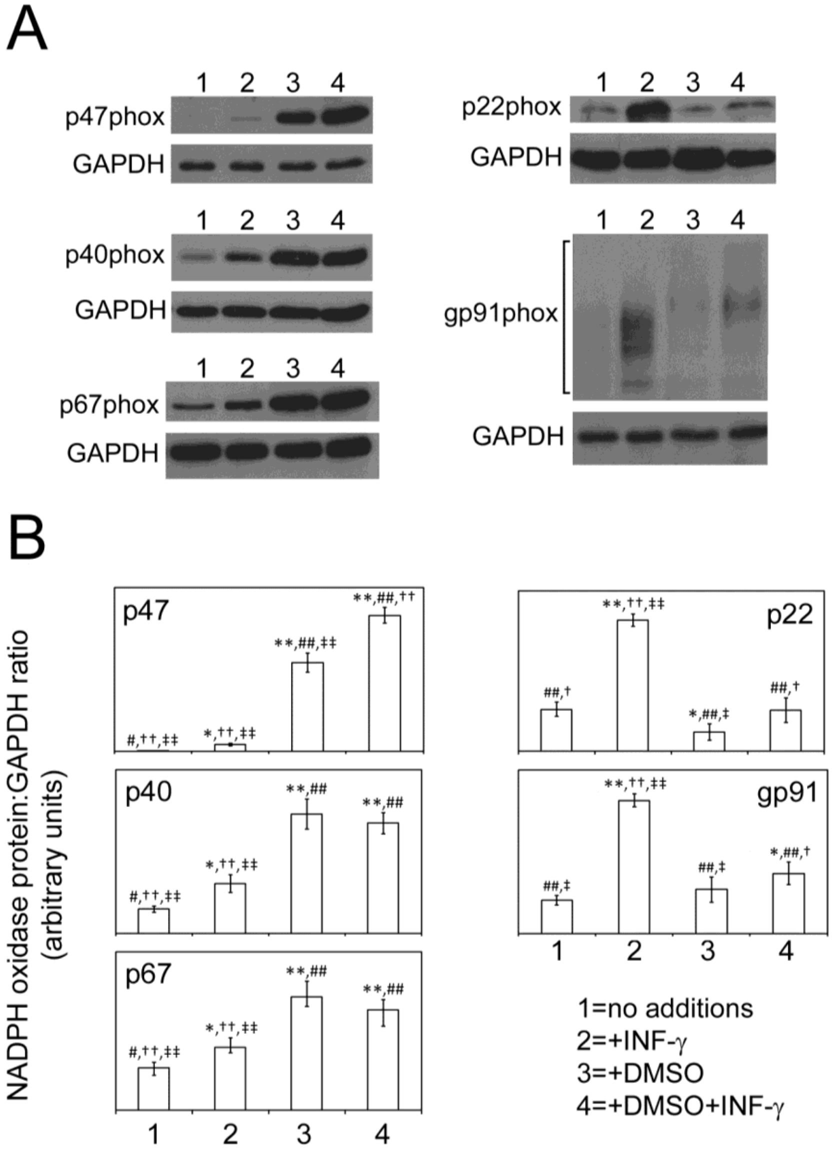
A
1 2 3 4
p47phox
GAPDH
p40phox
GAPDH
p67phox
GAPDH
p22phox
GAPDH
gp91phox
GAPDH
B
NADPH oxidase protein:GAPDH ratio (arbitrary units)
p47
p40
p67
p22
gp91
1=no additions
2=+INF-γ
3=+DMSO
4=+DMSO+INF-γ

Figure 4

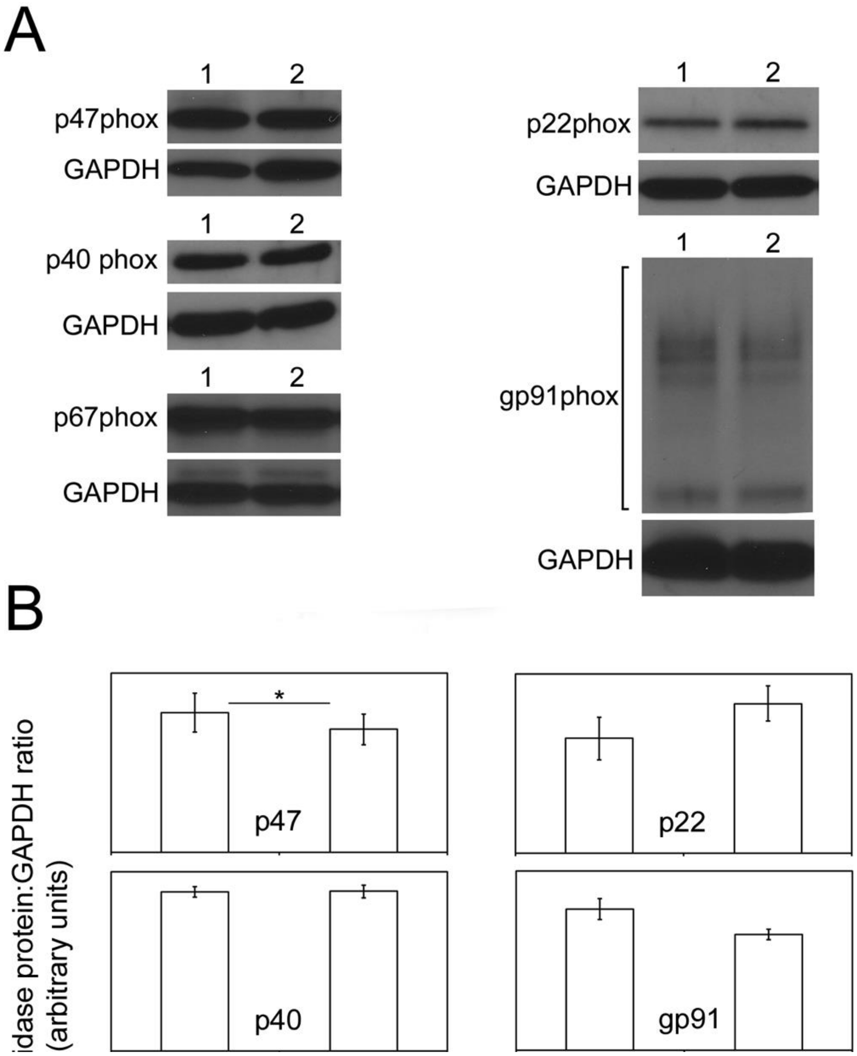

Figure 5

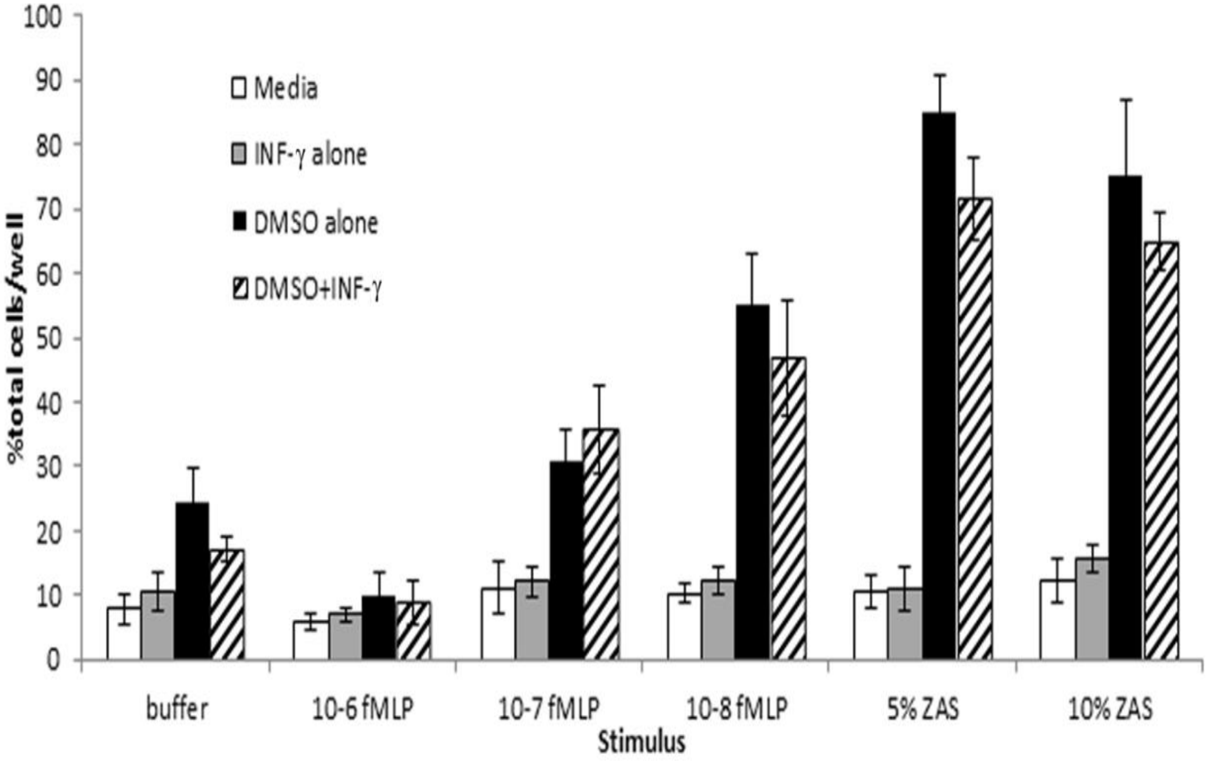

Appendix II: Toxicity Grading Scale for Healthy Adult and Adolescent Volunteers Enrolled in Preventative Vaccine Clinical Trials

Local Reaction to Injectable Product	Mild (Grade 1)	Moderate(Grade 2)	Severe (Grade 3)	Potentially Life Threatening (Grade 4)
Pain	Does not interfere with activity	Repeated use of non- narcotic pain reliever > 24 hours or interferes with activity	Any use of narcotic pain reliever or prevents daily activity	Emergency room (ER) visit or hospitalization
Tenderness	Mild discomfort to touch	Discomfort with movement	Significant discomfort at	ER visit or hospitalization
Erythema/Redness *	2.5 – 5 cm	5.1 – 10 cm	> 10 cm	Necrosis or exfoliative dermatitis
Induration/Swelling **	2.5 – 5 cm and does not interfere with	5.1 – 10 cm or interferes with activity	> 10 cm or prevents daily activity	Necrosis

* In addition to grading the measured local reaction at the greatest single diameter, the measurement should be recorded as a continuous variable.

** Induration/Swelling should be evaluated and graded using the functional scale as well as the actual measurement.

Vital Signs *	Mild (Grade 1)	Moderate(Grade 2)	Severe (Grade 3)	Potentially Life Threatening (Grade 4)
Fever (°C) **<br>(°F) **	38.0 – 38.4<br>100.4 – 101.1	38.5 – 38.9<br>101.2 – 102.0	39.0 – 40<br>102.1 – 104	> 40<br>> 104
Tachycardia - beats per minute	101 – 115	116 – 130	> 130	ER visit or hospitalization for arrhythmia
Bradycardia - beats per minute***	50 – 54	45 – 49	< 45	ER visit or hospitalization for arrhythmia
Hypertension (systolic) - mm Hg	141 – 150	151 – 155	> 155	ER visit or hospitalization for malignant hypertension
Hypertension (diastolic) - mm Hg	91 – 95	96 – 100	> 100	ER visit or hospitalization for malignant hypertension
Hypotension (systolic) – mm Hg	85 – 89	80 – 84	< 80	ER visit or hospitalization for hypotensive shock
Respiratory Rate – breaths per minute	17 – 20	21 – 25	> 25	Intubation

* Subject should be at rest for all vital sign measurements.
** Oral temperature; no recent hot or cold beverages or smoking.

*** When resting heart rate is between 60 – 100 beats per minute. Use clinical judgement when characterizing bradycardia among some healthy subject populations, for example, conditioned athletes.

**Systemic (General)**	**Mild (Grade 1)**	**Moderate(Grade 2)**	**Severe (Grade 3)**	**Potentially Life Threatening (Grade 4)**
Nausea/vomiting	No interference with activity or 1 – 2 episodes/24 hours	Some interference with activity or > 2 episodes/24 hours	Prevents daily activity, requires outpatient IV hydration	ER visit or hospitalization for hypotensive shock
Diarrhea	2 – 3 loose stools or < 400 gms/24 hours	4 – 5 stools or 400 – 800 gms/24 hours	6 or more watery stools or > 800gms/24 hours or requires outpatient IV hydration	ER visit or hospitalization
Headache	No interference with activity	Repeated use of non- narcotic pain reliever > 24 hours or some interference with [illegible]	Significant; any use of narcotic pain reliever or prevents daily activity	ER visit or hospitalization
Fatigue	No interference with activity	Some interference with activity	Significant; prevents daily activity	ER visit or hospitalization
Myalgia	No interference with activity	Some interference with activity	Significant; prevents daily activity	ER visit or hospitalization

**Systemic Illness**	**Mild (Grade 1)**	**(Moderate(Grade 2)**	**Severe (Grade 3)**	**Potentially Life Threatening (Grade 4)**
Illness or clinical adverse event (as defined according to applicable regulations)	No interference with activity	Some interference with activity not requiring medical [illegible]	Prevents daily activity and requires medical [illegible]	ER visit or hospitalization

Tables for Laboratory Abnormalities

The laboratory values provided in the tables below serve as guidelines and are dependent upon institutional normal parameters. Institutional normal reference ranges should be provided to demonstrate that they are appropriate.

Serum *	Mild (Grade 1)	Moderate (Grade 2)	Severe (Grade 3)	Potentially Life Threatening (Grade 4)**
Sodium – Hyponatremia mEq/L	132 – 134	130 – 131	125 – 129	< 125
Sodium – Hypernatremia mEq/L	144 – 145	146 – 147	148 – 150	> 150
Potassium – Hyperkalemia mEq/L	5.1 – 5.2	5.3 – 5.4	5.5 – 5.6	> 5.6
Potassium – Hypokalemia mEq/L	3.5 – 3.6	3.3 – 3.4	3.1 – 3.2	< 3.1
Glucose – Hypoglycemia mg/dL	65 – 69	55 – 64	45 – 54	< 45
Glucose – Hyperglycemia Fasting – mg/dL<br>Random – mg/dL	100 – 110<br>110 – 125	111 – 125<br>126 – 200	>125<br>>200	Insulin requirements or hyperosmolar coma
Blood Urea Nitrogen BUN	23 – 26	27 – 31	> 31	Requires dialysis
Creatinine – mg/dL	1.5 – 1.7	1.8 – 2.0	2.1 – 2.5	> 2.5 or requires dialysis
Calcium – hypocalcemia mg/dL	8.0 – 8.4	7.5 – 7.9	7.0 – 7.4	< 7.0
Calcium – hypercalcemia mg/dL	10.5 – 11.0	11.1 – 11.5	11.6 – 12.0	> 12.0
Magnesium – hypomagnesemia mg/dL	1.3 – 1.5	1.1 – 1.2	0.9 – 1.0	< 0.9
Phosphorous – hypophosphatemia mg/dL	2.3 – 2.5	2.0 – 2.2	1.6 – 1.9	< 1.6
CPK – mg/dL	1.25 – 1.5 x ULN***	1.6 – 3.0 x ULN	3.1 –10 x ULN	> 10 x ULN
Albumin – Hypoalbuminemia g/dL	2.8 – 3.1	2.5 – 2.7	< 2.5	--
Total Protein – Hypoproteinemia g/dL	5.5 – 6.0	5.0 – 5.4	< 5.0	--
Alkaline phosphate – increase by factor	1.1 – 2.0 x ULN	2.1 – 3.0 x ULN	□3.1 – 10 x ULN	> 10 x ULN
Liver Function Tests –ALT, AST increase by factor	1.1 – 2.5 x ULN	2.6 – 5.0 x ULN	5.1 – 10 x ULN	> 10 x ULN
Bilirubin – when accompanied by any increase in Liver Function Test increase by factor	1.1 – 1.25 x ULN	1.26 – 1.5 x ULN	1.51 – 1.75 x ULN	> 1.75 x ULN
Bilirubin – when Liver Function Test is normal; increase by factor	1.1 – 1.5 x ULN	1.6 – 2.0 x ULN	2.0 – 3.0 x ULN	> 3.0 x ULN
Cholesterol	201 – 210	211 – 225	> 226	---
Pancreatic enzymes – amylase, lipase	1.1 – 1.5 x ULN	1.6 – 2.0 x ULN	2.1 – 5.0 x ULN	> 5.0 x ULN

* The laboratory values provided in the tables serve as guidelines and are dependent upon institutional normal parameters. Institutional normal reference ranges should be provided to demonstrate that they are appropriate.

** The clinical signs or symptoms associated with laboratory abnormalities might result in characterization of the laboratory abnormalities as Potentially Life Threatening (Grade 4). For example. a low sodium value that falls within a grade 3 parameter (125-129 mE/L) should be recorded as a grade 4 hyponatremia event if the subject had a new seizure associated with the low sodium value.

***ULN" is the upper limit of the normal range.

Hematology *	Mild (Grade 1)	Moderate (Grade 2)	Severe (Grade 3)	Potentially Life Threatening (Grade 4)
Hemoglobin (Female) - gm/dL	11.0 – 12.0	9.5 – 10.9	8.0 – 9.4	< 8.0
Hemoglobin (Female) change from baseline value - gm/dL	Any decrease – 1.5	1.6 – 2.0	2.1 – 5.0	> 5.0
Hemoglobin (Male) - gm/dL	12.5 – 13.5	10.5 – 12.4	8.5 – 10.4	< 8.5
Hemoglobin (Male) change from baseline value – gm/dL	Any decrease – 1.5	1.6 – 2.0	2.1 – 5.0	> 5.0
WBC Increase - cell/mm^3	10,800 – 15,000	15,001 – 20,000	20,001 – 25, 000	> 25,000
WBC Decrease - cell/mm^3	2,500 – 3,500	1,500 – 2,499	1,000 – 1,499	< 1,000
Lymphocytes Decrease - cell/mm^3	750 – 1,000	500 – 749	250 – 499	< 250
Neutrophils Decrease - cell/mm^3	1,500 – 2,000	1,000 – 1,499	500 – 999	< 500
Eosinophils - cell/mm^3	650 – 1500	1501 - 5000	> 5000	Hypereosinophilic
Platelets Decreased - cell/mm^3	125,000 – 140,000	100,000 – 124,000	25,000 – 99,000	< 25,000
PT – increase by factor (prothrombin	1.0 – 1.10 x ULN**	□1.11 – 1.20 x ULN	1.21 – 1.25 x ULN	> 1.25 ULN
PTT – increase by factor (partial thromboplastin	1.0 – 1.2 x ULN	1.21 – 1.4 x ULN	1.41 – 1.5 x ULN	> 1.5 x ULN
Fibrinogen increase - mg/dL	400 – 500	501 – 600	> 600	--
Fibrinogen decrease - mg/dL	150 – 200	125 – 149	100 – 124	< 100 or associated with gross bleeding or disseminated intravascular [illegible]

* The laboratory values provided in the tables serve as guidelines and are dependent upon institutional normal parameters. Institutional normal reference ranges should be provided to demonstrate that they are appropriate.

** "ULN" is the upper limit of the normal range.

Urine *	Mild (Grade 1)	Moderate (Grade 2)	Severe (Grade 3)	Potentially Life Threatening (Grade 4)
Protein	Trace	1+	2+	Hospitalization or dialysis
Glucose	Trace	1+	2+	Hospitalization for hyperglycemia
Blood (microscopic) – red blood cells per high power field (rbc/hpf)	1 - 10	11 – 50	> 50 and/or gross blood	Hospitalization or packed red blood cells (PRBC) transfusion

* The laboratory values provided in the tables serve as guidelines and are dependent upon institutional normal parameters. Institutional normal reference ranges should be provided to demonstrate that they are appropriate.

Appendix III: Justification for proposed ACTIMMUNE® (Interferon gamma-1b) dosing in: COMIRB #14-2231: Effect of IFN- γ-1b on Innate Immune cells

The planned dosing of ACTIMMUNE® is largely consistent with that already approved in the ACTIMMUNE® BLA (s) and, where slightly different (single-dose of 100mcg/ m^2), is supported by a substantial body of clinical safety data as summarized below:

	**ACTIMMUNE® Dosing**	**Planned Dosing : COMIRB #14-2231**
Age/Gender	All ages , male and female	18-60 years of age, male and female
Route of A	SC	SC
Individualised dosing	per m^2	per m^2
Maximum daily dose	50mcg/m^2 3 times weekly	Single dose 25-100mcg/m^2
		Multiple dose 50mcg/m^2 3 times weekly
Population	CGD and malignant osteopetrosis patients	Healthy Subjects

Maximum Tolerated Dose (MTD)

The original Phase 1 clinical development of IFN-γ 1b was conducted in oncology/AIDS patients and maximum tolerated doses based on IM or IV administration were established as follows:

Maximum Tolerated Dose (MTD) of IFN-γ lb in Multiple-Dose, Phase 1 Clinical Trials in Patients with Advanced Malignancy or AIDS

Treatment Regimen	MTD (mcg/m^2/day)
Daily IM injection (6 weeks)	250-500
Daily 24-hour IV infusion (4 weeks)	10-250
Daily 6-hour IV infusion (2 weeks)	250-500
1-hour IV infusion three times weekly (6 weeks)	500-1000

Subsequently, daily doses of 100mcg/m^2 IFN-γ 1b by the SC route of administration was chosen based on an NCI study (Maluish AE et al 1988) comparison of immunological activity (including enhanced superoxide/hydrogen peroxide production) of different doses and routes of administration in melanoma patients. Subcutaneous (SC) administration was compared with IM administration using the 100 mcg/m^2 dose. SC administration resulted in enhanced hydrogen peroxide production and Fc receptor expression by monocytes. A comparison of two schedules, daily and three times weekly, suggested that monocyte activation may persist for up to 72 hours after IFN-γ 1b administration.

Of the doses tested, 100 mcg/m^2 administered daily or three times weekly appeared to be the most effective biological response modifier (BRM) regimen, and due to ease of administration, the SC route was preferred.

Healthy Subjects

The pharmacokinetics of exogenous IFN-γ 1b in healthy male only subjects at single and multiple doses of 100mcg/m^2 were included in the BLA for approval in CGD and are described in the prescribing information for ACTIMMUNE® (Legacy Studies).

The safety and tolerability in both these single-dose and multiple dose studies was as follows:

Study 10091g ‘Pharmacokinetic deposition of recombinant human interferon-gamma following intravenous, subcutaneous and intramuscular administration in normal male volunteers’.
This study involved 24 healthy male subjects that received single administrations of IFN-γ 1b at 100mcg/m^2 by different parenteral rotes of administration. No serious or life-threatening toxicities due to IFN-γ 1b were observed. The most commonly observed toxicities were mild to moderate constitutional symptoms consisting of chills, myalgia and headache. Fever and nausea were observed less frequently. Administration of IFN-γ 1b was well tolerated.

Study 10125g ‘Pharmacokinetic deposition of recombinant human interferon-gamma following chronic subcutaneous administration in normal male volunteers’. This study involved 38 healthy male subjects that received multiple administrations of IFN-γ 1b at 100mcg/m^2 either daily or three times weekly.
No serious or life-threatening toxicities due to IFN-γ 1b were observed. The most commonly observed toxicities were mild to moderate fever, chills, fatigue, myalgia and headache. Fever and nausea were observed less frequently. Administration of IFN-γ 1b was well tolerated. Daily administration of IFN-γ 1b had a higher incidence of constitutional symptoms compared to three times weekly injections. There were four subject study withdrawals on each treatment regimen; seven cases relating to headache, muscle aches, fatigue, nausea, light-headedness, mild diarrhoea and one case relating to intercurrent illness (sore throat).

More recently, a study in 40 healthy subjects assessing the impact of dose titration on FLS at escalating doses of 15, 30 and 50mcg/m^2 was reported (Devane JG et al 2014).The subjects in this study offered a broader range of subject demographics than the Legacy PK BLA studies including both male and female subjects.

While a total of 15 subjects discontinued the study prematurely, only 4 subject discontinuations were due to FLS or other AEs (neutropenia, elevated white blood cell (WBC) count and traumatic brain injury with loss of consciousness (also a Serious Adverse Event [SAE] judged possibly related to study drug).

A population PK model based on the combined Legacy and Devane et al 2014 studies was successfully developed and the impact of certain patient/procedure covariates was assessed. The following were some key conclusions:

- A base 2 compartment PK model best fitted the data with median CL/F estimated as 28.6L/h.
- Neither dose (15 to 100mcg/m^2), race nor gender had any significant impact on CL/F - this indicates dose linearity in PK over the 15 to 100mcg/m^2 range and gender/race independent dosing.
- A 20% lower CL/F was associated with SC injection in the abdomen compared with thigh. This mirrors similar patterns with other injected therapeutic proteins and most likely reflects a different bioavailability (F) from different injection sites.
- BSA was a significant covariate for CL/F validating the current and planned body mass based dosage regimen.
- Age was a marginally significant covariate. The range from 18 to 55 years appeared to be associated with an age related increase in CL/F. Given the bias in the planned study in favour of younger patients, this suggests less likelihood of any unexpected accumulation.

Based on the Population PK model, simulated PK profiles for both standard 50 mcg/m^2 and the proposed 100mcg/m^2 dosing of IFN-γ 1b following SC injections three times weekly (TIW) are shown below. It is noteworthy that based on a 3 times weekly injection regimen, there is no accumulation and the differences between the doses are principally different C_{Max} exposures.

50 mcg/m2 TIW

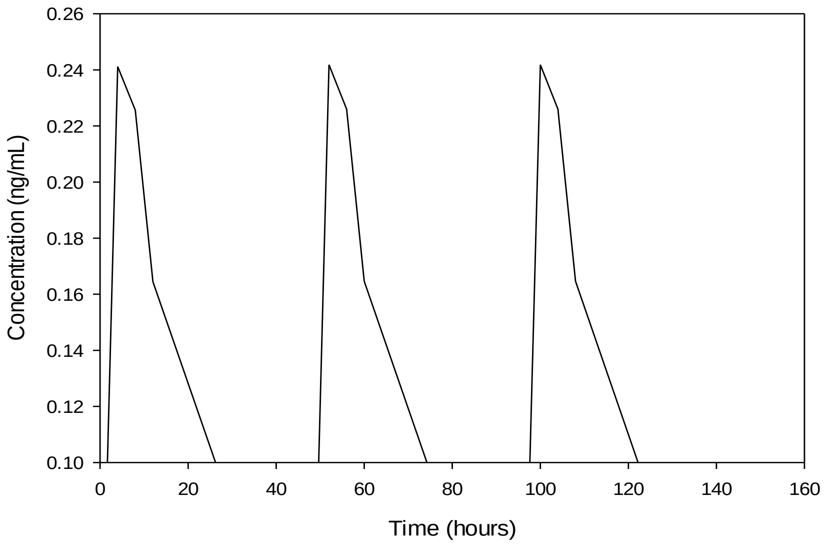

100 mcg/m2 TIW

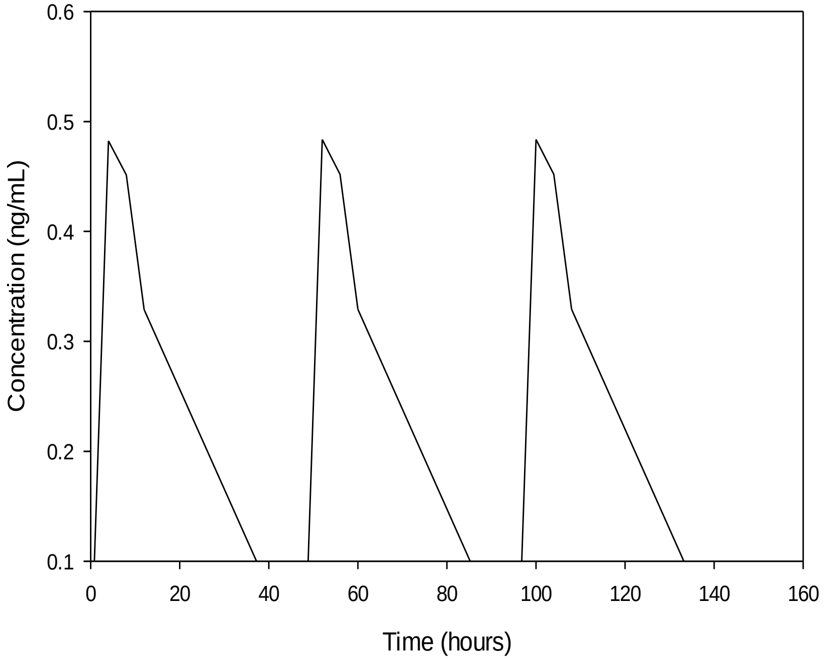

Chronic Granulomatous Disease (CGD) and Prescribing Information

Based on the immunological profile of IFN-γ 1b and its apparent activity to boost innate and adaptive antimicrobial activity including the oxidative burst in neutrophils, an *in-vitro* treatment with IFN-γ 1b of phagocytes from CGD patients (who have a genetic defect in their NADPH oxidase enzyme) suggested an enhanced superoxide production (Ezekowitz RA et al 1987). Subsequently, a study of IFN-γ 1b treatment in 3 CGD patients showed improved oxidative burst/metabolism and superoxide production as well as improved phagocyte bactericidal activity. The IFN-γ 1b doses in this study were 10 or 50mcg/m^2 by SC injection daily or every other day and were well tolerated (Sechler JM et al 1988).

Thereafter a pivotal Phase 3 study in 128 CGD patients using 50mcg/m^2 SC three times weekly of IFN-γ 1b or placebo established the clinical benefit of prophylactic therapy with IFN-γ 1b in CGD and its good tolerability and was the basis of regulatory approval in 1990 (Ezekowitz RA et al 1991). The typical labelled AE profile is dominated by relatively high frequency constitutional; side effects such as fever, headache, myalgia, chills, tiredness and fatigue (often referred to as Flu like Symptoms).

From the ACTIMMUNE Prescribing Information:

The most common adverse events observed in patients with CGD are shown in the following table:

Percent of Patients **Clinical Toxicity**	***ACTIMMUNE*** **CGD (n=63)**	**Placebo CGD (n=65)**
Fever	52	28
Headache	*33*	*9*
Rash	*17*	*6*
Chills	*14*	*0*
Injection site erythema or tenderness	*14*	*2*
Fatigue	*14*	*11*
Diarrhea	*14*	*12*
Vomiting	*13*	*5*
Nausea	*10*	*2*
Myalgia	*6*	*0*
Arthralgia	*2*	*0*
Injection site pain	*0*	*2*

Bone Marrow Toxicity

Reversible neutropenia and thrombocytopenia that can be severe and may be dose related have been observed during ACTIMMUNE® therapy. Caution should be exercised when administering ACTIMMUNE® to patients with myelosuppression.

Hepatic Toxicity

Elevations of AST and /or ALT (up to 25-fold) have been observed during ACTIMMUNE® therapy. The incidence appeared to be higher in patients less than 1 year of age compared to older children. The transaminase elevations were reversible with reduction in dosage or interruption of ACTIMMUNE® treatment. Patients beginning ACTIMMUNE® treatment before age one year should receive monthly assessments of liver function. (If severe hepatic enzyme elevations develop, ACTIMMUNE® dosage should be modified).

Patient clinical exposure to 200mcg (approx. 100mcg/m^2)

Although 50mcg/m^2 three times weekly by SC injection was the approved dose in CGD, there remains the possibility that higher doses, particularly the 100mcg/m^2 dose, previously identified as optimal in the dose finding studies, might be superior in other indications. Thus, a number of Phase

2/3 clinical trials have been conducted in other indications at doses equivalent to 100mcg/m^2 i.e. 200mcg three times weekly by SC injection.

A direct comparison of 2 doses of IFN-γ 1b was conducted in a large Phase 2 trial in liver disease patients (Appendix 1). This controlled trial was a randomized, double-blind, placebo-controlled study evaluating two doses of SC IFN-γ 1b (100 μg and 200 μg) in patients with chronic hepatitis C virus (HCV) infection and advanced liver fibrosis or compensated cirrhosis (Polros PJ et al 2007).

IFN-γ 1b appeared to be well tolerated in patients with advanced HCV-associated liver fibrosis. The incidence of overall adverse events deemed by the investigators to be possibly or probably related to study drug was similar across all 3 treatment arms: 96% (151/157) in the IFN-γ 1b 200-μg arm, 98% (165/169) in the IFN-γ 1b 100-μg arm, and 94% (153/162) in the placebo arm. Fever, rigors, influenza-like illness, myalgia, arthralgia, headache, and fatigue occurred more frequently in patients receiving IFN-γ 1b than in those receiving placebo. Fourteen patients died during the study: 5 each in the IFN-γ 1b 200μg and 100-μg arms, and 4 in the placebo arm. Events leading to death were similarly distributed among the 3 treatment arms, and were related primarily to cardiac disease, progression of liver disease, and multi-organ failure. Greater percentages of patients in the 200-μg IFN-γ 1b and 100-μg IFN-γ 1b groups than in the placebo group experienced absolute neutrophil counts and total WBC counts at the Grade 3 toxicity level: 15%, 9%, and 3%, respectively, for Grade 3 absolute neutrophil counts and 6%, 5%, and 1%, respectively, for Grade 3 WBC counts.

Summary of Treatment-Emergent Adverse Events in ≥5% of Patients with Liver Fibrosis Treated with IFN-γ 1b or Placebo

Adverse Event (AE)<br>Body System/Organ Class)	Number (%) of Patients with AEs		
	IFN-γ 1b 200μg (N = 157)	IFN-γ 1b 100μg (N = 169)	Placebo (N = 162)
Number of Patients with at Least One AE	156 (99)	168 (99)	161 (99)
BLOOD AND LYMPHATIC SYSTEM DISORDERS	32 (20)	25 (15)	18 (11)
Neutropenia	10 (6)	9 (5)	6 (4)
Leukopenia NOS	8 (5)	4 (2)	3 (2)
EYE DISORDERS	29 (18)	22 (13)	22 (14)
Vision blurred	8 (5)	3 (2)	8 (5)
GASTROINTESTINAL DISORDERS	100 (64)	102 (60)	90 (56)
Nausea	36 (23)	42 (25)	41 (25)
Diarrhoea NOS	24 (15)	24 (14)	31 (19)
Abdominal pain upper	23 (15)	18 (11)	20 (12)
Dyspepsia	19 (12)	23 (14)	17 (10)
Abdominal pain NOS	13 (8)	12 (7)	15 (9)
Vomiting NOS	8 (5)	11 (7)	13 (8)
Abdominal distension	5 (3)	14 (8)	9 (6)
Constipation	11 (7)	6 (4)	7 (4)
Ascites	9 (6)	6 (4)	5 (3)
Flatulence	5 (3)	8 (5)	3 (2)
GENERAL DISORDERS AND ADMINISTRATION SITE	141 (90)	148 (88)	115 (71)

Adverse Event (AE) Body System/Organ Class)	Number (%) of Patients with AEs		
	IFN-γ 1b 200μg (N = 157)	IFN-γ 1b 100μg (N = 169)	Placebo (N = 162)
CONDITIONS			
Fatigue	71 (45)	65 (38)	61 (38)
Rigors	75 (48)	52 (31)	21 (13)
Pyrexia	54 (34)	52 (31)	21 (13)
Influenza-like illness	35 (22)	32 (19)	23 (14)
Pain NOS	32 (20)	31 (18)	17 (10)
Edema peripheral	19 (12)	30 (18)	18 (11)
Injection site bruising	16 (10)	12 (7)	13 (8)
Weakness	14 (9)	11 (7)	7 (4)
Injection site erythema	13 (8)	12 (7)	6 (4)
Malaise	10 (6)	10 (6)	7 (4)
Chest pain	13 (8)	6 (4)	6 (4)
Lethargy	8 (5)	1 (1)	5 (3)
HEPATOBILIARY DISORDERS	25 (16)	20 (12)	15 (9)
Hepatomegaly	9 (6)	10 (6)	5 (3)
INFECTIONS AND INFESTATIONS	54 (34)	89 (53)	69 (43)
Nasopharyngitis	9 (6)	16 (9)	24 (15)
Upper respiratory tract infection NOS	11 (7)	19 (11)	15 (9)
Sinusitis NOS	10 (6)	18 (11)	12 (7)
Bronchitis NOS	8 (5)	8 (5)	11 (7)
Urinary tract infection NOS	4 (3)	12 (7)	10 (6)
Influenza	1 (1)	10 (6)	6 (4)
INJURY, POISONING AND PROCEDURAL COMPLICATIONS	16 (10)	21 (12)	22 (14)
Post procedural pain	2 (1)	4 (2)	9 (6)
METABOLISM AND NUTRITION DISORDERS	28 (18)	36 (21)	35 (22)
Anorexia	7 (4)	11(7)	6 (4)
Hypertriglyceridemia	9 (6)	2 (1)	6 (4)
Appetite decreased NOS	3 (2)	5 (3)	8 (5)
MUSCULOSKELETAL AND CONNECTIVE TISSUE DISORDERS	108 (69)	107 (63)	90 (56)
Arthralgia	48 (31)	52 (31)	40 (25)
Myalgia	50 (32)	52 (31)	34 (21)
Muscle cramps	40 (25)	24 (14)	12 (7)
Back pain	15 (10)	21 (12)	28 (17)
Pain in limb	10 (6)	11 (7)	10 (6)
NERVOUS SYSTEM DISORDERS	111 (71)	124 (73)	98 (60)
Headache NOS	87 (55)	101 (60)	74 (46)
Dizziness	19 (12)	31 (18)	17 (10)
Tremor	8 (5)	9 (5)	5 (3)
Disturbance in attention	5 (3)	6 (4)	9 (6)
PSYCHIATRIC DISORDERS	77 (49)	81 (48)	82 (51)
Insomnia	38 (24)	47 (28)	32 (20)
Depression	22 (14)	27 (16)	32 (20)

Adverse Event (AE) Body System/Organ Class)	Number (%) of Patients with AEs		
	IFN-γ 1b 200μg (N = 157)	IFN-γ 1b 100μg (N = 169)	Placebo (N = 162)
Irritability	20 (13)	16 (9)	19 (12)
Anxiety NEC	9 (6)	17 (10)	13 (8)
Confusion	5 (3)	5 (3)	8 (5)
RESPIRATORY, THORACIC, AND MEDIASTINAL DISORDERS	67 (43)	72 (43)	67 (41)
Cough	19 (12)	32 (19)	21 (13)
Pharyngolaryngeal pain	11 (7)	16 (9)	15 (9)
Dyspnea NOS	14 (9)	14 (8)	11 (7)
Epistaxis	13 (8)	12 (7)	12 (7)
Nasal congestion	9 (6)	12 (7)	13 (8)
Sinus congestion	1 (1)	12 (7)	7 (4)
Rhinorrhea	4 (3)	9 (5)	3 (2)
SKIN AND SUBCUTANEOUS TISSUE DISORDERS	63 (40)	54 (32)	59 (36)
Pruritus NOS	25 (16)	14 (8)	15 (9)
Rash NOS	14 (9)	10 (6)	14 (9)
Sweating increased	9 (6)	10 (6)	6 (4)
Dry skin	8 (5)	6 (4)	5 (3)

Adverse events resulting in treatment discontinuation occurred in 14% (22/157) of patients in the IFN-γ 1b 200-μg arm, 11% (18/169) in the IFN-γ 1b 100-μg arm and 6% (9/162) in the placebo arm; however, no single adverse event stands out as the predominant reason for discontinuation of study treatment. The frequency of serious adverse events was similar across all treatment arms: 13% (20/157) of patients in the IFN-γ 1b 200-μg arm, 14% (24/169) in the IFN-γ 1b 100-μg arm, and 14% (23/162) in the placebo arm.

The most recent and largest efficacy/safety trial at the 200mcg IFN-γ 1b dosage was Study GIPF-007 which was a Phase 3, randomized, double-blind, placebo-controlled, multinational, safety and efficacy study of IFN-γ 1b in patients with IPF. A total of 826 patients at 81 centres in Europe and North America were randomly assigned (2:1) to receive 200 μg of SC IFN-γ 1b or placebo equivalent three times weekly. The primary efficacy outcome measure was survival time from date of randomization. The AEs associated with IFN-γ 1b were generally consistent with prior clinical experience, including constitutional symptoms, neutropenia, and elevations in AST and ALT .The safety results in this study were generally consistent with prior clinical experience of IFN-γ 1b and suggest that long-term therapy with IFN-γ 1b is generally well tolerated at this dosage.

In addition, a variety of other clinical trials with IFN-γ 1b at 200mcg SC injection exposure are listed below. The clinical safety and tolerability at the 200mcg SC dose appeared consistent with the labelled information.

Clinical Trials with IFN-γ 1b at 200mcg SC Injection Exposure

Reference	Sponsor	Trial Phase	Indication	Number of Patients	Dosing Regimen
Meyskens 1995	*Sponsor: Southwest Oncology Group; Source: Genentech*	*Randomized Phase 3*	*Cutaneous melanoma Stage 2 or 3*	*137*	*200 μg/d × 1 yr (n = 137) vs. no treatment (n = 147)*
Dawson 2009	*Investigator-sponsored*	*Randomized, 3 treatment arms*	*Bilateral cavitary tuberculosis*	*89*	*DOTS alone or with either aerosol or SC IFN-γ 1b 200 μg TIW, 16 weeks*
GICF-002	*InterMune*	*Randomized, double-blind, placebo-controlled, phase 1/2*	*Mild-to-moderate CF*	*38*	*IFN-γ 1b (50 μg/m2 or 100 μg/m2) or placebo TIW x 4 wks*
GINHL-001	*InterMune*	*Schedule- and dose-escalating, open-label, phase 1/2*	*Relapsed or progressive low-grade/ follicular NHL*	*24; 6 in each of 4 groups*	*IFN-γ 1b 100 μg or 200 μg TIW plus IV rituximab 375 mg/m2 ow x 4 weeks*
Muir 2006	*Investigator-sponsored*	*Open-label*	*Chronic hepatitis C*	*20*	*IFN-γ 1b 200 μg TIW SC for 24 weeks*
Li 1990	*Sponsor and source: Genentech*	*Randomized placebo-controlled, open-label*	*Ragweed-allergic rhinitis*	*29*	*200 μg/d (n = 14) vs. 20 μg/d (n = 15) vs. placebo (n = 15)*
Bitran 1995	*Sponsor: unknown; source: National Cancer Institute*	*Open-label*	*Patients with partial response to chemo-therapy for extensive small cell lung cancer*	*30*	*200 μg/d*
Ghanei 2006		*Open-label, case-control groups*	*Bronchiolitis in mustard gas-exposed patients*	*36*	*Inhaled Felixotide and Serevent OR IFN-γ 1b 200 μg TIW SC + prednisolone 7.5mg*

Experience of single-dose escalation in Friedreich's Ataxia patients

A single dose escalation study in adult FA patients has been conducted in Rome, Italy in the recent past but is not yet reported.

**EudraCT Number:** 2012-001881-14 **Sponsor Protocol Number:** GIFT/1 **Start Date*:** 2013-03-01	
**Sponsor Name:** AZIENDA UNIVERSITARIA POLICLINICO UMBERTO I DI ROMA	
**Full Title:** A phase II clinical trial to evaluate the safety and efficacy of interferon gamma in elevating frataxin levels in Friedreich ataxia patients	
**Medical condition:** Friedreich's Ataxia	
**Population Age:** Adults	**Gender:** Male, Female
**Trial protocol:** IT *(Ongoing)*	
**Trial results:** *(No results available)*	

The dose escalation was from 100mcg to 150mcg to 200mcg by single SC injection. The key measurement was frataxin protein in PMBC's.

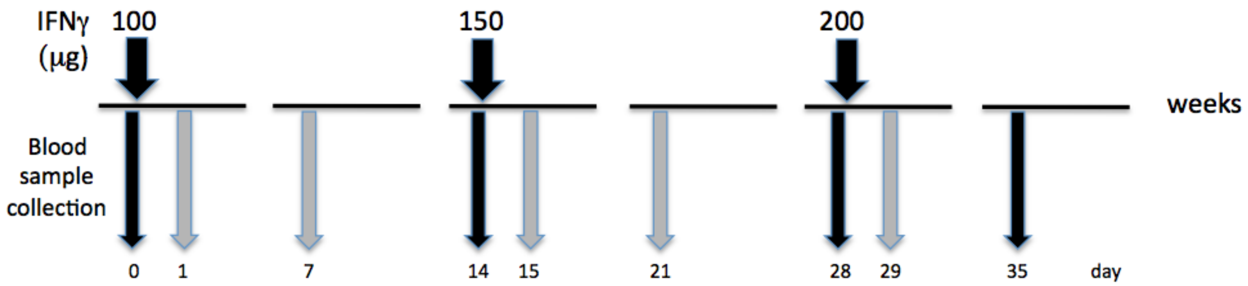

The study has not yet been reported but a summary of the safety/tolerability has been received from the investigators:

	Pz	After 100 mcg N	Action taken	After 150 mcg	Action taken	After 200 mcg	Action taken
1	33 F	None	none	Hyperthermia (37.5°C) for a few hrs, spontaneous resolution	None	Light headache for a few hrs, spontaneous resolution	none
2	33 F	None	none	none	none	none	none
3	32 M	None	none	none	none	none	none
4	21 F	None	none	none	none	none	none
5	26 F			Hyperthermia (37.6°C) for a few hrs, resolved after paracetamol	paracetamol 1 g xos	Fever (38.2°C) for a few hrs, resolved after paracetamol	paracetamol 1 g xos
6	29 M	Fever (38.2°C) for a few hrs, resolved after paracetamol	paracetamol 1 g xos	none	none	none	none

7	30 F	Fever (38.7°C) for a few hrs, resolved after paracetamol	paracetamol 1 g xos	none	none	none	none
8	29 F	None	none	Hyperthermia (37.2°C) for a few hrs, spontaneous resolution	none	none	none
9	26 M	Hyperthermia (37.5°C) for a few hrs, resolved after paracetamol	paracetamol 1 g xos	Hyperthermia (37.5°C) for a few hrs, resolved after paracetamol, nausea, vomiting	paracetamol 1 g xos metoclopramide 10mg, 2 ml i.m.	Fever (38.0°C) for a few hrs, resolved after paracetamol, nausea, vomiting	paracetamol 1 g xos metoclopramide 10mg, 2 ml i.m.
10	35 F	None	none	none		Fever (38.4°C) for a few hrs, resolved after paracetamol	paracetamol 1 g xos

Overall, the acute tolerability was acceptable and was consistent with the labelled safety for ACTIMMUNE®.

CONCLUSION

This document summarizes the substantial clinical experience in both healthy subjects and various patient populations at doses at or equivalent (200mcg) to that proposed for this study (up to 100mcg/m^2 single dose) The clinical evidence to support acute tolerability in healthy subjects at the higher dose combined with the apparent predictability of ACTIMMUNE® PK in healthy subjects supports the proposed dosing in this study.

References

Devane JG, M Martin, M Matson. 2014. “A short 2 week dose titration regimen reduces the severity of flu-like symptoms with initial interferon gamma-1b treatment.” *Curr Med Res Opin.* 30(6):1179-1187.

Ezekowitz RA, S Orkin, P Newburger. 1987. “Recombinant interferon gamma augments phagocyte superoxide production and X-chronic granulomatous disease gene expression in X-linked variant chronic granulomatous disease.” *J Clin Invest.* 80 (4): 1009 – 1016.

Ezekowitz RA. 1991. “A Controlled Trial of Interferon Gamma to Prevent Infection in Chronic Granulomatous Disease -The International Chronic Granulomatous Disease Cooperative Study Group.” *N Engl J Med.* 324:509-516.

Maluish AE, W Urba, D Longo, W Overton, D Coggin, E Crisp, R Williams, S Sherwin, K Gordon, R Steis. 1988, “The determination of an immunologically active dose of interferon-gamma in patients with melanoma.” *JCO.* 434-45.

Polros PJ, Jeffers L, Afdhal N et al. 2007. ‘‘Final results of a double-blind, placebo-controlled trial of the antifibrotic efficacy of interferon-gamma1b in chronic hepatitis C patients with advanced fibrosis or cirrhosis.’’ Hepatology. 45(3):569-578

Sechler JM, H Malech, C White, J Gallin. 1988. “Recombinant human interferon-gamma reconstitutes defective phagocyte function in patients with chronic granulomatous disease of childhood.” *Proc Natl Acad Sci U S A.* 85(13):4874-4878.

Appendix IV: Enrollment Timeline for both Single Dose (SD) and Steady State (SS) Cohorts:

Cohort	Timepoint (Weeks)	Subject #	IFN-γ Dose (mcg/m^2)	Total blood volume drawn at that visit (mL)
Start of Single Dose Cohort	1	Subject 1 SD begins	10	204
	2	Subject 2 SD begins	10	204
	3	Subject 3 SD begins	10	204
	4	Subject 4 SD begins	10	204
	5	Subject 1	25	204
	6	Subject 2	25	204
	7	Subject 3	25	204
	8	Subject 4	25	204
	9	Subject 1	50	204
	10	Subject 2	50	204
	11	Subject 3	50	204
	12	Subject 4	50	204
	13	Subject 1	100	204
	14	Subject 2	100	204
	15	Subject 3	100	204
	16	Subject 4	100	204
	17	Subject 1 post 30-day ADA	n/a	3
		Subject 5 SD begins	10	204
	18	Subject 2 post 30-day ADA	n/a	3
		Subject 6 SD begins	10	204
	19	Subject 3 post 30-day ADA	n/a	3
		Subject 7 SD begins	10	204
	20	Subject 4 post 30-day ADA	n/a	3
		Subject 8 SD begins	10	204
	21	Subject 5 SD	25	204
	22	Subject 6 SD	25	204
	23	Subject 7 SD	25	204
	24	Subject 8 SD	25	204
	25	Subject 5 SD	50	204
	26	Subject 6 SD	50	204
	27	Subject 7 SD	50	204
	28	Subject 8 SD	50	204
	29	Subject 5 SD	100	204
	30	Subject 6 SD	100	204
	31	Subject 7 SD	100	204

	32	Subject 5 SD post 30-day ADA	n/a	3
		Subject 8 SD	100	204
	33	Subject 6 SD post 30-day ADA	n/a	3
		Subject 9 SD	10	204
	34	Subject 10 SD	10	204
	35	Subject 7 SD post 30-day ADA	n/a	3
Start of Steady State Cohort (Neutrophil analysis)	35	Subject 1 of SS neutrophil cohort starts	50	≤ 156 over 2 weeks
	36	Subject 8 SD post 30-day ADA	n/a	3
	37	Subject 9 SD	25	201
	38	Subject 10	25	201
	39	Subject 2 of SS neutrophil cohort starts	50	≤ 156 over 2 weeks
	40	Subject 1 SS post 30-day ADA	n/a	3
	41	Subject 9 SD	50	201
	42	Subject 10 SD	50	201
	43	Subject 3 SS neutrophil starts	50	≤ 156 over 2 weeks
	43	Subject 2 SS post 30-day ADA	n/a	3
	44	-	-	-
	45	Subject 9 SD	100	201
	46	Subject 10 SD	100	201
	47	Subject 3 SS post 30-day ADA	n/a	3
	47	Subject 4 SS neutrophil starts	50	≤ 156 over 2 weeks
	48	-	-	-
	49	Subject 9 SD post 30-day ADA	n/a	3
	49	Subject 5 SS neutrophil starts	50	≤ 156 over 2 weeks
	50	Subject 10 SD post 30-day ADA	n/a	3
	51	Subject 4 SS post 30-day ADA	na/	3
	51	Subject 6 SS neutrophil starts	50	≤ 156 over 2 weeks
	52	-	-	-
	53	Subject 5 SS post 30-day ADA	n/a	3
	53	Subject 7 SS neutrophil starts	50	≤ 156 over 2 weeks
	54	-	-	-
	55	Subject 6 SS post 30-day ADA	n/a	3

56	-	-	-
57	Subject 7 SS post 30-day ADA	n/a	3
57	Subject 8 SS neutrophil starts	50	≤ 156 over 2 weeks
58	-	-	-
59	Subject 9 SS neutrophil starts	50	≤ 156 over 2 weeks
60	-	-	-
61	Subject 8 SS post 30-day ADA	n/a	3
61	Subject 10 SS neutrophil starts	50	≤ 156 over 2 weeks
62	-	-	-
63	Subject 9 SS post 30-day ADA	n/a	3
63	Subject 11 SS monocyte starts	50	≤ 156 over 2 weeks
64	Subject 10 SS post 30-day ADA	n/a	3
65	Subject 12 SS monocyte starts	50	≤ 156 over 2 weeks
66			
67	Subject 11 post 30-day ADA	n/a	3
67	Subject 13 SS monocyte starts	50	≤ 156 over 2 weeks
68			
69	Subject 12 post 30-day ADA	n/a	3
69	Subject 14 SS monocyte starts	50	≤ 156 over 2 weeks
70	-	-	-
71	Subject 13 post 30-day ADA	n/a	3
71	Subject 15 SS monocyte starts	50	≤ 156 over 2 weeks
72	-	-	-
73	Subject 14 post 30-day ADA	n/a	3
73	Subject 16 SS monocyte starts	50	≤ 156 over 2 weeks
74	-	-	-
75	Subject 15 post 30-day ADA	n/a	3
75	Subject 17 SS monocytes starts	50	≤ 156 over 2 weeks
76	-	-	-
77	Subject 16 post 30-day ADA	n/a	3
77	Subject 18 SS monocyte starts	50	≤ 156 over 2 weeks
78	-	-	-

79	Subject 17 post 30-day ADA	n/a	3
79	Subject 19 SS monocyte starts	50	≤ 156 over 2 weeks
80	-	-	-
81	Subject 18 post 30-day ADA	n/a	3
81	Subject 20 SS monocyte starts	50	≤ 156 over 2 weeks
82	-	-	-
83	Subject 19 post 30-day ADA	n/a	3
84	-	-	-
85	Subject 20 post 30-day ADA	n/a	3
